# CREB3L2-ATF4 heterodimerization defines a transcriptional hub of Alzheimer’s disease gene expression linked to neuropathology

**DOI:** 10.1126/sciadv.add2671

**Published:** 2023-03-03

**Authors:** Cláudio Gouveia Roque, Kyung Min Chung, Ethan P. McCurdy, Radhika Jagannathan, Lisa K. Randolph, Krystal Herline-Killian, Jimena Baleriola, Ulrich Hengst

**Affiliations:** ^1^ The Taub Institute for Research on Alzheimer’s Disease and the Aging Brain, Vagelos College of Physicians and Surgeons, Columbia University, New York, NY, USA.; ^2^ Integrated Program in Cellular, Molecular, and Biomedical Studies, Vagelos College of Physicians and Surgeons, Columbia University, New York, NY, USA.; ^3^ Division of Aging and Dementia, Department of Neurology, Vagelos College of Physicians and Surgeons, Columbia University, New York, NY, USA.; ^4^ Doctoral Program in Neurobiology and Behavior, Columbia University, New York, NY, USA.; ^5^ Achucarro Basque Center for Neuroscience, Leioa, Spain.; ^6^ IKERBASQUE Basque Foundation for Science, Bilbao, Spain.; ^7^ Department of Cell Biology and Histology, University of the Basque Country, Leioa, Spain.; ^8^ Department of Pathology and Cell Biology, Vagelos College of Physicians and Surgeons, Columbia University, New York, NY, USA.

## Abstract

Gene expression is changed by disease, but how these molecular responses arise and contribute to pathophysiology remains less understood. We discover that β-amyloid, a trigger of Alzheimer’s disease (AD), promotes the formation of pathological CREB3L2-ATF4 transcription factor heterodimers in neurons. Through a multilevel approach based on AD datasets and a novel chemogenetic method that resolves the genomic binding profile of dimeric transcription factors (ChIPmera), we find that CREB3L2-ATF4 activates a transcription network that interacts with roughly half of the genes differentially expressed in AD, including subsets associated with β-amyloid and tau neuropathologies. CREB3L2-ATF4 activation drives tau hyperphosphorylation and secretion in neurons, in addition to misregulating the retromer, an endosomal complex linked to AD pathogenesis. We further provide evidence for increased heterodimer signaling in AD brain and identify dovitinib as a candidate molecule for normalizing β-amyloid–mediated transcriptional responses. The findings overall reveal differential transcription factor dimerization as a mechanism linking disease stimuli to the development of pathogenic cellular states.

## Teaser

A unique transcription factor pair drives gene expression changes connected to hallmarks of Alzheimer’s decline.

## INTRODUCTION

Alzheimer’s disease (AD) is a progressive neurodegenerative disorder with an increasing worldwide prevalence. The preclinical phase of AD, which can last 10 to 20 years, is characterized by the gradual accumulation of β-amyloid and tau aggregates in the brain, together with neuroinflammation and synaptic alterations (*1*). Several lines of evidence indicate that β-amyloid deposition precedes and accelerates tau pathology, the latter correlating with the onset of cognitive decline (*1*–*3*). Concurrently, gene expression changes across specific pathways tied to pathophysiology are observed (*4*–*7*), highlighting an important role for altered transcriptional regulators in AD. What causes these changes, how they interact with β-amyloid and tau pathologies, and whether they are drivers of disease or a response to it remain, however, unclear.

Far from a binary on/off switch, gene expression has emerged as a nuanced, dynamic, and collaborative process involving various transcriptional layers (*8*). It follows that AD-associated gene expression changes can only be fully explained in light of this regulatory interdependency. Transcription factor (TF) dimerization, a common feature among many TF families, is a salient but mostly overlooked case in point, in that it can generate enormous variability in DNA binding specificities and transcriptional activities (*9*–*12*). Crucially, network analyses of gene coexpression profiles typically used in AD research are not designed to capture these synergistic TF combinations, resulting in a fragmented mechanistic understanding of the gene programs underlying AD progression and, most likely, missed therapeutic opportunities.

Here, we report the discovery and characterization of a pathological TF heterodimer, CREB3L2-ATF4, linked to AD pathogenesis. CREB3L2-ATF4 heterodimerization is potentiated by β-amyloid in neurons, and we confirmed that their association is enriched in AD brain. To probe its role in pathogenesis, we engineered a new chemogenetic methodology, ChIPmera, which resolves the DNA binding specificities of dimeric TFs in their cellular context. We found that the CREB3L2-ATF4 heterodimer regulates a transcription network linked to AD gene expression and triggers characteristic cellular features of the condition, including tau hyperphosphorylation, a primary driver of neurodegeneration in AD (*1*). Overall, the findings reveal that TF dimerization can contribute to the disruption of gene networks and the exacerbation of disease processes.

## RESULTS

### Aβ_42_ promotes CREB3L2-ATF4 heterodimerization

**Fig. 1.**
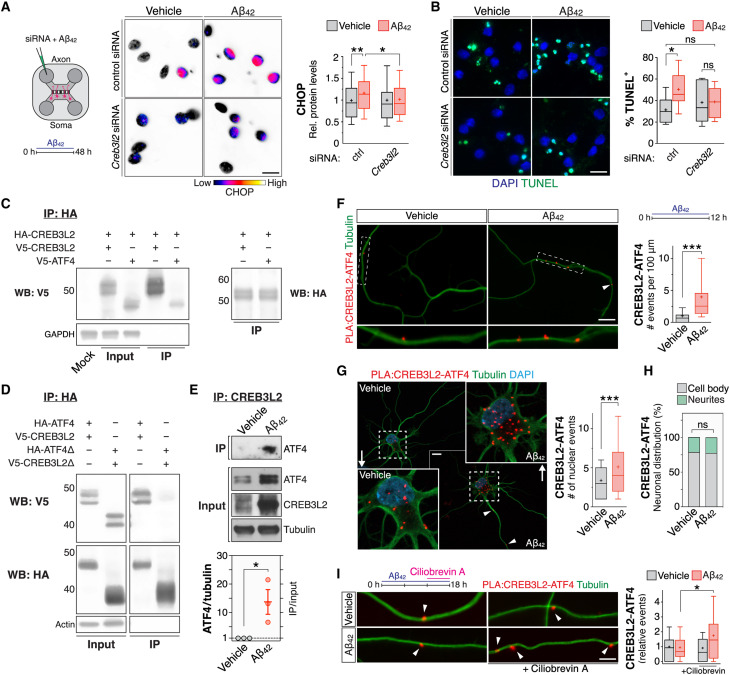
Aβ_42_ promotes CREB3L2-ATF4 heterodimerization. (**A**) Analysis of nuclear CHOP protein by quantitative immunofluorescence. Axons were transfected with control or *Creb3l2*-targeting siRNAs before Aβ_42_ treatment. Box plots summarize *n* = 8 replicates; ***P = 0.0008, one-way analysis of variance (ANOVA) with Bonferroni correction. Here and henceforth, whiskers denote 10th to 90th percentile; “+” sign marks sample mean. *Creb3l2* knockdown ranged between 77.3 and 86.5% in dissociated neurons. Scale bar, 15 μm. (**B**) TUNEL assay in hippocampal neurons. Experimental outline as in (A). Box plots summarize *n* = 10 to 12 replicates; *P = 0.0357, one-way ANOVA with Bonferroni correction. Scale bar, 15 μm. (**C**) CREB3L2-ATF4 coimmunoprecipitation using in vitro translated proteins. IP, immunoprecipitation; WB, Western blot. (**D**) CREB3L2-ATF4 coimmunoprecipitation analysis in HEK293 cells. (**E**) Coimmunoprecipitation of endogenous CREB3L2-ATF4 in neuritic extracts treated with Aβ_42_. Mixed cortical and hippocampal neurons were grown on transwell inserts. ATF4 levels are normalized against input βIII-tubulin. Plot shows mean of *n* = 3 replicates; *P = 0.0418, unpaired *t* test. (**F**) Visualization of axonal CREB3L2-ATF4 by PLA. Hippocampal neurons were cultured in microfluidic chambers, and axons were treated with Aβ_42_ for 12 hours. Box plots summarize *n* = 3 replicates; ***P < 0.0001, unpaired *t* test. Scale bar, 10 μm. (**G**) PLA visualization of CREB3L2-ATF4 in dissociated hippocampal neurons. Aβ_42_ was bath-applied for 12 hours. Box plots summarize *n* = 3 replicates. Nuclear CREB3L2-ATF4 events: ***P = 0.0007; somatic CREB3L2-ATF4 events: **P = 0.0016 (unpaired *t* tests). ns, not significant. Scale bar, 10 μm. (**H**) Subcellular distribution of CREB3L2-ATF4. This is the same dataset analyzed in (G). (**I**) PLA visualization of CREB3L2-ATF4 after inhibition of axonal retrograde transport. Ciliobrevin A was delivered in the last 6 hours of an 18-hour Aβ_42_ protocol. Box plots summarize *n* = 3 replicates; ***P < 0.0001, one-way ANOVA test with Bonferroni correction. PLA signals were normalized to axon length. Scale bar, 10 μm.

The basic region leucine zipper (bZIP) TF ATF4, an integral part of the unfolded protein response (*13*), is constitutively expressed in neurons and contributes to synaptic plasticity and memory formation (*14*–*16*). ATF4 is also associated with various neurodegenerative disorders, including AD, and is known to activate both prosurvival and prodeath signaling pathways in a context-dependent manner (*17*, *18*). We have previously reported that axonally synthesized ATF4 mediates pathogenic transcriptional changes and neurodegeneration triggered by soluble oligomeric Aβ_42_ (*19*, *20*), a neurotoxic β-amyloid peptide linked to the onset of AD (*1*). What accounts for the different functions displayed by ATF4 in these various settings? In particular, how does ATF4 function downstream of Aβ_42_ as a driver of AD pathogenesis? Because bZIP TFs operate as obligate dimers (*9*), we hypothesized that differential heterodimerization could modulate ATF4’s unique mode of action in response to Aβ_42_ by allowing for a distinct transcriptional output. To identify such potential ATF4-binding partners, we delivered small interfering RNAs (siRNAs) to axons of hippocampal neurons cultured in microfluidic chambers and screened for genes involved in the retrograde spread of β-amyloid pathology. Specifically, we focused on candidate mRNAs that, like *Atf4*, were previously found to be recruited into axons upon exposure to Aβ_42_ (fig. S1, A and B) (*19*) and evaluated the effect of their local knockdown by two criteria: prevention of C/EBP homologous protein (CHOP) induction, a prodeath effector TF downstream of ATF4-mediated neurodegeneration (*19*), and mitigation of cell death. The bZIP TF CREB3L2 passed on both counts, in that its knockdown prevented the somatic activation of *Chop* (Fig. 1A) and reduced Aβ_42_-promoted cell death (Fig. 1B), akin to the effects of ATF4 suppression (*19*). By contrast, silencing *Hif1a*, another axon-localizing bZIP TF mRNA (*19*), did not protect against Aβ_42_ (fig. S1C). As another approach to interfere with CREB3L2 generation and validate our initial screen, we locally inhibited S2P, a protease required for the transcriptional activation of this TF (*21*). Like CREB3L2 knockdown, S2P pharmacological inhibition abolished the induction of CHOP expression and ameliorated the retrograde degeneration response triggered by axonal Aβ_42_ stimuli (fig. S1, D and E). Subsequent chromatin immunoprecipitation quantitative polymerase chain reaction (ChIP-qPCR) analyses in dissociated neurons further showed that CREB3L2 directly bound a proximal promoter/enhancer region of *Chop* and that this association was potentiated by Aβ_42_ (fig. S1F).

**Fig. 2.**
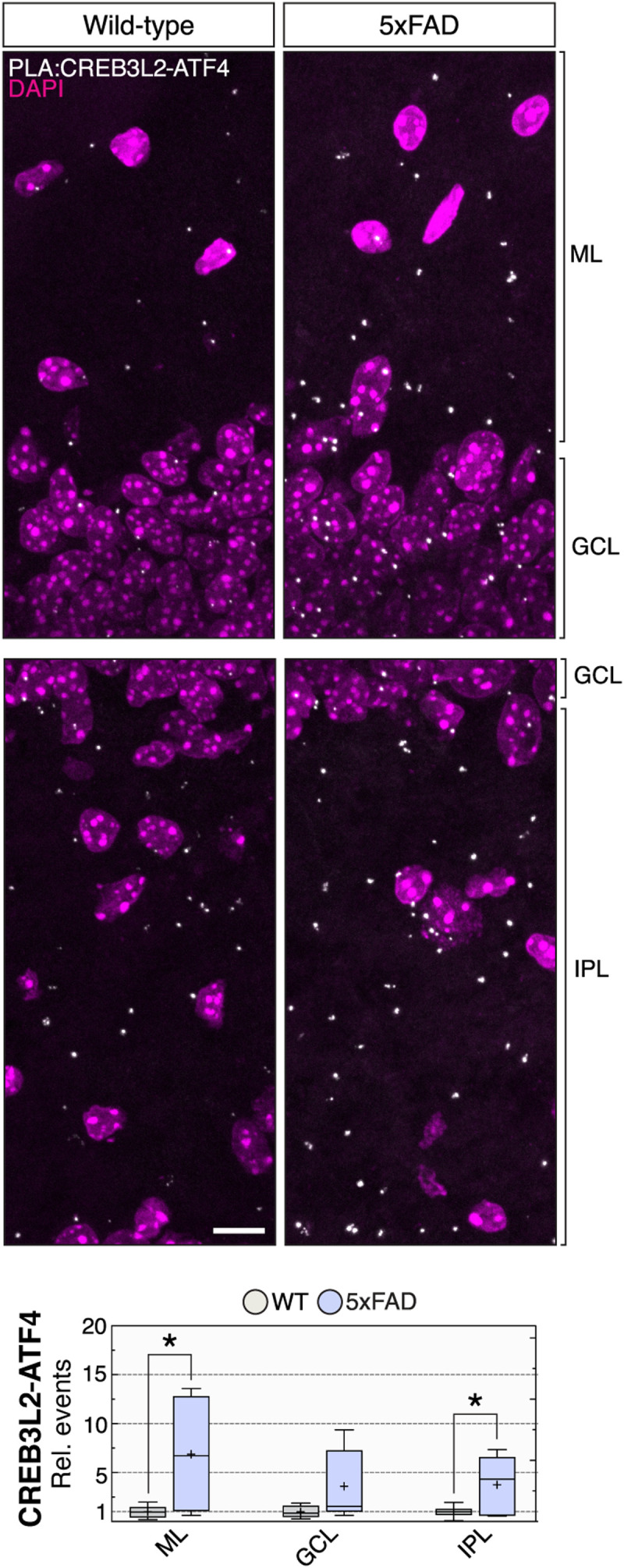
CREB3L2-ATF4 up-regulation in β-amyloid pathology 5xFAD model. In vivo detection and quantification of CREB3L2-ATF4 dimers by PLA in the dentate gyrus of 10-week-old 5xFAD or wild-type hippocampus. ML, molecular layer; IPL, inner polymorphic layer; GCL, granule cell layer. Wild-type (WT), *n* = 6; 5xFAD, *n* = 5; ML, *P = 0.0182; GCL, *P* = 0.0574; IPL, *P = 0.0285; unpaired one-tailed *t* tests. Whiskers extend to the smallest and largest data values, and sample means are indicated by + sign. Scale bar, 10 μm.

These observations raised the possibility that CREB3L2 and ATF4 act in the same Aβ_42_-initiated signaling pathway. Coimmunoprecipitation studies with either in vitro translated or overexpressed tagged proteins revealed that CREB3L2 and ATF4 form stable heterodimers via their leucine zipper domains (Fig. 1, C and D), the canonical dimerization motif of bZIP TFs. Moreover, in axon-dendritic preparations, ATF4 readily coimmunoprecipitated with CREB3L2 in response to Aβ_42_ stimulation, differently from vehicle-treated control neurites (Fig. 1E and fig. S1G). We additionally visualized their interaction by proximity ligation assay (PLA) and found that Aβ_42_ promoted increased signals for CREB3L2-ATF4 in axons and somas within 12 hours of treatment (Fig. 1, F and G, and fig. S1H). CREB3L2-ATF4 dimers were predominantly detected in the somatic compartment (~78% of all events), both inside and outside nuclei, of control and Aβ_42_-treated neurons (Fig. 1, G and H). We note, however, that axon-derived CREB3L2-ATF4 likely contributes to this somatic pool in a nonnegligible way, given that inhibition of dynein, the protein motor of axonal retrograde transport, led to a buildup of CREB3L2-ATF4 signals in axons treated with Aβ_42_ (Fig. 1I). These analyses also revealed that axonal CREB3L2-ATF4 interactions receded to control levels within 18 hours of Aβ_42_ exposure, suggesting that the surge in axonal CREB3L2-ATF4 signaling is temporally limited, primarily occurring in the first 12 hours of stimulation (Fig. 1I). In addition, we found that Aβ_42_-promoted axonal CREB3L2-ATF4 heterodimerization is dependent on local protein synthesis, as treatment with emetine, an inhibitor of ribosome activity, in the last 60 min of a 12-hour Aβ_42_ protocol limited their association in axons to control levels (fig. S1I). Quantification of CREB3L2-ATF4 signals in a mouse model of Aβ_42_ deposition (5xFAD; B6SJLF1/J background; 10-week-old animals) revealed a significant accumulation of the heterodimer in the hippocampal dentate gyrus, specifically in the molecular layer (ML) and inner polymorphic layer (IPL) in relation to age-matched controls (Fig. 2), providing in vivo evidence that CREB3L2-ATF4 heterodimerization is also increased by Aβ_42_ in mouse brain. By contrast, α-synuclein fibrils, protein aggregates tied to the progression of Parkinson’s disease (*22*), produced no increments in CREB3L2-ATF4 heterodimerization when incubated with cultured hippocampal neurons for 10 days (fig. S1, J and K), suggesting a certain level of specificity for Aβ_42_ as a modulator of this transcriptional pathway. Together, our findings identify CREB3L2 as a dimerization partner of ATF4 and show that Aβ_42_, an early trigger of AD pathogenesis (*1*), potentiates their heterodimerization.

### ChIPmera resolves genomic binding patterns of dimeric TFs

**Fig. 3.**
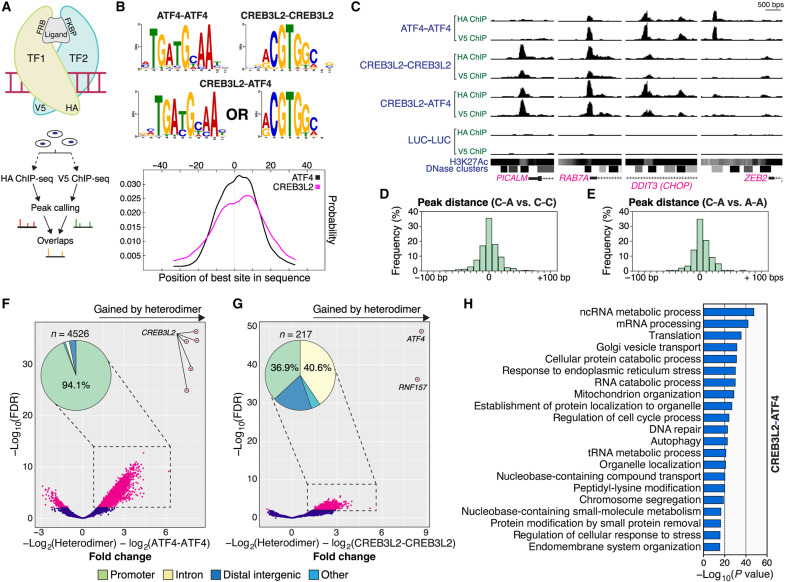
The CREB3L2-ATF4 transcriptional program. (**A**) Experimental outline. ChIPmera uses chemically induced proximity to promote the formation of specific TF pairs. Each monomer is engineered with two unique features: a specific dimerization domain (FRB or FKBP, C-terminally fused) and an N-terminal epitope tag (HA or V5). (**B**) Motif analysis of CREB3L2-CREB3L2, ATF4-ATF4, and CREB3L2-ATF4 binding sites. Heterodimer-bound sequences were centrally enriched in canonical CREB3L2 or ATF4 recognition motifs. CREB3L2: CentriMo *E* = 4.7 × 10^−39^; ATF4: CentriMo *E* = 4.0 × 10^−34^. Other motifs identified were not centrally distributed. (**C**) Representative binding behaviors are displayed by CREB3L2-CREB3L2, ATF4-ATF4, and CREB3L2-ATF4 dimers. ENCODE-generated histone H3 lysine 27 acetylation (H3K27Ac) and deoxyribonuclease I (DNase I) hypersensitivity profiles are shown. (**D**) Coincidence analysis of CREB3L2-ATF4 and CREB3L2-CREB3L2 DNA binding sites across the genome. Genomic distances were computed within a ±2-kb window around CREB3L2-CREB3L2 peaks and are plotted as a frequency histogram using a 10-bp bin size. (**E**) Same as in (D), except that here CREB3L2-ATF4 and ATF4-ATF4 dimers are compared. (**F**) Differential enrichment analysis of genomic regions bound by CREB3L2-ATF4 and ATF4-ATF4 dimers. Enrichment fold change and statistical significance are plotted along the *x* and *y* axes, respectively, from *n* = 2 independent replicates (each replicate includes two parallel ChIP-seq runs). Magenta data points: false discovery rate (FDR) ≤ 0.01; blue data points: FDR > 0.01. (**G**) Differential enrichment analysis of genomic regions bound by CREB3L2-ATF4 and CREB3L2-CREB3L2 dimers. Axes layout and data point codification are the same as in (F). The strong signal ascribed to *RNF157* coincides with an *ATF4* pseudogene found within this locus. (**H**) GO functional analysis of the CREB3L2-ATF4 transcriptional program (biological process).

While AD-associated gene expression changes have been characterized in detail (*4*–*7*), they occur via mostly unknown mechanisms. As an Aβ_42_-regulated TF heterodimer, CREB3L2-ATF4 could contribute to these transcriptional responses, prompting us to investigate its DNA binding program. However, despite recent methodological developments allowing for an unprecedented understanding of the binding specificities of TF dimers (*23*, *24*), these protocols are not tailored to their study in a cellular context and have a steep technical barrier to entry. Instead, we developed an approach that builds upon the well-established ChIP-sequencing (ChIP-seq) protocol. In our workaround, CREB3L2 and ATF4 were fused with FKBP/FRP domains, and specific homo- or heterodimers were promoted in human embryonic kidney (HEK) 293 cells using chemically induced proximity (*25*). In addition, each TF monomer was also tagged with a unique epitope [hemagglutinin (HA) or V5] to facilitate its capture and purification of bound chromatin (Fig. 3A and fig. S2, A to D). We call this system “ChIPmera,” since it is based on a molecular chimera composed of two TFs.

Preliminary reporter assays with chemically induced CREB3L2-ATF4 heterodimers showed that their pairing makes up a functional unit capable of significantly driving *Chop* activation, as interference with CREB3L2 or ATF4 using dominant-negative bZIP-like inhibitor peptides completely prevented CREB3L2-ATF4–induced reporter expression gains above baseline levels (fig. S2E) (*26*, *27*). Our evaluation of control *Renilla* luciferase homodimers, equally promoted by chemically induced proximity, further revealed that the ChIPmera protocol produces nearly no background noise (fig. S2F), averaging just 12 peaks across all replicates. In addition, maximal CREB3L2 homodimer signals were present within 15.1 base pairs (bp) of each other, a figure closely matched by coincident CREB3L2-ATF4 peaks (14.6 bp); ATF4 pairs were even more proximal, binding, on average, 11.3 bp apart (fig. S2G). These results indicate that our methodology offers very good overall DNA binding site resolution.

Unbiased motif searches using CentriMo revealed that CREB3L2-ATF4–bound DNA fragments were centrally enriched in either CREB3L2 or ATF4 canonical recognition sites (Fig. 3B and fig. S2H) (*28*). These analyses also failed to detect novel motifs associated with CREB3L2-ATF4, suggesting that the heterodimer does not determine new DNA binding specificities. In this regard, note that the CREB3L2 motif is analogous to the central tetramer of the cyclic adenosine 3′,5′-monophosphate (AMP) response element (5′-TGACGTCA-3′) bound with high affinity by various members of the CREB/ATF family, including ATF4 (*29*), which likely explains the permissive binding complementarity demonstrated by both TFs (Fig. 3B and fig. S2H). We further found that genomic regions enriched in CREB3L2-ATF4 were also targeted by CREB3L2-CREB3L2 and/or ATF4-ATF4 (Fig. 3, C to E). Still, only a fraction of genes regulated by ATF4 homodimers were bound by the heterodimer (Fig. 3, C and F), while most CREB3L2-ATF4 hits were also part of the CREB3L2 homodimer dataset (Fig. 3, C and G). Globally, the CREB3L2-ATF4 heterodimer shared far more of its transcriptional signature with CREB3L2 than ATF4 (Fig. 3, F and G), a particularly interesting observation considering that homodimerization may be the default physiological (i.e., nonpathological) configuration of CREB3L2 (*30*). Also informative was the genomic distribution of CREB3L2-ATF4: Like CREB3L2 homodimers, the heterodimer predominated in the vicinity (±3 kb) of transcription start sites, accounting for 91.4% of all signals (fig. S2I).

### Proteostasis and trafficking are targeted by CREB3L2-ATF4

Next, we performed a gene ontology (GO) enrichment analysis to explore the functional profile of CREB3L2-ATF4. Top statistically overrepresented GO terms among CREB3L2-ATF4–bound genes included RNA metabolism, protein translation and turnover, endoplasmic reticulum (ER) stress, mitochondrial organization, DNA repair, and intracellular vesicular trafficking (Fig. 3H). Overall, while the heterodimer combined biological functions individually associated with CREB3L2 and ATF4 (or both), the CREB3L2 program was the predominant factor determining its specificity. We additionally noted that a surprisingly high number of CREB3L2-ATF4 signals mapped within AD risk loci (*31*). These included in their vicinity *ABCA7*, *ADAM10*, *ADAMTS1*, *BCKDK*, *BIN1*, *CELF1*, *CSTF1*, *CD2AP*, *EED*, *FERMT2*, *HESX1*, *IQCK*, *KAT8*, *MEF2C*, *PICALM*, *PSMC3*, *OARD1*, and *ZCWPW1*, many of which directly relate to AD susceptibility through their impact on the endocytic pathway and amyloid beta percursor protein (APP) homeostasis (Fig. 3C and fig. S3A) (*32*).

Genes uniquely regulated by the CREB3L2-ATF4 heterodimer (i.e., not shared with the CREB3L2 homodimer; Fig. 3G) were associated with the unfolded protein and oxidative stress responses, the proteasome, and cell adhesion, among others (fig. S3B). Up to 91.7% of these signals coincided with ChIP-seq ATF4 peaks previously characterized by the ENCODE Consortium, indicating that they represent specific components of the ATF4 program that CREB3L2-ATF4 integrates. Their genomic distribution was also more diversified than most other CREB3L2-ATF4 signals, with only 36.9% found within ±3 kb of a transcription start site (fig. S3C). *CREB3L2* and *ATF4* were themselves strongly bound by the CREB3L2-ATF4 heterodimer (fig. S3D), suggestive of an autoregulatory mechanism.

Collectively, our analyses uncover the CREB3L2-enriched DNA binding program of the CREB3L2-ATF4 heterodimer and reveal direct links with key pathways associated with cellular proteostasis and trafficking. They also serve as proof of concept for ChIPmera in its ability to identify genomic sites bound by specific TF dimers within their cellular environment.

### CREB3L2-ATF4 orchestrates AD-linked transcription network

**Fig. 4.**
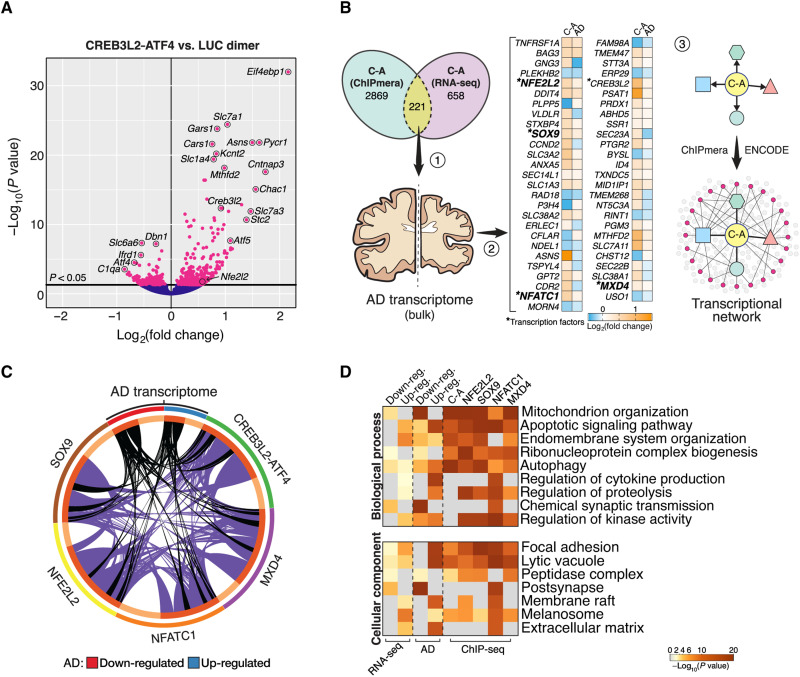
CREB3L2-ATF4 orchestrates an AD-linked transcription network. (**A**) Gene expression changes triggered by CREB3L2-ATF4 in rat hippocampal neurons analyzed by RNA sequencing (RNA-seq). Basal expression levels were measured in cells expressing *Renilla* luciferase homodimers. Fold changes over baseline (log_2_ transformed) and adjusted *P* values (−log_10_ transformed), as calculated by DESeq2 from *n* = 5 independent replicates, are plotted along the *x* and *y* axes, respectively. Magenta data points: adjusted *P* < 0.05; blue data points: adjusted *P* > 0.05. (**B**) Strategy used to characterize the AD-associated transcription network regulated by CREB3L2-ATF4. First, we determined which of the differentially expressed genes (DEGs) in our RNA-seq dataset were direct DNA binding targets of CREB3L2-ATF4 as identified by ChIPmera. Second, we evaluated the transcriptional signatures of these common hits in AD prefrontal cortex to understand which CREB3L2-ATF4–regulated targets had relevant disease-associated expression profiles (significance cutoff: *P* < 1 × 10^−15^). We found that this subset included four up-regulated TFs, *NFE2L2*, *SOX9*, *NFATC1*, and *MXD4*, as well as *CREB3L2*. Third, we explored the regulatory connections and functional relationships within this extended transcription network, described in (C) and (D). (**C**) Circos plot illustrating the regulatory relationships within the wider CREB3L2-ATF4 transcription network and their interaction with the AD transcriptome (bulk tissue level). Genes within each category (e.g., CREB3L2-ATF4 targets) are arranged along the ideogram’s arc. Inner lines link genes shared by two datasets (e.g., SOX9 and NFATC1); black-colored lines additionally identify genes with AD-associated transcriptional profiles. (**D**) Representative GO terms enriched across input gene lists, colored by *P* values (−log_10_-transformed). This comparative analysis integrates the DNA binding program of each TF, AD-associated gene expression changes, and the neuronal transcriptional profile promoted by CREB3L2-ATF4 measured in (A).

To gain further insight into the pathological role of CREB3L2-ATF4, we performed RNA-sequencing (RNA-seq) on primary rat hippocampal neurons with increased dosage of CREB3L2-ATF4 using, as above, chemically induced proximity to promote their dimerization (Fig. 4A and fig. S4, A to E). Significant hits (*P* < 0.05) were then compared against the AD transcriptome (Fig. 4B). Of 879 differentially expressed genes (DEGs) downstream of CREB3L2-ATF4 activation, 221 (approximately 25%; representation factor = 2.0, *P* < 2.2 × 10^−26^, hypergeometric test) were identified as DNA binding targets of the heterodimer in our ChIPmera study (Fig. 4B), indicating that CREB3L2-ATF4 has direct and indirect (i.e., downstream) transcriptional effects. These 221 genes were subsequently evaluated against a previously published late-onset AD transcriptome, in which the dorsolateral prefrontal cortex was profiled at bulk tissue level (*4*), revealing a subgroup of 53 genes directly regulated by CREB3L2-ATF4 with disease-associated differential expression (significance cutoff defined as *P* < 1 × 10^−15^; Fig. 4B and fig. S5A). It included the tumor necrosis factor receptor *TNFRSF1A*, which contributes to AD pathogenesis by mediating neuronal cell death (*33*), as the most significantly increased CREB3L2-ATF4 target gene (*P* = 1.26 × 10^−35^). In addition, four up-regulated TFs—*NFE2L2* (commonly known as NRF2; *P* = 9.09 × 10^−30^), *SOX9* (*P* = 2.24 × 10^−27^), *NFATC1* (*P* = 4.74 × 10^−21^), and *MXD4* (*P* = 6.16 × 10^−15^)—were part of the AD-associated transcriptional program specifically mediated by CREB3L2-ATF4 (Fig. 4B and fig. S5, A to D) (*34*). Except for *MXD4*, these TFs were not differently expressed in CREB3L2-CREB3L2 neurons (fig. S4A), constituting a unique aspect of the CREB3L2-ATF4 program. While the DNA binding programs of CREB3L2-CREB3L2 and CREB3L2-ATF4 dimers overlap to a large degree (Fig. 3G), our RNA-seq analyses showed that their transcriptional responses diverged substantially in terms of both genes and biological processes affected by their activation (fig. S4, B to D), underscoring the distinct functional identity of CREB3L2-ATF4. It is also noteworthy that *CHOP* was one of the direct targets whose expression was significantly increased by CREB3L2-ATF4 in neurons (log fold change = +0.31, *P* = 0.04), corroborating our observations in Aβ_42_ neurons (Fig. 1A and fig. S1, D and F); however, its up-regulation profile in AD did not meet our stringent significance cutoff and was not considered further in our analysis.

### CREB3L2-ATF4 transcription network is functionally tied to AD pathophysiology

The finding that CREB3L2-ATF4 controls a transcription network activated in AD speaks to a broader role for the heterodimer in modulating disease-linked gene expression, prompting us to explore its regulatory and functional relationships. To this end, we combined ChIP-seq analyses from the ENCODE Consortium (the exception being SOX9, whose transcriptional program was mined from published literature) (*35*) with the ChIPmera CREB3L2-ATF4 readout and contrasted these datasets against the AD transcriptome (top 3000 DEGs; bulk tissue level) as well as our own RNA-seq results using the Metascape platform (Fig. 4, C and D, and fig. S5E) (*36*). Doing so allowed us to identify which co-regulated gene modules within this transcriptional circuit were altered in AD. Notably, processes related to mitochondria, apoptotic signaling, the endosome, autophagy, and synaptic transmission, each with well-described links to AD pathophysiology (*37*, *38*), were enriched across the wider CREB3L2-ATF4 regulatory network (Fig. 4D). This analysis also highlighted important nuances within the network for a system-level interpretation of AD-linked gene misregulation. For example, while both mitochondrial and synaptic GO terms are down-regulated AD-related functions, the former is co-regulated by all TFs in this network, whereas the latter is exclusively related to the NFATC1 program (Fig. 4D). Proteolysis, on the other hand, is targeted by the four “downstream” TFs but not directly by CREB3L2-ATF4 (Fig. 4D), illustrating how the heterodimer can have meaningful effects beyond its core DNA binding program. Notably, when considering all the TF-target gene relationships within this network, we found that 52.2% of DEGs in AD brain (top 3000) were connected to at least one regulator (representation factor = 1.4, *P* < 2.63 × 10^−65^, hypergeometric test; Fig. 4C).

To compensate for potential biases in cell composition associated with bulk AD brain transcriptomes, we extended these analyses using neuron-specific gene expression profiles obtained from dorsolateral prefrontal cortex single-nucleus RNA-seq (snRNA-seq) datasets (fig. S6A) (*7*). We observed strong links to AD transcriptional responses in excitatory neurons, with 62.8% of down-regulated DEGs targeted by one or more regulators (representation factor = 1.7, *P* < 1.91 × 10^−37^, hypergeometric test; fig. S6B). Up-regulated DEGs also showed appreciable levels of overlap with CREB3L2-ATF4 and its wider network (43.5%; representation factor = 1.2, *P* < 0.031, hypergeometric test), but note that gene repression constitutes 74.7% of the expression signature of these cells in AD (*n* = 565 of 756; fig. S6B) (*7*). CREB3L2-ATF4 alone directly interacts with 25.1% of all significantly altered genes in AD excitatory neurons (representation factor = 2.1, *P* < 1.57 × 10^−24^, hypergeometric test). Among others, functional enrichment analyses highlighted processes related to intracellular transport in connection to the heterodimer’s regulatory activity in these cells (fig. S6, C and D), in line with our results in cultured neurons with induced CREB3L2-ATF4 dimerization (Fig. 4A and fig. S4, A to D). Differently, no enriched functional terms were uncovered in AD inhibitory neurons due to the low number of DEGs (*n* = 51) identified in this subpopulation (*7*). In any case, we found an equally high, statistically significant degree of interaction between the CREB3L2-ATF4 network and down-regulated DEGs in inhibitory cells (59.8%; representation factor = 1.6, *P* < 0.001, hypergeometric test), suggesting a role for CREB3L2-ATF4 in promoting gene repression in AD neurons. Together, these analyses provide a data-driven, unbiased view of AD-relevant cellular dysfunctions to which the CREB3L2-ATF4 heterodimer potentially contributes via its transcriptional program.

### CREB3L2-ATF4 activation recapitulates AD retromer misregulation

**Fig. 5.**
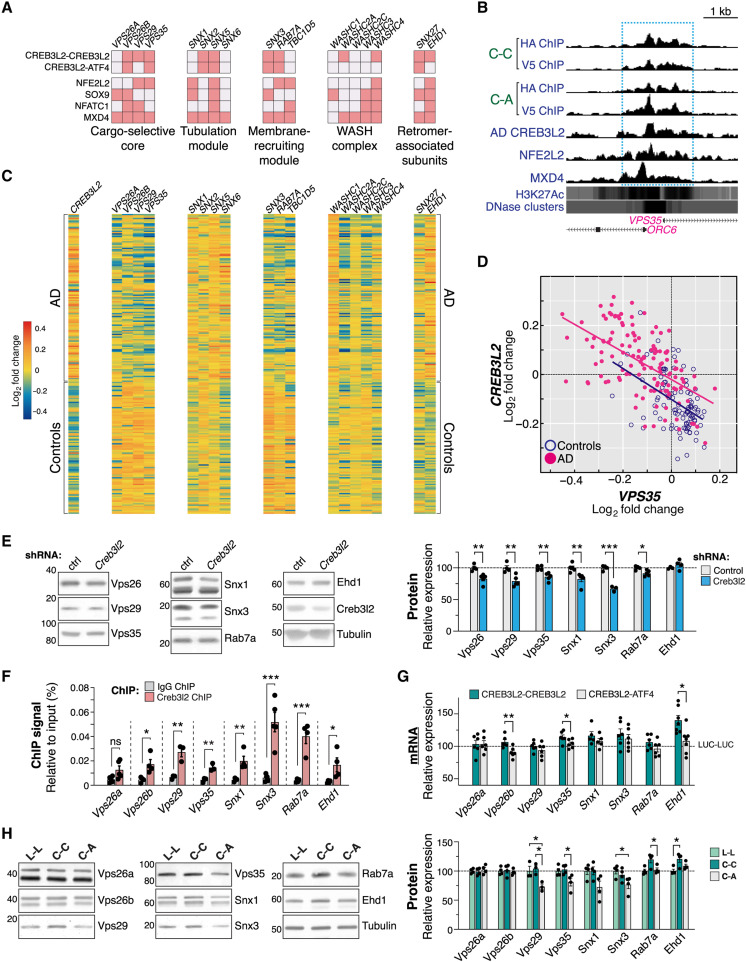
CREB3L2-ATF4 activation recapitulates transcription misregulation profile of AD retromer. (**A**) Retromer regulatory inputs within the CREB3L2-ATF4 transcription network. Pink-colored squares denote significant enrichments. (**B**) *VPS35* ChIP-seq tracks alongside ENCODE-produced H3K27Ac marks and DNase I hypersensitivity profiles. (**C**) Retromer transcriptomic profiles, mined from Zhang *et al*. (*4*), in nondemented control and AD individuals (bulk prefrontal cortex). Data are log_2_-transformed. Rows denote individual cases (controls, *n* = 101; AD, *n* = 129). *EHD1*: Pearson *r* = 0.79; *VPS29*: Pearson *r* = −0.72; *SNX1*: Pearson *r* = 0.70; *SNX6*: Pearson *r* = 0.68; *VPS35*: Pearson *r =* −0.67; *VPS26B*: Pearson *r* = −0.65. (**D**) *VPS35* (*x* axis) and *CREB3L2* (*y* axis) mRNA expression prefrontal cortex. Controls, Pearson *r* = −0.4481, ***P < 0.0001; AD, Pearson *r* = −0.5595, ***P < 0.0001. (**E**) Retromer protein levels in hippocampal neurons infected with control or *Creb3l2*-targeting shRNAs. Measurements were normalized to βIII-tubulin and are presented relative to control baseline. Mean ± SEM of *n* = 3 to 4 replicates; unpaired *t* tests. (**F**) ChIP-qPCR analysis of CREB3L2 normalized to total input chromatin. Mean ± SEM of *n* = 3 to 5 replicates; unpaired *t* tests. (**G**) RT-qPCR analysis of retromer gene expression in CREB3L2-ATF4 (C-A), CREB3L2-CREB3L2 (C-C), and control (L-L) hippocampal neurons. Measurements were normalized to *Tubb3* (βIII-tubulin) and are presented relative to control (L-L background). Plots show individual measurements and mean ± SEM of *n* = 5 to 7 replicates; one-way ANOVA with Tukey’s multiple comparison tests. (**H**) Western blot analysis of retromer protein levels in CREB3L2-ATF4 and CREB3L2-CREB3L2 neurons. Measurements were normalized to βIII-tubulin levels and are shown relative to control (L-L) baseline. Plots show individual measurements and mean ± SEM of *n* = 4 replicates (Vps29, *n* = 3); one-way ANOVA tests with Sidak’s multiple comparison correction.

Next, we sought to characterize how the heterodimer might influence specific AD gene expression responses. Among other functions, endosome-related processes were consistently enriched across our various datasets (Fig. 3H and fig. S4, C and E), including when analyzed in the context of AD-associated transcriptional changes (Fig. 4D and fig. S6, C and D). We encountered multiple direct links to the retromer, a master endosomal cargo-sorting complex whose dysfunction is implicated in the pathogenesis of AD and other neurodegenerative disorders (*39*–*41*). For example, the CREB3L2-ATF4 program includes various subunits of the retromer cargo-selective, tubulation, and membrane-recruiting modules, which CREB3L2-CREB3L2 mostly shares (Fig. 5, A and B). The retromer machinery additionally receives extensive inputs from the wider NRF2-SOX9-NFATC1-MXD4 network (Fig. 5, A and B), further hinting at a potentially important role for this pathway in mediating retromer gene expression mechanisms. Retromer dysfunction in AD results, at least partly, from the deficient expression of two core subunits, VPS26 and VPS35, in the brain of affected individuals (*41*). Still, while the proamyloidogenic effects of a malfunctioning retromer are well characterized (*40*–*43*), it remains unresolved why retromer transcriptional misregulation occurs in AD in the first place. This gap prompted us to examine more closely a potential connection between CREB3L2-ATF4 and retromer-mediated endosomal dysfunction.

First, by inspecting AD-associated retromer gene expression patterns in the dorsolateral prefrontal cortex (*4*), we discovered that retromer misregulation was more extensive than previously recognized (Fig. 5, C and D), i.e., not restricted to VPS26 and VPS35 but evident across its different modules. These changes were particularly pervasive among subunits of the cargo-selective and membrane-recruiting modules, which were down-regulated in the diseased brain, and had *VPS29* ranking as the most significantly altered retromer gene (log fold change = −0.14, *P* = 3.09 × 10^−24^). In addition, perturbed expression of brain-enriched *VPS26B* rather than the more ubiquitous *VPS26A* paralog was observed (Fig. 5C) (*44*). By contrast, sorting nexins *SNX1* and *SNX6*, as well as *EHD1*, implicated in endosome membrane tubulation, were up-regulated in AD (Fig. 5C). Notably, the transcriptional changes of several retromer subunits correlated strongly with *CREB3L2* expression (Pearson *r* ≥ 0.65), both positively and negatively (Fig. 5, C and D). These included *VPS26B*, *VPS29*, and *VPS35* (negative coexpression association); core retromer components involved in the selection of cargoes (*39*); as well as *EHD1*, *SNX1*, and *SNX6* (positive association). Other brain regions affected by AD develop comparable retromer misregulation profiles (figs. S7 and S8A) (*45*, *46*), indicating that these effects are not unique to the prefrontal cortex. snRNA-seq datasets similarly ranked *VPS29* as the most robust retromer DEG in AD excitatory neurons, followed, to a lesser extent, by *SNX3*, *RAB7A*, and *VPS35* (fig. S8B) (*7*). Together, our analyses reveal that the retromer machinery in AD is affected by widespread transcriptional alterations and suggest links between CREB3L2 and the breakdown of retromer regulatory mechanisms.

In line with this idea, CREB3L2 knockdown reduced the expression of various retromer subunits at both mRNA and protein levels in neurons (Fig. 5E and fig. S9A). We further confirmed by ChIP-qPCR that CREB3L2 binds to DNA regulatory elements in the vicinity of several retromer genes in these cells (Fig. 5F), consistent with CREB3L2 functioning as a constitutive transcriptional activator for the neuronal retromer. Earlier, we had noted in our RNA-seq datasets that CREB3L2-ATF4 heterodimerization dysregulated various trafficking processes, including endosome-to-Golgi retrograde transport, an export pathway mediated by the retromer machinery (fig. S4C) (*39*). This observation suggested that CREB3L2’s normal function might be impaired by its association with ATF4, potentially leading to a breakdown of retromer regulation. To test this, we measured retromer transcript and protein profiles in neurons using reverse transcription qPCR (RT-qPCR) and Western blot following CREB3L2-ATF4 induction. These studies revealed a general trend toward the down-regulation of the retromer machinery due to CREB3L2-ATF4 activation (mRNA average change = −12.9% and protein average change = −18.6%; Fig. 5, G and H). One prominent example was *Vps35*, retromer’s “backbone” subunit, which developed significantly reduced mRNA and protein levels (Fig. 5, G and H). CREB3L2-ATF4 heterodimers likewise led to the down-regulation of *Vps26b* mRNA (Fig. 5G), while the effects on *Vps29*, *Rab7a*, and *Snx3* expression were particularly evident at the protein level (Fig. 5H), suggesting that posttranscriptional mechanisms may also be at play. We detected a marked increase in *Ehd1* expression levels only in CREB3L2-CREB3L2 neurons (Fig. 5, G and H), in agreement with the finding that CREB3L2 binds to this gene exclusively as a homodimer (Fig. 5A). Similarly, *Vps26a*, which is not a transcriptional target of either CREB3L2-CREB3L2 or CREB3L2-ATF4 (Fig. 5A), was unaffected in either background (Fig. 5, G and H), showing that the disruption of retromer regulatory processes by CREB3L2-ATF4 does not occur indiscriminately. CREB3L2-ATF4 activation was also seemingly detrimental to normal retromer function in neurons, as indicated by the increased degradation of cation-independent mannose 6-phosphate receptor (CI-M6PR; fig. S9B), the canonical target of retromer-mediated endosome-to-Golgi retrieval (*47*, *48*).

Collectively, our findings reveal that the neuronal retromer is affected by CREB3L2-ATF4 via a transcriptional response in many respects comparable to that seen in AD, suggesting a mechanism for its functional impairment. They additionally provide a concrete example of how the heterodimer can produce gene expression disruptions linked to relevant AD cellular dysfunctions.

### CREB3L2-ATF4 interacts with β-amyloid and tau neuropathologies

**Fig. 6.**
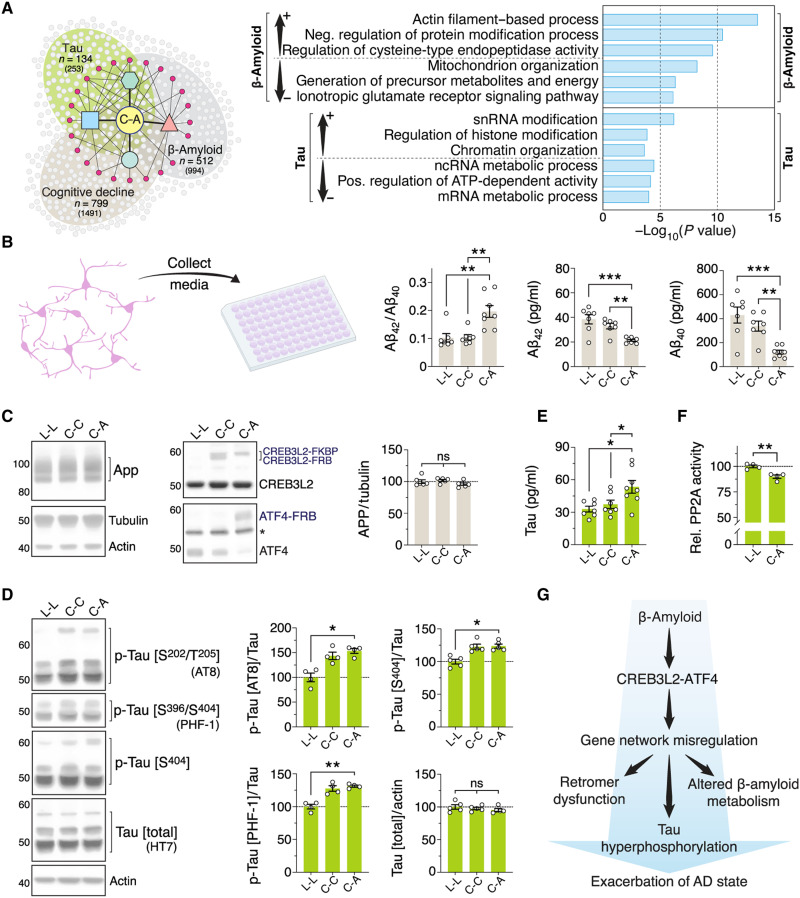
CREB3L2-ATF4 interacts with β-amyloid and tau neuropathologies. (**A**) Top three enriched GO terms for CREB3L2-ATF4–interacting genes positively and negatively associated with β-amyloid or tau neuropathologies (−3 ≤ signed −log_10_ *P* value ≥ 3). β-Amyloid pathology overlap: representation factor = 1.4, *P* < 7.54 × 10^−23^, hypergeometric test; tau pathology overlap: representation factor = 1.4, *P* < 8.15 × 10^−8^, hypergeometric test; cognitive decline overlap: representation factor = 1.5, *P* < 3.36 × 10^−43^, hypergeometric test. (**B**) Analysis of extracellular Aβ_42_, Aβ_40_, and Aβ_42_/Aβ_40_ ratios in culture supernatants from hippocampal neurons expressing CREB3L2-ATF4 (C-A), CREB3L2-CREB3L2 (C-C), or *Renilla* luciferase (L-L) dimers. Plots show individual measurements and mean ± SEM of *n* = 7 replicates; one-way ANOVA tests with Tukey’s correction. (**C**) Western blot analysis of full-length APP. Plots display individual measurements and mean ± SEM of *n* = 6 replicates. (**D**) Western blot quantification of tau phosphorylation in rat hippocampal neurons normalized against total tau levels. Plots show individual measurements and mean ± SEM of *n* = 4 replicates; one-way ANOVA tests with Tukey’s correction. Measurements shown here pertain only to tau signals around 50 kDa (see also fig. S10F). (**E**) Extracellular tau protein levels. Plots show individual measurements and mean ± SEM of *n* = 7 replicates; one-way ANOVA tests with Tukey’s correction. (**F**) Analysis of PP2A phosphatase activity in purified neuronal extracts. Plot shows mean ± SEM of relative cell number–normalized absorbance measurements from *n* = 4 replicates; **P = 0.0031, unpaired *t* test. (**G**) The findings support a model whereby CREB3L2-ATF4 promotes AD-linked gene expression changes and contributes to characteristic features of AD pathophysiology, including retromer dysfunction, altered β-amyloid metabolism, and tau hyperphosphorylation.

Because CREB3L2-ATF4 is regulated by β-amyloid, an upstream component of the Alzheimer’s pathological cascade (*1*), we next determined how the heterodimer might globally relate to this neuropathology and other characteristic disease phenotypes. To this end, we leveraged gene-trait molecular networks previously elucidated by Mostafavi and colleagues (*5*) linking AD-associated transcriptomic patterns to disease-relevant end points (e.g., β-amyloid burden or cognitive decline) and assessed their interaction with CREB3L2-ATF4. These data were originally derived from participants enrolled in the Religious Orders Study (ROS) or the Rush Memory and Aging Project (MAP), two large-scale longitudinal cohort studies of aging and dementia (*49*). We first examined gene expression changes in the dorsolateral prefrontal cortex conditioned by β-amyloid and tau neuropathologies and looked for direct overlaps with the CREB3L2-ATF4 transcription network. We subsequently used GO annotations to determine which biological processes within the CREB3L2-ATF4 network were most significantly enriched in connection to these traits (Fig. 6A and fig. S10, A to C). Doing so revealed that mitochondria and energy-related functions were negatively associated with β-amyloid burden (Fig. 6A and fig. S10A) and that gene expression modulators, such as histone modification and chromatin organization, showed the strongest positive associations with tau pathology (Fig. 6A and fig. S10B), overall indicating that CREB3L2-ATF4 heterodimerization may, to some degree, influence these well-known pathophysiological relationships (*50*–*52*). CREB3L2-ATF4–regulated gene modules associated with cognitive decline showed excellent agreement with the β-amyloid cohort, similarly highlighting mitochondria-related functions among the strongest associations (fig. S10, A and C). Cellular trafficking terms were also significantly enriched in both datasets (fig. S10, A and C), consistent with these pathways encompassing a key aspect of how CREB3L2-ATF4 contributes to AD pathophysiology.

Following up on these observations, we determined whether CREB3L2-ATF4 interacted with APP and tau regulation. We first measured soluble Aβ peptides in the medium of rat hippocampal neurons expressing CREB3L2-ATF4 heterodimers using a Meso Scale multiplex immunoassay. Compared to controls, CREB3L2-ATF4 neurons had significantly higher Aβ_42_/Aβ_40_ ratios (Fig. 6B), indicative of a shift in APP processing. This was accompanied by reduced Aβ_42_ and Aβ_40_ levels (Fig. 6B), with the decline of Aβ_40_ being, however, more pronounced than that seen for the aggregation-prone Aβ_42_ peptide (−71.9 versus −44.5%, respectively). These changes were not a result of a direct transcriptional effect of CREB3L2-ATF4 activation on *App*, as neither its mRNA nor protein expression was affected by the heterodimer (Fig. 6C and fig. S10D). We also observed a drop in sAPPα levels (−19.3%; fig. S10E), indicating that both the amyloidogenic and non-amyloidogenic APP processing pathways are affected by the heterodimer, albeit at different degrees.

To assess tau metabolism, we initially focused on neuronal tau phosphorylation patterns, as hyperphosphorylated forms of this protein are associated with increased tau aggregation in AD (*53*). Using antibodies that recognize specific tau phospho-epitopes, we found that the CREB3L2-ATF4 heterodimer led to significantly higher phosphorylation of various disease-relevant sites (Fig. 6D and fig. S10, F and G). At Ser^202^/Thr^205^, analyzed with the same AT8 antibody used in Braak staging (*54*, *55*), a ~53.4% increase over control neurons was observed by Western blot (Fig. 6D). Similarly, phosphorylation levels at Ser^396^/Ser^404^, which comprise one of the earliest AD-related abnormal tau processing events (*56*), were 31.1% higher in CREB3L2-ATF4 neurons as detected with the PHF-1 antibody (Fig. 6D) (*57*, *58*). Comparable measurements (+23.7%) were obtained with a second antibody against phosphorylated Ser^404^ (T7444), confirming that this residue is affected by the heterodimer (Fig. 6D). We additionally encountered an upward (but statistically not significant) trend in tau phosphorylation levels with CREB3L2 homodimers (Fig. 6D and fig. S10F), suggesting that CREB3L2 up-regulation alone, as seen in the aftermath of CREB3L2-ATF4 activation (Figs. 4A and 6C and fig. S3D), may also, to some degree, modulate tau dyshomeostasis.

While tau is predominantly an intracellular protein, it is also known to be released by neurons and contribute to the spread of pathology (*53*), prompting us to evaluate whether CREB3L2-ATF4 might similarly influence tau secretion. Using a Meso Scale platform, we found that extracellular tau accumulation in CREB3L2-ATF4 cultures was 62.9% above control levels (Fig. 6E). By contrast, this measure was unchanged in CREB3L2-CREB3L2 neurons (Fig. 6E). Further analyses using enzyme-linked immunosorbent assays (ELISAs) failed to detect Neurofilament-light (a biomarker of neurodegeneration) and Map2 (another member of the microtubule-associated protein family) in these samples across all conditions (fig. S10H), indicating that tau secretion is actively promoted in CREB3L2-ATF4 neurons as opposed to its extracellular accumulation being a consequence of neuronal death.

How does CREB3L2-ATF4 activation influence tau metabolism? Analysis of neuronal RNA-seq datasets revealed that various subunits of the holoenzyme protein phosphatase 2A (PP2A) were disrupted at the transcriptional level by CREB3L2-ATF4 (fig. S10I) (*53*). PP2A accounts for ~70% of tau-directed phosphatase activity in the human brain, and expression changes are proposed as a reason for its impairment in AD (*53*). For example, we found that *Ppp2ca* mRNA, which encodes a catalytic subunit of PP2A down-regulated in AD (log fold change = −0.16, *P* = 2.49 × 10^−25^) (*4*), was likewise reduced by CREB3L2-ATF4 activation in neurons (log fold change = −0.15, *P* = 0.016; fig. S10I), raising the possibility that PP2A function is negatively affected by CREB3L2-ATF4. PP2A phosphatase activity in purified extracts of CREB3L2-ATF4 neurons was significantly reduced in relation to controls (−10.1%; Fig. 6F), mechanistically in line with the altered tau phosphorylation patterns observed in these cells (Fig. 6D and fig. S10F). However, globally, a STRING enrichment analysis using gene expression changes mediated by the heterodimer as input identified 17 additional DEGs with links to tau (*P* = 1.14 × 10^−7^), including Clusterin, an important risk gene for late-onset AD implicated in tau aggregate seeding (fig. S10J) (*31*, *59*). We cannot exclude, therefore, that other pathways contribute to tau dyshomeostasis downstream of CREB3L2-ATF4. Together, our findings reveal that CREB3L2-ATF4 drives abnormal tau phosphorylation and secretion in neurons, two key aspects linked to the development and spread of tau pathology, overall supporting a model whereby the heterodimer is regulated by and functionally interacts with AD neuropathologies (Fig. 6G).

### CREB3L2-ATF4 heterodimers are present in AD brain

**Fig. 7.**
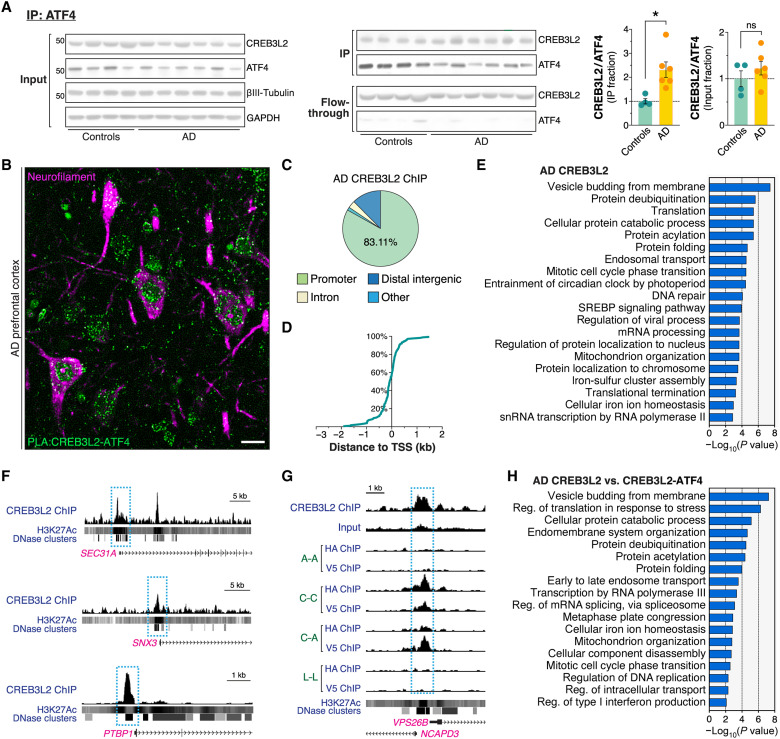
CREB3L2-ATF4 in the human AD brain. (**A**) Coimmunoprecipitation analysis of CREB3L2-ATF4 heterodimers in control and late-onset AD prefrontal cortex (immunoprecipitation with anti-ATF4 antibody). Plots show individual measurements and mean ± SEM of CREB3L2/ATF4 ratios from *n* = 4 controls and *n* = 6 AD cases; *P = 0.0133, unpaired *t* test. (**B**) PLA detection of CREB3L2-ATF4 heterodimers (green punctate signals) in AD dorsolateral prefrontal cortex costained for neurofilament (magenta labeling), a neuronal marker. This pseudocolored representative micrograph was produced using chromogenic detection methods. See fig. S11D for quantification and technical controls. Scale bar, 25 μm. (**C**) Genomic distribution of AD CREB3L2 ChIP-seq signals. Cutoff for proximal promoter/enhancer regions was defined as ±3 kb from a transcription start site. (**D**) Cumulative frequency distribution of CREB3L2 ChIP-seq peaks relative to known transcription start sites (TSS). (**E**) GO functional analysis of AD CREB3L2 transcriptional program (biological process). (**F**) Representative CREB3L2 ChIP-seq tracks juxtaposed with ENCODE-produced H3K27Ac and DNase I hypersensitivity profiles. *SEC31A* encodes a component of the COPII protein complex and participates in vesicle budding from the ER; *SNX3* governs the interaction between the retromer and early endosomes; *PTBP1* is a splicing regulator. (**G**) AD CREB3L2 ChIP-seq genome browser tracks in *VPS26B* locus juxtaposed with ChIPmera datasets and ENCODE-produced H3K27Ac and DNase I hypersensitivity profiles. For clarity, AD ChIP-seq and ChIPmera tracks are displayed using different viewing ranges, as the ChIPmera signals are consistently stronger. (**H**) GO term enrichment analysis (biological process) of targets common to both CREB3L2-ATF4 ChIPmera and AD CREB3L2 ChIP-seq datasets.

The analyses so far offer evidence of a potentially important role for CREB3L2-ATF4 in AD pathophysiology, which motivated us to pursue additional corroboration that their heterodimerization mediates a disease-relevant transcriptional mechanism. We first determined whether CREB3L2-ATF4 was found in the human brain and to what extent its heterodimerization levels were different in AD by performing coimmunoprecipitation in samples of nondemented control and disease cases (table S1). Akin to our earlier bioinformatic analyses, we chose to evaluate tissue originating from the dorsolateral prefrontal cortex (Brodmann area 9), a cerebral region linked to higher cognitive functions affected by AD (*7*). These studies revealed that proportionally higher levels of CREB3L2 were present in ATF4 coimmunoprecipitates from AD brains than those found in controls (131% average enrichment, *P* = 0.013; Fig. 7A), particularly in individuals with advanced tau pathology (Braak stage ≥ V; fig. S11A). This was accompanied by only marginal increases in CREB3L2/ATF4 ratios in AD input fractions (23%; *P* = 0.34; Fig. 7A and fig. S11B), denoting a specific and robust enrichment of CREB3L2 in ATF4 coimmunoprecipitates. The ATF4 immunoprecipitation reaction was highly efficient, as judged by the near absence of this protein in the flow-through fraction and the complete lack of any ATF4 accumulation in control immunoglobulin G (IgG) reactions (Fig. 7A and fig. S11C). These findings suggest that an increased share of ATF4 is “occupied” by CREB3L2 in AD brains, indicating that an overall shift in ATF4 and CREB3L2 dimerization patterns occurs in association with this neurodegenerative condition.

Using PLA analyses in AD prefrontal cortex, we further found that CREB3L2-ATF4 heterodimers were present not only in neurons, predominantly inside the nucleus, but also in axons, as well as in other cells (Fig. 7B). In addition, comparisons against control cases showed that nuclear CREB3L2-ATF4 signals were enriched in AD neurons by approximately 20% (fig. S11D and table S2), an effect size that, while statistically significant, is likely attenuated by local differences in brain β-amyloid accumulation, which we did not account for in this assessment. These data reveal that neurons are a source of CREB3L2-ATF4 heterodimers in AD brain.

### CREB3L2 transcriptionally overlaps with CREB3L2-ATF4 in AD

Next, we sought to substantiate our characterization of the CREB3L2-ATF4 transcriptional program with direct human evidence. Two individuals (both females with moderate AD pathology, aged >89 years; table S3) were chosen for ChIP-seq analysis based on high prefrontal cortex CREB3L2 and ATF4 expression in addition to reduced postmortem processing intervals. To our knowledge, ChIP-seq of point-source TFs in human brains has not been reported, likely due to the inherent technical challenges associated with biobanked material. While our efforts to immunoprecipitate ATF4-bound chromatin were unfruitful, we resolved 228 genomic sites enriched in CREB3L2, assignable to a set of 179 functionally coherent protein-coding genes (STRING network enrichment: *P* = 1.22 × 10^−7^).

In agreement with our ChIPmera study, further breakdown of CREB3L2 binding showed that 83.1% of these signals were located within ±3 kb of a transcription start site (Fig. 7, C and D), validating our earlier observation that CREB3L2 preferentially engages with proximal promoter/enhancer regions (Fig. 3F). Likewise, GO terms related to intracellular trafficking, including endosomal transport, as well as ER stress, proteostasis, RNA metabolism, mitochondrial organization, and DNA repair, were associated with CREB3L2 in AD (Fig. 7E and fig. S11E). Notably, the retromer complex also came up as an enriched function (Fig. 7, F and G, and fig. S11, E and F), confirming that this endocytic sorting pathway is a relevant regulatory target of CREB3L2 in AD brain. In addition, approximately half of the sites bound by CREB3L2 coincided with CREB3L2-ATF4–enriched regions (representation factor = 4.1, *P* < 1.1 × 10^−34^, hypergeometric test), and up to 78.8% were part of the CREB3L2 homodimer repertoire (representation factor = 3.5, *P* < 1.1 × 10^−58^, hypergeometric test). Functional annotation of this overlapping gene subset revealed that trafficking categories, such as endosomal transport, as well as ER stress, mitochondrial organization, and proteostasis, were common to both CREB3L2-ATF4 and AD CREB3L2 datasets (Fig. 7H and fig. S11G).

ChIPmera and the bioinformatic analyses that followed have thus captured a core set of disease-relevant pathways strikingly congruous with those regulated by CREB3L2 in AD brain. Although to some extent correlative, the findings support the pathophysiological significance of our proposed model regarding the nature of the CREB3L2-ATF4 program, especially since it is CREB3L2 that largely defines the regulatory landscape of the heterodimer (Fig. 3, F and G).

### Gene expression as a driver and intervention target in Aβ_42_ neurodegeneration

**Fig. 8.**
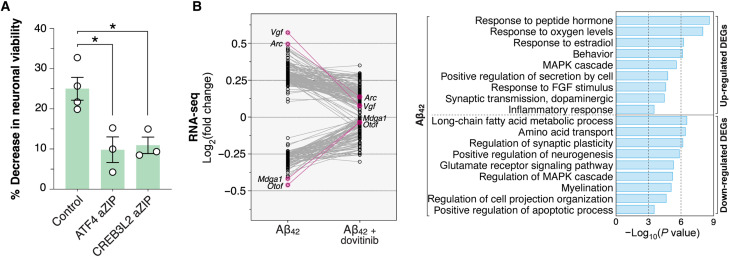
Gene expression as a driver and intervention target in Aβ_42_ neurodegeneration. (**A**) Cell viability analysis of dissociated rat hippocampal neurons after 48-hour Aβ_42_ stimulation protocol. Plots show individual measurements and mean ± SEM of *n* = 3 biological replicates. For each background, data are presented as Aβ_42_/vehicle ratios. ATF4 aZIP: *P = 0.0167; CREB3L2 aZIP: *P = 0.0137; unpaired *t* tests. (**B**) Gene expression changes triggered by Aβ_42_ in rat hippocampal neurons analyzed by RNA-seq. Basal expression levels were measured in vehicle-treated cells. DEGs identified in Aβ_42_ condition are compared against their expression values in neurons cotreated with dovitinib. Individual data points show fold changes over baseline (log_2_-transformed) of *n* = 2 biological replicates. Aβ_42_ and dovitinib were applied for 24 hours before RNA collection. A GO enrichment analysis of DEGs (*n* = 203; 128 up-regulated, 75 down-regulated) in Aβ_42_-treated neurons is also shown. FGF, fibroblast growth factor; MAPK, mitogen-activated protein kinase.

Because CREB3L2-ATF4 interacts with a substantial subset of the AD transcriptome and recapitulates aspects of disease progression, disrupting its activity may mitigate the detrimental effects mediated by Aβ_42_ and hence potentially improve disease outcomes. Targeted interference with bZIP TFs is well established and has found applicability in cancer models (*26*, *27*). Briefly, bZIPs homo- and/or heterodimerize by forming a parallel coiled-coil (the “leucine zipper”) and bind DNA via a proximal region rich in basic (i.e., positively charged) amino acids (*9*, *10*, *30*). Replacing the latter with an acidic (i.e., negatively charged) sequence creates very efficient dominant-negative bZIP sponges (aZIPs), which simultaneously prevent dimerization and DNA binding of target TFs (*26*, *27*). On the basis of this principle, we designed CREB3L2 and ATF4 aZIPs and tested their ability to rescue Aβ_42_-induced neuronal cell death after viral delivery. Both peptides significantly improved cell viability in neurons exposed to Aβ_42_ across a 48-hour stimulation protocol in relation to wild-type neurons (Fig. 8A). These findings suggest that CREB3L2 and ATF4 are central effectors of Aβ_42_ neurodegeneration.

While TFs have traditionally been considered poor therapeutic targets (*60*), gene expression is emerging as a powerful platform for drug discovery and repurposing efforts (*61*, *62*). This is made possible by resources like The Connectivity Map (CMap), which contains more than 1 million gene expression signatures for a range of drugs and other perturbations (*63*). With CMap, changes in gene expression linked to a disease process can be compared for similarity to drug-induced perturbations and those with the most negative correlations followed up as therapeutic leads. A query of the CMap database using the CREB3L2-ATF4 transcriptome that included both up-regulated and down-regulated genes (top 150 DEGs, 75 from each arm) identified dovitinib, a pan-receptor tyrosine kinase inhibitor (*64*), as the most significant hit (connectivity score = −0.65; −log_10_[false discovery rate] = 15.65). This molecule had been previously classified as a top repurposing candidate for AD based on two independent analyses (*65*, *66*), prompting us to test it in neurons challenged with Aβ_42_. Using RNA-seq, we found 203 DEGs (*P* < 0.05) after a 24-hour Aβ_42_ stimulation protocol (average absolute log fold change = 0.29), 131 of which showed corrective shifts ≥0.10 with dovitinib cotreatment, and a subset of 30 genes surpassing ≥0.25 differences in head-to-head comparisons (Fig. 8B). *Vgf* was the most significantly altered gene by Aβ_42_ (log fold change [Aβ_42_] = 0.57), also undergoing the largest overall correction toward control baseline levels with dovitinib (log fold change [Aβ_42_ + dovitinib] = 0.07) (Fig. 8B). The results indicate that dovitinib can mitigate, at least to some extent, the early-phase transcriptional response downstream of Aβ_42_. Encouragingly, this drug can cross the blood-brain barrier and has a well-characterized safety profile (*67*), in addition to being nontoxic to neurons (*65*), which bodes well for future preclinical work.

Together, in this study, we have discovered a TF heterodimer regulated by β-amyloid that interacts with a molecular network linked to disease phenotypes and have confirmed that key components of this transcriptional pathway are present in the AD brain. We additionally provide evidence that gene expression may be a promising intervention target for AD therapies.

## DISCUSSION

Our findings collectively support the conclusion that CREB3L2 and ATF4 form a pathologically important association in AD and highlight TF combinatorial relationships as a relevant disease mechanism. Because TF interactions are widespread phenomena (*9*, *10*), consequential pathophysiological insights are likely within reach of future investigations. ChIPmera, a methodology that we developed to interrogate dimeric TFs, could prove particularly useful in this regard, especially when coupled with much-needed improved strategies for detecting context-dependent TF associations. CREB3L2 and ATF4 have no known genetic variants associated with AD risk and were likely to have been disregarded as candidates of study had we focused uniquely on their expression profiles, given that they would not be considered obvious top picks by this measure alone (*4*, *31*). Instead, it is their heterodimerization that makes them remarkable in the context of AD.

Despite not encompassing the full complexity of AD, the amyloid cascade hypothesis remains the predominant model of pathogenesis (*1*, *68*). It postulates that β-amyloid deposition is a key instigator of the ensuing degenerative process involving tau aggregation, neuron loss, and cognitive impairment (*68*). Elucidating the mechanisms by which β-amyloid precipitates this chain of events is of critical significance (*69*), not least because current evidence strongly suggests that tau pathology is the primary mediator of neurodegeneration in AD (*1*). CREB3L2-ATF4 bridges both hallmark AD neuropathologies, being regulated by β-amyloid and promoting aspects of tau dyshomeostasis typical of AD. Our findings thus indicate that gene expression changes are not merely responsive to but function as actual drivers of AD pathology. Given the scope of the heterodimer’s transcriptional program, it is doubtful that its effects can be ascribed to a single dysfunctional pathway (e.g., retromer or PP2A) but more likely emerge from globally interdependent disturbances spanning different cellular processes. This is ultimately why AD and other conditions with multifactorial etiologies may be ideally suited for therapeutic interventions focusing on gene expression (*65*), as these open the possibility of correcting cellular imbalances even when the underlying pathogenic mechanisms are not entirely understood.

We recognize that some inferences that we make in this study regarding CREB3L2-ATF4 are based on correlation analyses. We have accommodated this limitation by using human datasets from different sources and favoring unbiased readouts whenever possible. The identification of dovitinib attests to the strength of the approach. We and others have closed in on the same molecule using different strategies and datasets (*65*, *66*), which provides a strong indication that CREB3L2-ATF4 captures a core gene expression signature of AD. Focused development of a small molecule that specifically inhibits their heterodimerization will be needed to allow more direct validations and fundamentally prove the role of CREB3L2-ATF4.

In conclusion, we report that TF heterodimerization can encode pathogenic stimuli and reconfigure transcription networks associated with disease processes. Our study provides a novel mechanistic perspective for understanding gene expression programs in the context of AD and suggests a transcriptional link between β-amyloid and tau pathologies, the two hallmark brain lesions that characterize this neurodegenerative condition.

## MATERIALS AND METHODS

### Neuronal culture

Hippocampi were dissected from embryonic day (E) 16 to E18 rat embryos obtained from pregnant Sprague-Dawley dams (Envigo). All animal procedures were approved by the Institutional Animal Care and Use Committee (IACUC) at Columbia University. Cell dissociation was performed using TripLE Express. Neurons (50,000 to 60,000 per microfluidic chamber) were plated on poly-d-lysine (0.1 mg ml^−1^; MilliporeSigma)– and laminin (2 μg ml^−1^; Bio-Techne)–coated substrates and grown in Neurobasal supplemented with 10% fetal bovine serum, 2 mM l-glutamine, 1 mM sodium pyruvate, and antibiotics (50 U ml^−1^ penicillin-streptomycin). Tripartite microfluidic chambers were produced with Dow Corning Sylgard 184 Silicone Encapsulant Clear Kit (10:1 mix ratio; Ellsworth Adhesives) cured at ~70°C for at least 4 hours following published protocols (*19*). Chamber design incorporated two sets of 200-μm-long microgroove barriers to exclude the crossing of cell bodies and dendrites. After 24 hours, the medium was changed to Neurobasal containing 1× B27 and 2 mM l-glutamine. Subsequent medium changes (half volumes) were performed at day in vitro 4 (DIV4) and thereafter every 3 to 5 days. To prevent glial cell proliferation, medium changes included 5-flurodeoxyuridine and uridine (final concentration: 10 mM; MilliporeSigma) after DIV4. Neuronal cultures were grown in a 37°C, 5% CO_2_ humidified atmosphere until DIV12 to DIV14. Whenever stated, axonal or cell body compartments were treated with 30 μM ciliobrevin A (R&D Systems), 100 nM emetine (MilliporeSigma), or 10/15 μM nelfinavir (MilliporeSigma). Dovitinib (Selleck Chemicals) was bath-applied at 40 nM 30 min before Aβ_42_ stimulation. Unless otherwise specified, reagents were purchased from Thermo Fisher Scientific.

### Aβ_42_ peptide oligomerization and treatment

Lyophilized synthetic Aβ_42_ peptides (Bachem, H-1368) were dissolved to 1 mM in ice-cold 100% 1,1,1,3,3,3-hexafluoro-2-propanol (MilliporeSigma) by multiple rounds of pipetting, aliquoted, and spun in SpeedVac (Savant Instruments) for 30 min. The resulting peptide films were then resuspended in dimethyl sulfoxide (DMSO; MilliporeSigma) to 5 mM, further diluted to 100 μM with Ham’s F12 medium (Thermo Fisher Scientific), and incubated overnight at 4°C. Immediately before use, peptide concentration was adjusted with Neurobasal medium to a suitable working dilution and added to dissociated, noncompartmentalized cultures at concentrations of 250 to 750 nM to accommodate batch-to-batch variations. In compartmentalized neurons, oligomerized Aβ_42_ peptides were delivered at 3 μM, as previously described (*19*); this dosage reflects the ability of the polymer used in microfluidic device fabrication, polydimethylsiloxane, to absorb hydrophobic molecules such as Aβ_42_ and change their effective concentration (*70*). Vehicle controls consisted of a DMSO/F12 mixture, which was similarly incubated overnight.

### Axonal siRNA transfection

siRNAs were delivered at a final concentration of 50 nM using NeuroPORTER transfection reagent (Genlantis) 24 hours before Aβ_42_ treatment, as previously described (*19*, *20*). siRNA/NeuroPORTER mixture was prepared in serum- and antibiotic-free Neurobasal medium. Two hours after transfection, axonal compartments were supplemented with an equal volume of Neurobasal medium containing 2× B27 and 4 mM l-glutamine. *Stealth* RNA interference (RNAi) predesigned siRNAs were used (Thermo Fisher Scientific); *CREB3L2*: 5′-CGAGGGCUAUCCCAUUCCAACCAAA-3′ (RSS324942) and *HIF1A*: 5′-GCUAACAGAUGAUGGUGACAUGAUU-3′ (RSS310066). A control siRNA was similarly purchased from Thermo Fisher Scientific (Stealth RNAi siRNA Negative Control Med GC Duplex #3).

### Cell death assay (TUNEL)

Labeling of apoptotic cells was performed in accordance with the manufacturer’s instructions (DeadEnd Fluorometric TUNEL System, Promega). Cells were fixed in prewarmed 4% paraformaldehyde, 4% sucrose phosphate-buffered saline (PBS) solution for 20 min at room temperature. After washing with PBS, a bovine serum albumin (BSA; 3 mg ml^−1^), 100 mM glycine, 0.25% Triton X-100, PBS-based solution was incubated for 30 min at room temperature to permeabilize cells and block nonspecific binding. Nuclei were counterstained with 4′,6-diamidino-2-phenylindole (DAPI), and samples were mounted in ProLong Diamond Antifade Mountant (Thermo Fisher Scientific). Per replicate/condition, TUNEL (terminal deoxynucleotidyl transferase–mediated deoxyuridine triphosphate nick end labeling)–positive nuclei were scored against total cell number in no fewer than 10 fields situated in the vicinity of microgroove barriers.

### In vitro translation and CREB3L2-ATF4 coimmunoprecipitation

Capped, poly(A)-tailed *Homo sapiens CREB3L2* (HA- or V5-tagged, cleaved form) and *ATF4* (HA- or V5-tagged; engineered from Addgene plasmid #26114, gifted by Y. Ye) transcripts were synthesized using the mMESSAGE mMACHINE SP6 and Poly(A) Tailing kits from Thermo Fisher Scientific. RNAs were column-purified in accordance to the manufacturer’s instructions (RNeasy Mini Kit, QIAGEN), and efficient poly(A) tailing was evaluated by agarose gel electrophoresis. CREB3L2 and ATF4 translation reactions were incubated separately for 90 min at 30°C following vendor’s guidelines (Rabbit Reticulocyte Lysate System, Promega). CREB3L2 and ATF4 protein products were confirmed by immunoblotting in pilot experiments. Upon completion of the translation protocol, lysates (45 μl) containing CREB3L2 and ATF4 were mixed and incubated at 37°C for 30 min with gentle agitation (300 rpm for 5 s every minute) before immunoprecipitation, as previously described (*71*). Immunoprecipitation was carried out overnight at 4°C with gentle rotation in PBS supplemented with 0.1% NP-40 and protease inhibitors (cOmplete, EDTA-free Protease Inhibitor Cocktail, Roche), using magnetic beads conjugated with anti-HA or anti-V5 antibodies (anti-HA beads: PI88836, Thermo Fisher Scientific; anti-V5 beads: NC0777490, MBL International). Washing cycles were repeated five times with immunoprecipitation buffer. Complex elution was performed in 2× Laemmli buffer [130 mM tris-Cl (pH 6.8), 0.1 mM dithiothreitol, 20% (v/v) glycerol, and 4% SDS diluted in water] by boiling at 95°C for 5 min. Immunoblot detection of tagged CREB3L2 and ATF4: anti-HA (1:4000; ab9110, Abcam), anti-V5 (R960-25, Thermo Fisher Scientific), and anti–glyceraldehyde-3-phosphate dehydrogenase (GAPDH) (1:10,000; 60004-1-Ig, Proteintech), in conjugation with TrueBlot anti-rabbit IgG horseradish peroxidase (HRP) (1:1000; Rockland) or Superclonal anti-mouse IgG HRP (1:10,000; A28177, Thermo Fisher Scientific).

### Western blot

Unless otherwise specified, protein extracts were prepared in ice-cold radioimmunoprecipitation assay buffer [150 mM NaCl, 1% NP-40, 0.5% sodium deoxycholate, 0.1% SDS, and 50 mM tris (pH 8.0)] and resolved by SDS–polyacrylamide gel electrophoresis using the NuPAGE electrophoresis system (Thermo Fisher Scientific). Both “semi-dry” and “wet” electroblotting methods were used, depending on protein size, the latter being, however, our routine choice. Nitrocellulose membranes (0.2 or 0.45 μm; Amersham Protran, GE Healthcare) were blotted using the following primary antibodies: anti-CREB3L2 (1:1000; HPA015068, Atlas Antibodies), anti-ATF4 (1:1000; #11815, Cell Signaling Technology), anti-ATF4 (1:1000; ab184909, Abcam), anti-ATF4 (1:1000; WH0000468M1, MilliporeSigma) or anti-ATF4 [at 1:1000 (product discontinued); ab50546, Abcam], anti–green fluorescent protein (GFP) (1:2000; ab290, Abcam), and anti-VPS26 (1:1000; ab23892, Abcam); anti-VPS29 (1:500; ab98929, Abcam); anti-VPS35 (1:2500; ab10099, Abcam); anti-SNX1 (1:10,000; ab134126, Abcam); anti-SNX3 (1:500; ab56078, Abcam), anti-RAB7A (1:1000; #9367, Cell Signaling Technology), anti-EHD1 (1:2500; ab109311, Abcam), anti-APP (1:20,000; ab32136, Abcam), anti–N-cadherin (1:1000; #13116, Cell Signaling Technology), anti–CI-M6PR (1:30,000; ab124767, Abcam), anti-Tau (1:1000; clone HT7, MN1000, Invitrogen), anti–phospho-Tau (pSer^202^/pThr^205^; 1:4000; AT8, Invitrogen), anti–phospho-Tau (pSer^396^/pSer^404^; 1:7,000; PHF-1, formerly available through P. Davies), anti–phospho-Tau (pSer^404^; 1:1000; T7444, MilliporeSigma), anti-FLAG (1:1000; F1804, MilliporeSigma), anti–βIII-tubulin (1:10,000 to 1:20,000; #MA1-118, Thermo Fisher Scientific); anti–β-actin (1:10,000; #3700, Cell Signaling Technology) or anti–β-actin (1:10,000; MAB1501, MilliporeSigma); anti-GAPDH (1:10,000; 60004-1-Ig, Proteintech); anti-HDAC1 (1:10,000; ab109411, Abcam). Signals were visualized on KwikQuant Imager (Kindle Biosciences) and quantified using Fiji/ImageJ.

### PLA on primary neuronal cultures

Cells grown in glass-bottom dishes (P50G-1.5-30-F, MatTek Corporation) were fixed in prewarmed 4% paraformaldehyde and 4% sucrose in PBS (pH 7.4) for 20 min at room temperature. After repeated wash cycles, 0.25% Triton X-100 saline was added for 10 min to permeabilize membranes. Antigen retrieval with steaming 0.01 M sodium citrate (0.05% Tween 20, pH 6.0) was then carried out for 1 min. Blocking was performed for 1 hour with 5% heat-inactivated goat serum diluted in PBS, and primary antibodies, prepared in blocking solution, were incubated overnight at 4°C [anti-CREB3L2: 1:100, HPA015068, Atlas Antibodies; anti-ATF4: ab50546, Abcam (product discontinued), at 1:1000, or WH0000468M1, MilliporeSigma, at 1:100, or 60035-1-Ig, Proteintech, at 1:100]. PLA protocol was performed using Duolink In situ Red Detection reagents (DUO92008, MilliporeSigma) as per the manufacturer’s guidelines. Plus (DUO92002) and minus (DUO92004) probes were diluted 1:5 in blocking buffer and incubated at 37°C for 1 hour in a hybridization oven (Hoefer Red Roller II). After the last wash step of the Duolink protocol, neurons were counterstained with Alexa Fluor 488–conjugated βIII-tubulin antibody (1:500; #801203, BioLegend) diluted in a water-based 2 mM tris and 1 mM NaCl solution (pH 7.5) for 1 hour and preserved in Duolink In Situ Mounting Medium with DAPI (DUO82040, MilliporeSigma). All incubations were performed in a humidity chamber. Samples were imaged using an Axio Observer.Z1 microscope (Zeiss) equipped with an EC Plan-Neofluar 40×/1.3 oil objective and an AxioCam MRm Rev. 3 camera; alternatively, an LSM800 confocal microscope (Zeiss) with a Plan Apo 63×/1.4 oil objective was used for analysis of nuclear interactions. Imaging settings were kept constant between conditions. Signals were counted manually and, when applicable, normalized to axon length.

### 5xFAD transgenic animals: Care and PLA

All experiments were reviewed and monitored by the IACUC at Columbia University in accordance with National Institutes of Health (NIH) guidelines for the humane treatment of animals. Hemizygous 5-week-old B6SJL-Tg(APPSwFlLon,PSEN1*M146L*L286V)6799Vas/Mmjax (5xFAD) mice (Mutant Mouse Resource and Research Center stock no.: 34840-JAX) were purchased from The Jackson Laboratory. This strain overexpresses both mutant human APP with the Swedish (K670N and M671L), Florida (I716V), and London (V717I) familial AD (FAD) mutations and human presenilin 1 (PS1) harboring two FAD mutations, M146L and L286V. Age- and sex-matched wild-type B6SJLF1/J mice (stock no.: 100012) were acquired from The Jackson Laboratory at the same time. Both groups were maintained in our breeding colony until 10 weeks of age. Mice were euthanized following ketamine (80 to 100 mg/kg) and xylazine (5 to 10 mg/kg) administration, perfused with normal saline (McKesson, #37-6280), and fixed with 4% (v/v) paraformaldehyde. Brains were postfixed overnight in 4% paraformaldehyde, washed in PBS, transferred to 30% sucrose, and finally embedded for cryostat sectioning (12-μm-thick coronal cuts). Epitope unmasking was done for 20 min in steaming 0.01 M sodium citrate buffer (0.05% Tween 20, pH 6.0), which was followed by three 10-min PBS-T (0.1% Tween 20) washes. A standard prerequisite of the PLA protocol in its kit format is the availability of specific primary antibodies raised in different hosts. Because we were unable to locate a compatible pair of CREB3L2 and ATF4 antibodies, we resorted to using the Duolink Probemaker kits (DUO92009 and DUO92010, Sigma-Aldrich) to directly conjugate two rabbit-raised antibodies, anti-CREB3L2 (HPA015068, Atlas Antibodies) and anti-ATF4 (ab184909, Abcam), with PLA PLUS and MINUS oligonucleotides. This approach requires that both antibodies are solubilized in a carrier- and preservative-free buffer; to achieve this, we dialyzed the antibodies by using a Slide-A-Lyzer device with a 10,000 molecular weight cutoff (#69570, Thermo Fisher Scientific) made to float on a glass beaker containing 200 ml of PBS for 2 hours. The whole protocol was performed inside a cold room to minimize degradation and, in the case of the anti-CREB3L2 antibody, was followed by a concentration step (#88513, Thermo Fisher Scientific). The conjugation reaction was performed overnight at room temperature, and CREB3L2-ATF4 heterodimers were stained using Duolink In situ Far-red Detection reagents (DUO92013, MilliporeSigma). As per the manufacturer’s instructions, the PLA Probe Diluent included in the Probemaker kit was used in substitution of the PLA Antibody Diluent in the PLA protocol. Tissue sections were preserved in Duolink In situ Mounting Medium with DAPI (DUO82040, MilliporeSigma). Images were acquired on a LSM800 confocal microscope (Zeiss) with a Plan-Apochromat 40×/1.3 Oil DIC M27 oil objective (Zeiss). Imaging settings were kept constant between conditions. PLA interactions were unbiasedly analyzed in Fiji using the “Analyze Particles” function after autothresholding (“Yen” method). We excluded one 5xFAD animal due to technical difficulties in the tissue preparation phase; sample size was determined by power analysis.

### Chemically induced proximity: Reagent preparation

C-terminal FRB (T82L mutant) or FKBP (wild-type) fusions, N-terminally tagged with HA or V5 epitopes, respectively, were synthesized by Genewiz and transferred into pGL4.75[*hRluc*/CMV] (Promega) using Sac I and Fse I sites. Human CREB3L2 (residues 2 to 384, corresponding to its transcriptionally active form) and full-length ATF4 (S219A stabilization mutant) were used in transgene design (*72*, *73*); control *Renilla* luciferase sequence was obtained from pGL4.75 (Promega). Successful insertions were screened by Sanger sequencing. For neuronal expression, these same transgenes were cloned into a modified FUGW plasmid (Addgene, #14883, gift by D. Baltimore), in which the GFP open reading frame was substituted by a custom multiple cloning site (CTCTAGAGGATCCCCGGGTACCGGTGGCGCGCCGCTTAGCGTTAACGCTAGCCGGACCGCCTGCAGGAGGCCTGCCCGGGCATTTAAATGAATTCAAC; fragment synthesized by Genewiz) using Bam HI and Eco RI sites. Lentiviral particles were harvested from HEK293T cells after Lipofectamine 3000–mediated transfection with lentiviral and packaging plasmids (pCMVD R8.9 and pHCMV VSVg); 6 hours after transfection, the medium was changed to Neurobasal containing B27 and l-glutamine. Viral supernatant was collected 18 to 24 hours later, centrifuged 500*g* for 5 min, passed through a 0.45-μm polyethersulfone (PES) filter, aliquoted, and stored at −80°C. A/C heterodimerizer [C-16-(S)-7-methylindolerapamycin (AP21967)] was purchased from Takara.

### ChIPmera: ChIP and data analysis

HEK293T cells (#CRL-3216, American Type Culture Collection), plated in 150-mm dishes (CLS430599, Corning), were maintained in Dulbecco’s modified Eagle’s medium supplemented with 10% fetal bovine serum plus antibiotics (50 U ml^−1^ penicillin-streptomycin) and transfected using Lipofectamine 3000 (Thermo Fisher Scientific). The use of HEK293 cells in this analysis reflects the need to use substantial amounts of starting material. Amounts of DNA delivery were optimized to achieve comparable expression levels between the different homodimer and heterodimer configurations (ATF4 transgenes: 18.35 μg; CREB3L2 transgenes: 4.5 μg; luciferase transgenes: 2.3 μg), as ATF4 and CREB3L2 have markedly different half-lives in HEK293T cells (note that ATF4 is actively degraded in specific phases of the cell cycle) (*73*). While Lipofectamine complexes were incubating, a complete media change was performed, which now included the A/C heterodimerizer at a concentration of 500 nM. Twenty-four hours after transfection, we proceeded by cross-linking protein-DNA contacts with 1% (v/v) formaldehyde (#28908, Thermo Fisher Scientific) for 10 min at room temperature. After quenching cross-linking reaction with glycine, cells were washed twice with ice-cold PBS, harvested by scraping in PBS with protease inhibitors, and centrifuged at 2000*g* and 4°C, as per the manufacturer’s instructions (SimpleChIP Plus Kit, #9005, Cell Signaling Technology). Chromatin fragments (mainly one to three nucleosomes in size) were obtained by incubation with micrococcal nuclease [3.5 μl (equivalent to 7000 gel units) in 200 μl; M0247S, New England Biolabs] for 45 min in a 37°C water bath. Nuclear membranes were subsequently broken up by three rounds of 20-s, 15% amplitude pulses using Sonic Dismembrator Model 500 (Thermo Fisher Scientific), and lysates were clarified by centrifugation. Adequate digestion was assessed by agarose gel electrophoresis. For each condition, digested chromatin was split into two tubes and immunoprecipitated overnight at 4°C with end-over-end rotation using prewashed magnetic beads conjugated with anti-HA or anti-V5 antibodies (anti-HA beads: PI88836, Thermo Fisher Scientific; anti-V5 beads: NC0777490, MBL International); 35 μl of anti-HA beads and 25 μl of anti-V5 beads were used per immunoprecipitation. Beads were captured on a magnetic stand and washed with low- and high-salt buffers, as directed. Elution was performed at 65°C and 1200 rpm for 30 min using a thermomixer, protein-DNA cross-links were reversed by treatment with proteinase K for 2 hours at 65°C, and DNA was column-purified. A representative 2% input sample was prepared by combining chromatin from the different backgrounds. ChIP-seq library preparation and sequencing reactions were conducted at GENEWIZ Inc., as described above. A total of 24 samples were submitted for analysis, consisting of parallel HA and V5 immunoprecipitations, from two independent replicates. Sequencing libraries were multiplexed and clustered on two lanes of a flowcell. Sequencing was performed using a 2 × 150 paired-end configuration. ChIP-seq sequencing data were processed and analyzed within the Galaxy web platform. First, library adapters and low-quality reads were removed using Trim Galore (version 0.6.3) with the following settings: phred quality score threshold = 20, overlap with adapter sequence required to trim a sequence = 2, maximum allowed error rate = 0.1; reads shorter than 36 bp were additionally discarded. Second, reads were mapped to the hg38 reference genome with Bowtie 2 v2.3.4.1. Third, unmapped and low-quality [Mapping Quality (MAPQ) < 20] reads were excluded with samtools v1.8. Fourth, files were converted to bigWig format using bamCoverage v3.3.0, and signals were visualized in UCSC genome browser. Fifth, after running MACS predictd function, peak calling was performed with MACS2 v2.1.1.2 with minimum false discovery rate (FDR) cutoff for peak detection fixed at 0.01 and luciferase homodimers were defined as controls. Sixth, MACS2 output was filtered to exclude peaks with fold enrichments lower than 5. Seventh, differential binding analysis was performed on pooled replicated samples using DiffBind package v2.10.0 with FDR threshold set at 0.01. Eighth, genomic context was analyzed using ChIPseeker v1.18.0 against GENCODE v32/GRCh38 genome assembly (September 2019 release) and gene annotations assigned by GREAT v4.0.4; the cutoff for proximal promoter/enhancer regions was defined as ±3 kb from a transcription start site. HA and V5-ChIP-seq signal overlap assessment used the bedtools windowBed function (v2.29.0). GO analysis was performed using the GO Consortium database (geneontology.org). The MEME suite was used for motif discovery (*28*).

### Analysis of AD-associated and related transcriptional profiles

AD mRNA expression measurements and significance *P* values were obtained from publicly available datasets {E-GEOD-44770 (ArrayExpress identifier); GSE95587 [Gene Expression Omnibus (GEO)] and GSE15222 (GEO)}, the ROSMAP cohort, and Genentech’s Myeloid Landscape web resource (*4*, *7*, *45*, *46*, *49*).

### Integration of wider CREB3L2-ATF4 transcription network with AD profiles

Overlapping hits among CREB3L2-ATF4 datasets were found using the Venn diagram module accessible at http://genevenn.sourceforge.net/. Processed ChIP-seq files [identifiers: ENCFF794DLT (NFE2L2), ENCFF516MEQ (NFATC1), and ENCFF353RDB (MXD4)], prepared and analyzed by the ENCODE Consortium, were retrieved from www.encodeproject.org. The DNA binding program of SOX9 was mined from published literature, and only “class I” sites were considered, cataloged as such by Ohba *et al*. (*35*) based on their clustering around transcription start sites. Gene annotations of ranked peaks were assigned by GREAT v4.0.4 using a ±3-kb transcription start site cutoff. For each dataset, only the top (i.e., strongest) 3000 hits within this cutoff were carried forward to accommodate the requirements of the Metascape analysis pipeline (*36*), which was used to produce the comparative GO meta-analysis. AD-associated gene expression changes were obtained from publicly available datasets (*4*, *7*). Top 3000 DEGs in AD prefrontal cortex (bulk RNA-seq dataset), excluding conflicting entries, were grouped according to expression profile, totaling 1267 up-regulated and 1692 down-regulated genes (lowest adjusted *P* value = 9.93 × 10^−20^).

### Neuronal culture supernatant collection and measurements

Culture supernatants were transferred to 15-ml falcon tubes, spun at 2000*g* and 4°C for 5 min, aliquoted, and stored at −80°C. A sandwich immunoassay (V-PLEX Aβ Peptide Panel 1 kit, #4G8, Meso Scale Discovery) was used in the measurement of β-amyloid species. Manufacturer’s guidelines were followed thoroughly during plate preparation, and samples were diluted 1:1 with Diluent 35 (provided as part of the kit) to avoid matrix saturation. All biological replicates were measured in parallel. Signal readings were performed on a Sector Imager 2400 instrument (Meso Scale Discovery). For assessing sAPPα levels in culture supernatants, we used a sandwich ELISA assay [sAPPα (mouse/rat) (highly sensitive), #27419, Immuno-Biological Laboratories], and samples were diluted 10-fold. Extracellular tau levels were assessed using the Phospho(Thr^231^)/Total Tau Kit from Meso Scale Discovery following the protocol provided by the manufacturer. Neurofilament-light and Map2 were measured by ELISA using PathScan Total Neurofilament-L Sandwich ELISA kit (#99175, Cell Signaling Technology) and Abcam’s SimpleStep MAP2 ELISA kit (ab253229), respectively.

### Analysis of Tau phosphorylation

DIV1 rat hippocampal neurons were infected with lentiviruses carrying FRB/FKBP-tagged ATF4 or CREB3L2 transgenes. One day later, the heterodimerizer was added to cultures diluted in Neurobasal growth medium at 100 nM, and cells were allowed to mature until DIV10. Additional rounds of heterodimerizer supplementation (100 nM, diluted in growth medium) were made every other day. On DIV10, cells were washed in ice-cold Hanks’ balanced salt solution and lysed in 2× Laemmli buffer [130 mM tris-Cl (pH 6.8), 0.1 mM dithiothreitol, 20% (v/v) glycerol, and 4% SDS diluted in water] by scrapping. Fresh lysates were boiled at 85°C for 5 min and immediately analyzed by Western blot. The following antibodies were used: anti-Tau (1:1000; clone HT7, MN1000, Invitrogen), anti–phospho-Tau (pSer^202^/pThr^205^; 1:4000; AT8, Invitrogen), anti–phospho-Tau (pSer^396^/pSer^404^; PHF-1, formerly available through P. Davies), and anti–phospho-Tau (pSer^404^; 1:1000; T7444, MilliporeSigma).

### PP2A activity assay

Hippocampal neurons were initially lysed in accordance with the instructions provided in the Serine/Threonine Phosphatase Assay Kit (V2460, Promega). Lysates were then centrifuged at 1 × 10^5^ *g* at 4°C for 1 hour in phosphatase storage buffer [2 mM EGTA, 5 mM EDTA, 0.5 mM phenylmethylsulfonyl fluoride, 150 mM NaCl, 1% Triton X-100, 50 mM tris-HCl (pH 7.4), and 0.5% protease inhibitor cocktail]. Sephadex G-25 spin columns were used to remove free phosphate found endogenously, followed by incubation for 1 hour at 37°C in PP2A reaction buffer [250 mM imidazole (pH 7.2), 1 mM EGTA, 0.1% β-mercaptoethanol, and BSA (0.5 mg/ml)] supplemented with Ser/Thr phosphopeptide. The reaction was stopped by adding 50 μl of molybdate dye/additive mixture. After 30 min, absorbance was measured at 600 nm in a 96-well microplate reader (Tecan). PP2A activity measurements were normalized to the total DNA content in biological replicates using a CyQUANT assay (Thermo Fisher Scientific). PP2B and PP2C show very low to no detectable activity in the presence of EGTA (PP2B) and EDTA (PP2C); it is also noteworthy that the phosphopeptide used in this assay is a poor substrate for protein phosphatase 1.

### Human brain sample procurement

Postmortem human material was obtained through the New York Brain Bank at Columbia University and the Neuropathology Brain Bank at Mount Sinai according to institutional guidelines governed by approved protocols. Neuropathological evaluations (tables S1 to S3) included assignment of CERAD, Braak, NIA-Reagan, or ABC scores. Dorsolateral prefrontal cortex tissue specimens were derived from Brodmann area 9.

### CREB3L2-ATF4 coimmunoprecipitation in human brain tissue

Protein A magnetic beads (#S1425S, New England Biolabs) were washed in PBS containing 0.1% BSA and incubated at 4°C for 1 hour with rotation. Following two rinses with PBS, beads were resuspended in lysis buffer, mixed for 4 hours with anti-ATF4 antibody (1 μg per immunoprecipitation; ab184909, Abcam), and washed three times with lysis buffer. At this point, we proceeded by covalently cross-linking the immobilized antibodies to protein A beads using bis(sulfosuccinimidyl)suberate (BS^3^; #21586, Thermo Fisher Scientific) following the manufacturer’s guidelines. Frozen dorsolateral prefrontal cortex tissue (approximately 80 mg per immunoprecipitation; table S1) was processed in ice-cold lysis buffer [20 mM tris-Cl (pH 8), 137 mM NaCl, 1% NP-40, 2 mM EDTA, supplemented with protease and phosphatase inhibitors (cOmplete cocktail tablets, Roche)]. Sample volumes were weight-adjusted in a sample-by-sample manner, and tissue extracts were incubated for 2 hours at 4°C with end-over-end rotation. During this incubation, a 10-min bath sonication step was performed to improve extraction efficiency. After centrifugation at 12,000 rpm and 4°C, pellets were discarded and supernatants were transferred to new tubes. Equal amounts of antibody-bead conjugates were mixed with lysates overnight at 4°C with constant rotation and washed a total of four times with ice-cold lysis buffer. Immunoprecipitation input and the supernatant resulting from the first wash step (“flow-through” fraction) were saved for further analyses. Immunoprecipitates were eluted in 50 μl of 0.2 M glycine buffer (pH 2.5) and allowed to react for 5 min at 4°C with rotation after a short vortexing step. Eluates were transferred to a new tube, and the elution protocol was repeated. Pooled eluates were neutralized by the addition of 20 μl of 1 M tris-Cl (pH 9.0), and Laemmli buffer–treated samples were heated at 80°C for 5 min. CREB3L2 signals were visualized using anti-CREB3L2 serum (HPA015068, Atlas Antibodies) and a light chain–specific monoclonal secondary antibody (211-032-171, Jackson ImmunoResearch); successful ATF4 immunoprecipitation was confirmed using anti-ATF4 sera (ab184909, Abcam, and #11815, Cell Signaling Technology).

### PLA in AD prefrontal cortex

CREB3L2-ATF4 heterodimers were visualized using Duolink In Situ Brightfield Detection reagents (DUO92012, MilliporeSigma). CREB3L2 and ATF4 PLA probes were prepared as described for the detection of CREB3L2-ATF4 heterodimers in 5xFAD mice. Per manufacturer’s instructions, the PLA Probe Diluent included in the Probemaker Kit was used in substitution of the PLA Antibody Diluent in the PLA protocol. Before deparaffinization with xylene, slides were placed in a 60°C oven for 1 hour; we proceeded by rehydrating slides using a graded ethanol series (100% > 95% > 70% > 50% > water) plus two 10-min PBS-T washes. Epitope unmasking was done for 20 min in steaming tris-EDTA buffer [10 mM tris base, 1 mM EDTA, and 0.05% Tween 20 (pH 9.0)], followed by three 5-min PBS-T rinses.

We quenched endogenous peroxidases slides with 1% hydrogen peroxide for 30 min before blocking. Costaining of neurofilament (1:400; heavy chain subunit; #N0142, MilliporeSigma) was performed afterward using the Vector Blue Alkaline Phosphatase Substrate Kit (SK-5300, Vector Laboratories). To increase detection sensitivity, we additionally used the Vectastain ABC-AP system (AK-5002, Vector Laboratories) before signal development. Last, sections were dehydrated in a graded ethanol series (50% > 70% > 95% > 100%), cleared with Histo-Clear (64110-01, Electron Microscopy Sciences), mounted in VectaMount (H-5000, Vector Laboratories), and air-dried for 24 hours before proceeding with imaging. Human dorsolateral prefrontal cortex specimens (Brodmann area 8/9; table S2) were manually counted by an experimenter “blind” to the underlying diagnosis. Technical controls: PLA Probe Rabbit IgG Isotype Control MINUS (DUO87004, MilliporeSigma) and CREB3L2 blocking peptide (APrEST73339, Atlas Antibodies). For each case, CREB3L2-ATF4 measurements were interspersed between five randomly selected tissue subregions; specifically, 10 neurons within layers III to V were analyzed in each subregion, for a total of 50 independent measurements per brain.

### Statistical analyses

Each experiment was independently repeated at least three times unless otherwise indicated. Individual measurements were taken from distinct samples. Details of biological replication and statistical analysis are indicated in figure legends or main text. For all tests, a significance level (α) of 0.05 was used. Datasets were analyzed with Prism (GraphPad). Detailed statistical results are provided in table S6. Representation factors and associated probabilities were obtained using a web-based resource (http://nemates.org/MA/progs/overlap_stats.html) developed by J. Lund (*74*). Briefly, representation factors are a measure of overlap between two independent groups of genes that quantifies “actual” versus “expected” occurrences; for our purposes, 25,000 was assumed as the total number of genes in the human genome. The probability of finding a certain level of overlap was calculated via hypergeometric statistics.

## Notes

View/request a protocol for this paper from *Bio-protocol*.
