# Supplementary material for: CREB3L2-ATF4 heterodimerization defines a transcriptional hub of Alzheimer’s disease gene expression linked to neuropathology
Source: Sci Adv. 2023 Mar 3;9(9):eadd2671. doi: 10.1126/sciadv.add2671 (PMC9984184; doi:10.1126/sciadv.add2671)
Supplement: Supplementary file 1 — Figs. S1 to S11 Tables S1 to S6 Supplementary Materials and Methods [file sciadv.add2671_sm.pdf]

Science Advances
AAAS

Supplementary Materials for

CREB3L2-ATF4 heterodimerization defines a transcriptional hub of Alzheimer's disease gene expression linked to neuropathology

Cláudio Gouveia Roque *et al.*

Corresponding author: Ulrich Hengst, uh2112@cumc.columbia.edu

Sci. Adv. **9**, eadd2671 (2023)
DOI: 10.1126/sciadv.add2671

This PDF file includes:

Figs. S1 to S11
Tables S1 to S6
Supplementary Materials and Methods

Figure S1.

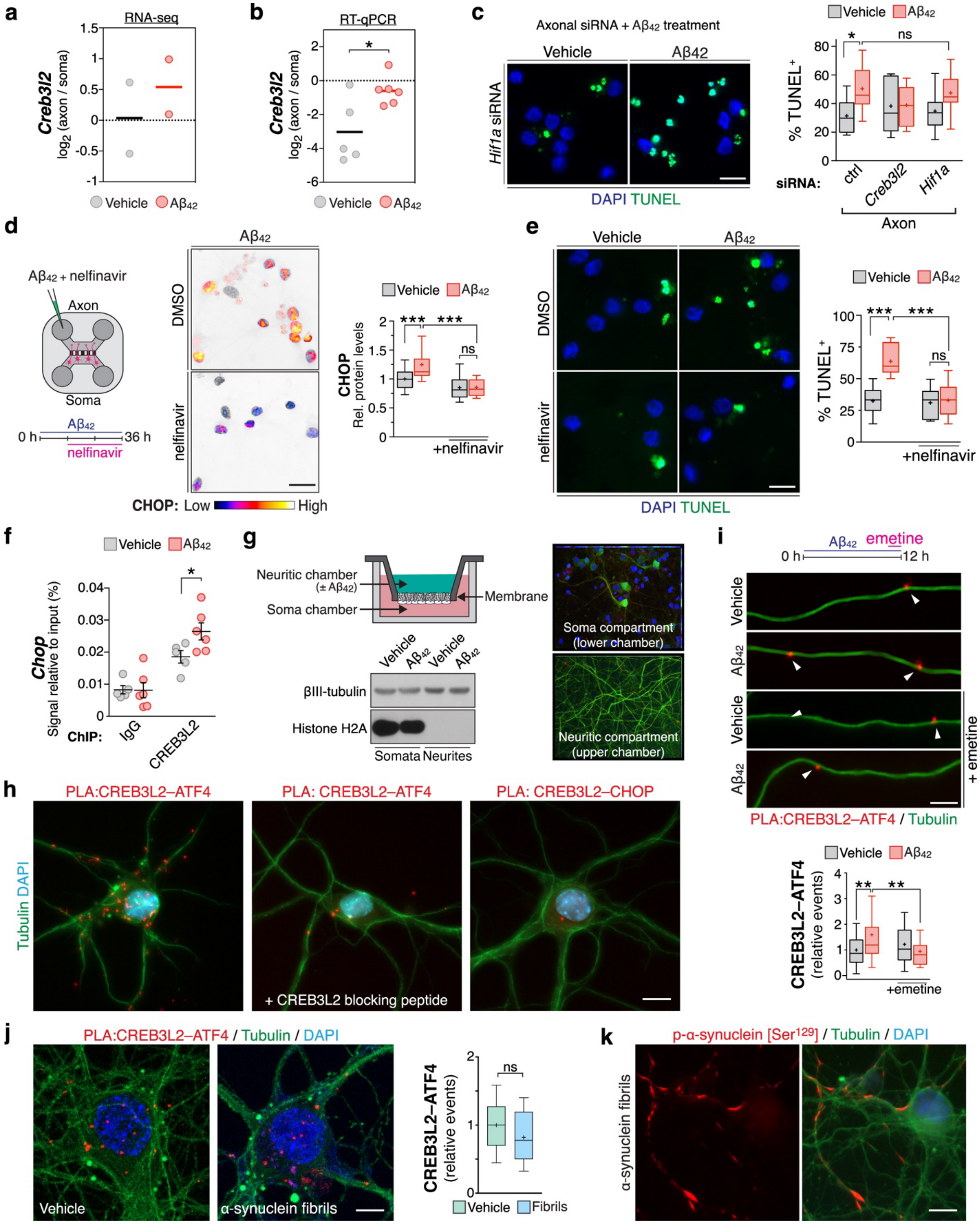

a
RNA-seq
Creb3l2
log2 (axon / soma)
Vehicle
Aβ42
b
RT-qPCR
c
Axonal siRNA + Aβ42 treatment
Vehicle
Aβ42
Hif1a siRNA
DAPI TUNEL
% TUNEL+
siRNA:
ctrl
Creb3l2
Hif1a
Axon
ns
d
Aβ42 + nelfinavir
Axon
Soma
0 h
36 h
nelfinavir
DMSO
CHOP: Low High
CHOP
Rel. protein levels
+nelfinavir
e
f
Chop
Signal relative to input (%)
ChIP:
IgG
CREB3L2
g
Neuritic chamber
(± Aβ42)
Soma chamber
Membrane
Soma compartment
(lower chamber)
Neuritic compartment
(upper chamber)
βIII-tubulin
Histone H2A
Somata
Neurites
h
PLA:CREB3L2–ATF4
PLA: CREB3L2–ATF4
PLA: CREB3L2–CHOP
Tubulin DAPI
+ CREB3L2 blocking peptide
i
emetine
0 h
12 h
+ emetine
PLA:CREB3L2–ATF4 / Tubulin
CREB3L2–ATF4
(relative events)
+emetine
j
PLA:CREB3L2–ATF4 / Tubulin / DAPI
Vehicle
α-synuclein fibrils
Fibrils
k
p-α-synuclein [Ser129] / Tubulin / DAPI
α-synuclein fibrils

Fig. S1 – $A\beta_{42}$ promotes CREB3L2–ATF4 heterodimerization.

a. Axonal *Creb3l2* expression following $A\beta_{42}$ stimulation of hippocampal neurons grown under microfluidic isolation. This analysis was performed using an RNA-seq dataset (GSE70860) previously published by our laboratory (*19*). $\log_2$-transformed data points are normalized to somatic *Creb3l2* levels.

b. RT-qPCR analysis of axonal *Creb3l2* expression following $A\beta_{42}$ stimulation of hippocampal neurons grown under microfluidic isolation. Log_2-transformed data points are normalized to somatic *Creb3l2* levels. Summary of $n = 4$ replicates; **P*-value =0.0199, unpaired *t*-test.

c. TUNEL assay of rat hippocampal neurons after $A\beta_{42}$ stimulation. Axons were transfected with control, *Creb3l2*, or *Hif1a*-targeting siRNAs before treatment with $A\beta_{42}$ for 48 hours. *Hif1a* is another TF-encoding gene whose mRNA is recruited into axons following $A\beta_{42}$ stimuli (*19*). Unlike *Creb3l2*, silencing axonal *Hif1a* expression did not ameliorate $A\beta_{42}$-induced cell death. Box-plots display summary of n = 10-12 replicates; **P*-value = 0.0357, one-way ANOVA test with post hoc Bonferroni multiple comparison correction. Scale bar, 15 μm.

d. Analysis of nuclear CHOP protein levels by quantitative immunofluorescence. Cells were cultured in microfluidic chambers for 10-12 DIV and $A\beta_{42}$ oligomers applied for 36 hours; nelfinavir, an S2P inhibitor, was added in the last 24 hours of the $A\beta_{42}$ protocol. Both treatments were delivered specifically to axons. Box-plot summary of $n = 3$ experiments; per replicate, each condition was sampled from 10 different optical fields; ****P*-value < 0.0001, one-way ANOVA test with post hoc Bonferroni multiple comparison correction. Scale bar, 15 μm.

e. Cell death analysis by TUNEL assay of rat hippocampal neurons. The experimental outline was the same as in (d). Box-plot summary of $n = 3$ independent experiments; per replicate, each condition was sampled from 10 different optical fields; ****P*-value < 0.0001, one-way ANOVA test with post hoc Bonferroni multiple comparison correction. Scale bar, 15 μm.

f. ChIP-qPCR assay of CREB3L2 binding to *Chop* regulatory DNA in vehicle- or $A\beta_{42}$-treated rat cortical neurons. Neurons were grown without microfluidic compartmentalization (i.e., dissociated cultures), and $A\beta_{42}$ was bath-applied for 36 hours. ChIP signals in each sample were normalized against total input chromatin. Summary of $n = 5$ independent replicates; ****P*-value < 0.0001, one-way ANOVA test with post hoc Bonferroni multiple comparison correction.

g. Schematic representation of the transwell Boyden chamber system employed to isolate neurites from cell bodies for biochemical analyses. To validate its applicability, we tested for the presence of a nuclear protein, Histone H2A, which was exclusively found in somatic extracts.

h. Representative control reactions for CREB3L2–ATF4 PLA signals in dissociated hippocampal neurons counterstained with βIII-tubulin and DAPI. CREB3L2 antibody and blocking peptide were co-incubated before proceeding with the assay. CHOP is not known to interact with CREB3L2 and, in line with this, a PLA targeting both transcription factors produces negligible signals. Scale bar, 10 μm.

i. Detection of axonal CREB3L2–ATF4 heterodimers by PLA after inhibition of local protein synthesis. Emetine was delivered to axons in the last 60 minutes of a 12-hour $A\beta_{42}$ stimulation protocol. Hippocampal neurons were cultured using a microfluidic chamber system to allow for axon-specific manipulations. Approximately 60 axonal fields were analyzed per condition, over $n = 3$ independent replicates; ****P*-value < 0.0001, one-way ANOVA test with post hoc Bonferroni multiple comparison correction. Scale bar, 10 μm.

j. Detection of neuronal CREB3L2–ATF4 heterodimers by PLA in dissociated hippocampal neurons. α-synuclein pre-formed fibrils were bath-applied for 10 days. βIII-tubulin and DAPI were used as counterstains. Box-plots display a summary of relative interaction events; 10 neurons were analyzed per condition in each experiment, over $n = 3$ independent replicates. Scale bar, 5 μm.

k. Immunofluorescence analysis of intraneuronal α-synuclein accumulation after 10-day incubation protocol. Scale bar, 10 μm.

Figure S2.

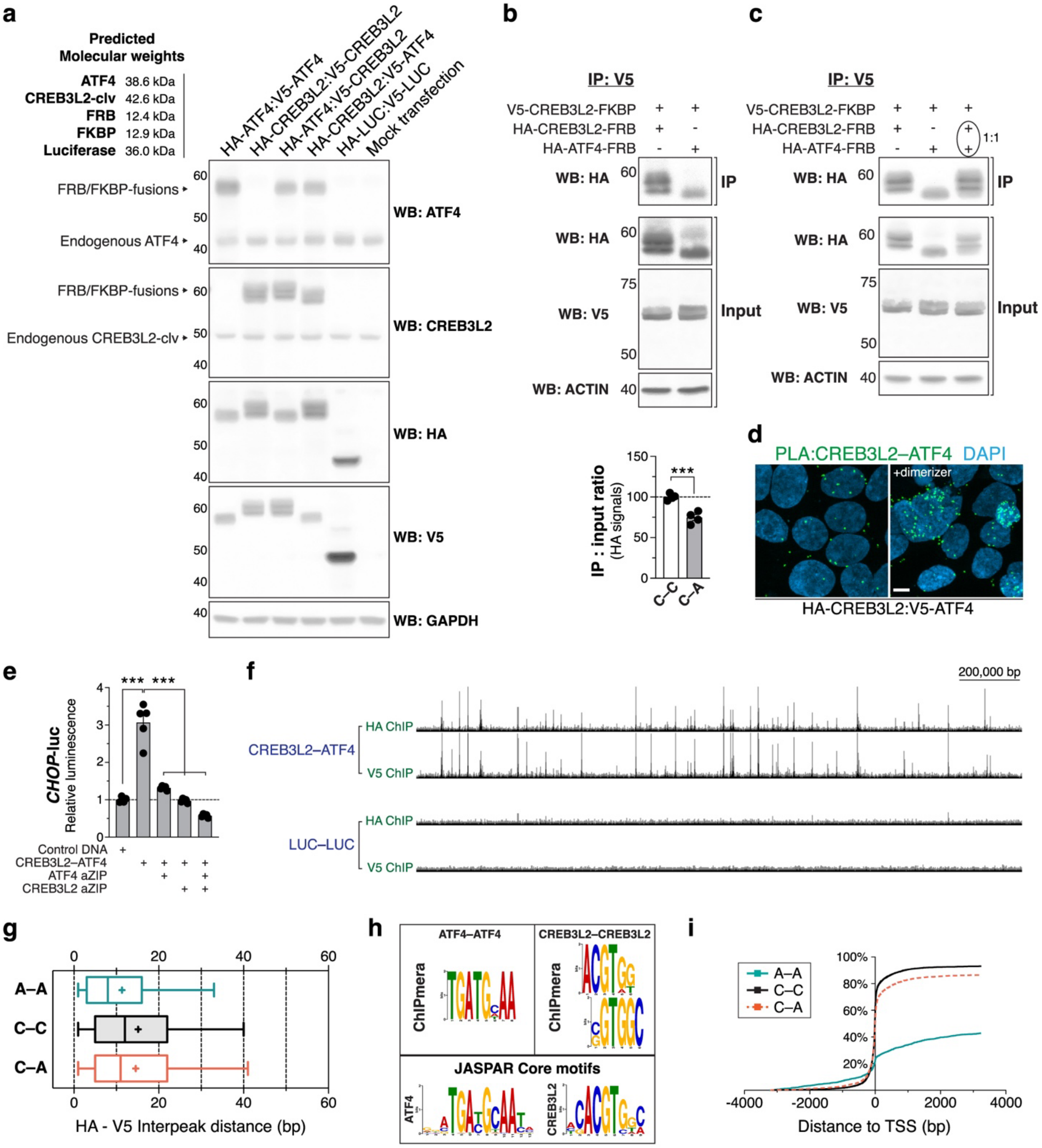

a
Predicted Molecular weights
ATF4 38.6 kDa
CREB3L2-clv 42.6 kDa
FRB 12.4 kDa
FKBP 12.9 kDa
Luciferase 36.0 kDa
HA-ATF4:V5-ATF4
HA-CREB3L2:V5-CREB3L2
HA-ATF4:V5-CREB3L2
HA-CREB3L2:V5-ATF4
HA-LUC:V5-LUC
Mock transfection
FRB/FKBP-fusions
Endogenous ATF4
WB: ATF4
Endogenous CREB3L2-clv
WB: CREB3L2
WB: HA
WB: V5
WB: GAPDH
b
IP: V5
V5-CREB3L2-FKBP
HA-CREB3L2-FRB
HA-ATF4-FRB
WB: HA
IP
Input
WB: V5
WB: ACTIN
IP : input ratio (HA signals)
C–C
C–A
c
1:1
d
PLA:CREB3L2–ATF4 DAPI
+dimerizer
HA-CREB3L2:V5-ATF4
e
CHOP-luc Relative luminescence
Control DNA
CREB3L2–ATF4
ATF4 aZIP
CREB3L2 aZIP
f
200,000 bp
CREB3L2–ATF4
HA ChIP
V5 ChIP
LUC–LUC
g
A–A
C–C
C–A
HA - V5 Interpeak distance (bp)
h
ATF4–ATF4
CREB3L2–CREB3L2
ChIPmera
JASPAR Core motifs
ATF4
CREB3L2
i
Distance to TSS (bp)

Fig. S2 – ChIPmera identifies DNA-binding patterns of dimeric TFs across the genome.

a. Western blot analysis of ChIPmera transgene expression in HEK293 cells. Different permutations of tagged TFs were co-expressed and probed 24 hours later with anti-CREB3L2, ATF4, HA, or V5 antibodies, revealing overall comparable expression levels between backgrounds.

b. Co-immunoprecipitation analysis of chemically induced CREB3L2–CREB3L2 (C–C) and CREB3L2–ATF4 (C–A) dimers in HEK293 cells. Mean ± SEM of $n = 4$ independent replicates, normalized to input HA signals; ****P*-value = 0.0009, unpaired *t*-test.

c. Co-immunoprecipitation analysis of chemically induced CREB3L2–CREB3L2 and CREB3L2–ATF4 dimers in HEK293 cells. Despite CREB3L2–ATF4 heterodimers establishing a less efficient association (and/or being more prone to degradation) than CREB3L2–CREB3L2 homodimers, ATF4 is still able to compete with CREB3L2 for binding, as indicated by its appreciable presence in V5-CREB3L2 immunoprecipitates when HA-ATF4 and HA-CREB3L2 are co-expressed.

d. PLA detection of chemically induced CREB3L2–ATF4 heterodimers in HEK293 cells with anti-CREB3L2 and anti-ATF4 antibodies before and after addition of dimerizer. DAPI (nuclear counterstain), 4′,6-diamidino-2-phenylindole. Scale bar, 10 μm.

e. *CHOP* luciferase reporter activities in CREB3L2–ATF4-expressing HEK293 cells. Plot shows mean ± SEM; ****P*-value < 0.0001, one-way ANOVA test with Dunnett's multiple comparison test.

f. Genome browser tracks for CREB3L2–ATF4 and control *Renilla* luciferase LUC–LUC dimers. This representative 2,000,000 bp genomic window on chromosome 1 (chr1:150,151,864-152,151,863) in the vicinity of *SNX27* shows various strong and coincident peaks for CREB3L2–ATF4. By contrast, LUC–LUC dimers produce neglectable ChIP-seq signals. Indeed, the MACS peak calling algorithm predicted, on average, just 12 significant peaks across the whole genome between the four LUC–LUC ChIP-seq experiments analyzed.

g. Analysis of HA and V5-ChIP-seq peak coincidence for each dimer pair. A–A, ATF4–ATF4; C–C, CREB3L2–CREB3L2; C–A, CREB3L2–ATF4.

h. Motif similarity analysis using the Tomtom algorithm. JASPAR Core (2018 version) is a curated database of TF DNA-binding preferences (http://jaspar.genereg.net/). ATF4–ATF4

versus JASPAR ATF4: *P*-value = 7.08×10^{-8}; CREB3L2–CREB3L2 versus JASPAR CREB3L2: *P*-value = 2.89×10^{-5}.

i. Cumulative frequency distribution of ChIPmera peaks proximal (<3 kb) to a transcription start site (TSS). A–A, ATF4–ATF4; C–C, CREB3L2–CREB3L2; C–A, CREB3L2–ATF4.

Figure S3.

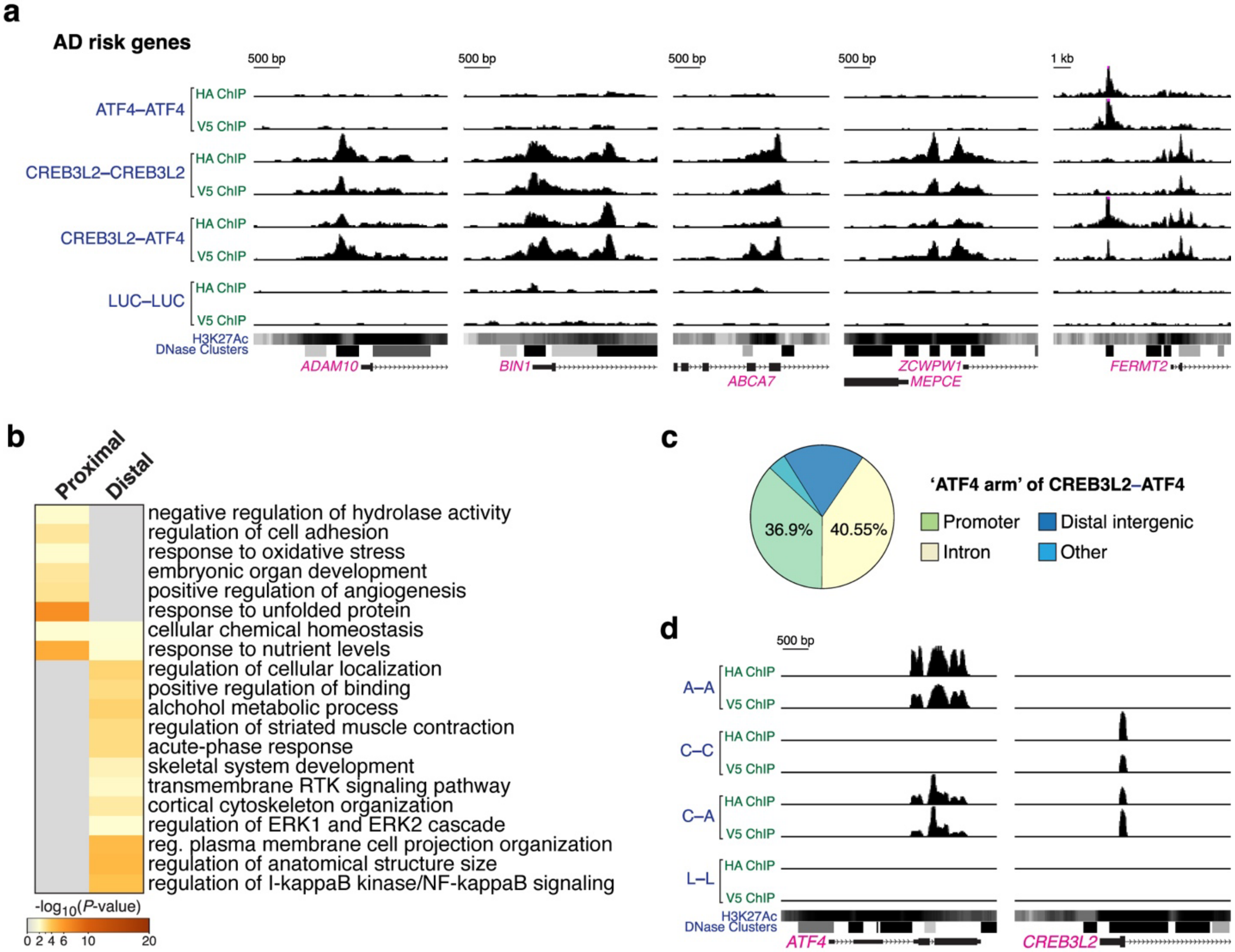
a
AD risk genes
500 bp
1 kb
ATF4–ATF4
CREB3L2–CREB3L2
CREB3L2–ATF4
LUC–LUC
HA ChIP
V5 ChIP
H3K27Ac
DNase Clusters
ADAM10
BIN1
ABCA7
ZCWPW1
MEPCE
FERMT2
b
Proximal
Distal
negative regulation of hydrolase activity
regulation of cell adhesion
response to oxidative stress
embryonic organ development
positive regulation of angiogenesis
response to unfolded protein
cellular chemical homeostasis
response to nutrient levels
regulation of cellular localization
positive regulation of binding
alchohol metabolic process
regulation of striated muscle contraction
acute-phase response
skeletal system development
transmembrane RTK signaling pathway
cortical cytoskeleton organization
regulation of ERK1 and ERK2 cascade
reg. plasma membrane cell projection organization
regulation of anatomical structure size
regulation of I-kappaB kinase/NF-kappaB signaling
-log10(P-value)
0 2 4 6 10 20
c
36.9%
40.55%
'ATF4 arm' of CREB3L2–ATF4
Promoter
Distal intergenic
Intron
Other
d
500 bp
A–A
C–C
C–A
L–L
HA ChIP
V5 ChIP
H3K27Ac
DNase Clusters
ATF4
CREB3L2

Fig. S3 – The CREB3L2–ATF4 transcriptional program.

a. ChIPmera genome browser tracks centered in AD susceptibility loci. bp, base pair; kb, kilobase.

b. GO functional analysis (biological process) of the CREB3L2–ATF4-specific genes (i.e., not shared with CREB3L2–CREB3L2). Genes were classified as 'proximal' or 'distal' depending on the proximity of its corresponding peak to the nearest transcription start site.

c. Genomic distribution of CREB3L2–ATF4 ChIPmera peaks not associated with CREB3L2–CREB3L2. The large majority of these (91.7%) coincide with ChIP-seq ATF4 signals described by the ENCODE Consortium. Cutoff for proximal promoter/enhancer regions was defined as ±3 kb from a transcription start site.

d. ChIPmera genome browser tracks in the vicinity of *CREB3L2* and *ATF4* loci juxtaposed with ENCODE-produced H3K27Ac and DNaseI hypersensitivity profiles. A–A, ATF4–ATF4; C–C, CREB3L2–CREB3L2; C–A, CREB3L2–ATF4; L–L, LUC–LUC.

Figure S4.

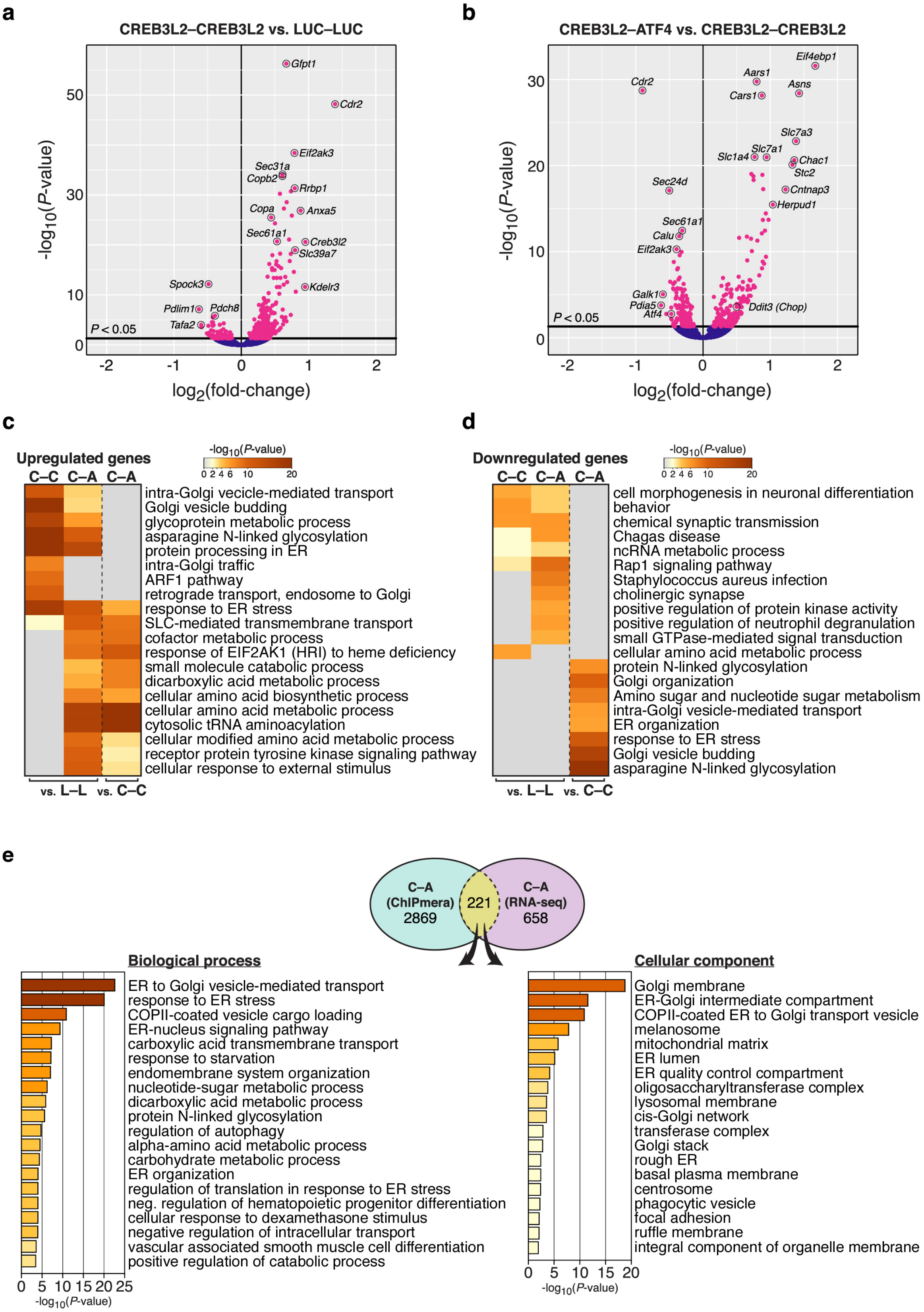

a
CREB3L2–CREB3L2 vs. LUC–LUC
-log10(P-value)
log2(fold-change)
P < 0.05
Gfpt1
Cdr2
Eif2ak3
Sec31a
Copb2
Rrbp1
Copa
Anxa5
Sec61a1
Creb3l2
Slc39a7
Kdelr3
Spock3
Pdlim1
Pdch8
Tafa2
b
CREB3L2–ATF4 vs. CREB3L2–CREB3L2
-log10(P-value)
log2(fold-change)
P < 0.05
Eif4ebp1
Aars1
Asns
Cdr2
Cars1
Slc7a3
Slc7a1
Slc1a4
Chac1
Stc2
Sec24d
Cntnap3
Herpud1
Sec61a1
Calu
Eif2ak3
Galk1
Pdia5
Atf4
Ddit3 (Chop)
c
Upregulated genes
-log10(P-value)
C–C
C–A
C–A
intra-Golgi vecicle-mediated transport
Golgi vesicle budding
glycoprotein metabolic process
asparagine N-linked glycosylation
protein processing in ER
intra-Golgi traffic
ARF1 pathway
retrograde transport, endosome to Golgi
response to ER stress
SLC-mediated transmembrane transport
cofactor metabolic process
response of EIF2AK1 (HRI) to heme deficiency
small molecule catabolic process
dicarboxylic acid metabolic process
cellular amino acid biosynthetic process
cellular amino acid metabolic process
cytosolic tRNA aminoacylation
cellular modified amino acid metabolic process
receptor protein tyrosine kinase signaling pathway
cellular response to external stimulus
vs. L–L
vs. C–C
d
Downregulated genes
-log10(P-value)
C–C
C–A
C–A
cell morphogenesis in neuronal differentiation
behavior
chemical synaptic transmission
Chagas disease
ncRNA metabolic process
Rap1 signaling pathway
Staphylococcus aureus infection
cholinergic synapse
positive regulation of protein kinase activity
positive regulation of neutrophil degranulation
small GTPase-mediated signal transduction
cellular amino acid metabolic process
protein N-linked glycosylation
Golgi organization
Amino sugar and nucleotide sugar metabolism
intra-Golgi vesicle-mediated transport
ER organization
response to ER stress
Golgi vesicle budding
asparagine N-linked glycosylation
vs. L–L
vs. C–C
e
C–A (ChIPmera) 2869
221
C–A (RNA-seq) 658
Biological process
ER to Golgi vesicle-mediated transport
response to ER stress
COPII-coated vesicle cargo loading
ER-nucleus signaling pathway
carboxylic acid transmembrane transport
response to starvation
endomembrane system organization
nucleotide-sugar metabolic process
dicarboxylic acid metabolic process
protein N-linked glycosylation
regulation of autophagy
alpha-amino acid metabolic process
carbohydrate metabolic process
ER organization
regulation of translation in response to ER stress
neg. regulation of hematopoietic progenitor differentiation
cellular response to dexamethasone stimulus
negative regulation of intracellular transport
vascular associated smooth muscle cell differentiation
positive regulation of catabolic process
-log10(P-value)
Cellular component
Golgi membrane
ER-Golgi intermediate compartment
COPII-coated ER to Golgi transport vesicle
melanosome
mitochondrial matrix
ER lumen
ER quality control compartment
oligosaccharyltransferase complex
lysosomal membrane
cis-Golgi network
transferase complex
Golgi stack
rough ER
basal plasma membrane
centrosome
phagocytic vesicle
focal adhesion
ruffle membrane
integral component of organelle membrane
-log10(P-value)

Fig. S4 – RNA-seq analysis of CREB3L2–ATF4-mediated transcriptional disruption.

a. Volcano plot depicting changes in gene expression triggered by CREB3L2–CREB3L2 homodimers in hippocampal neurons. Basal expression levels were measured in cells expressing *Renilla* luciferase homodimers. Fold-changes (log_2-transformed) and adjusted *P*-values ($-log_{10}$-transformed), as calculated by DESeq2 from $n = 5$ independent replicates, are plotted along the X- and Y-axes, respectively. Magenta data points: adjusted *P*-value < 0.05; blue data points: adjusted *P*-value > 0.05.

b. Same as in (a), except that here a head-to-head comparison between neurons expressing CREB3L2–ATF4 or CREB3L2–CREB3L2 dimers is shown.

c. Meta-enriched ontology clusters (top 20) across CREB3L2–CREB3L2, CREB3L2–ATF4, and control backgrounds, colored by *P*-values ($-log_{10}$-transformed). Only significantly upregulated genes were included in this analysis. The first two columns denote enrichments over control, while the third column shows a direct comparison between CREB3L2–ATF4 and CREB3L2–CREB3L2 neurons.

d. Same as in (d), except that downregulated genes were analyzed.

e. GO functional analyses of the 221 DEGs downstream of CREB3L2–ATF4 activation in neurons previously identified as direct DNA-binding targets of the heterodimer in our ChIPmera study.

Figure S5.

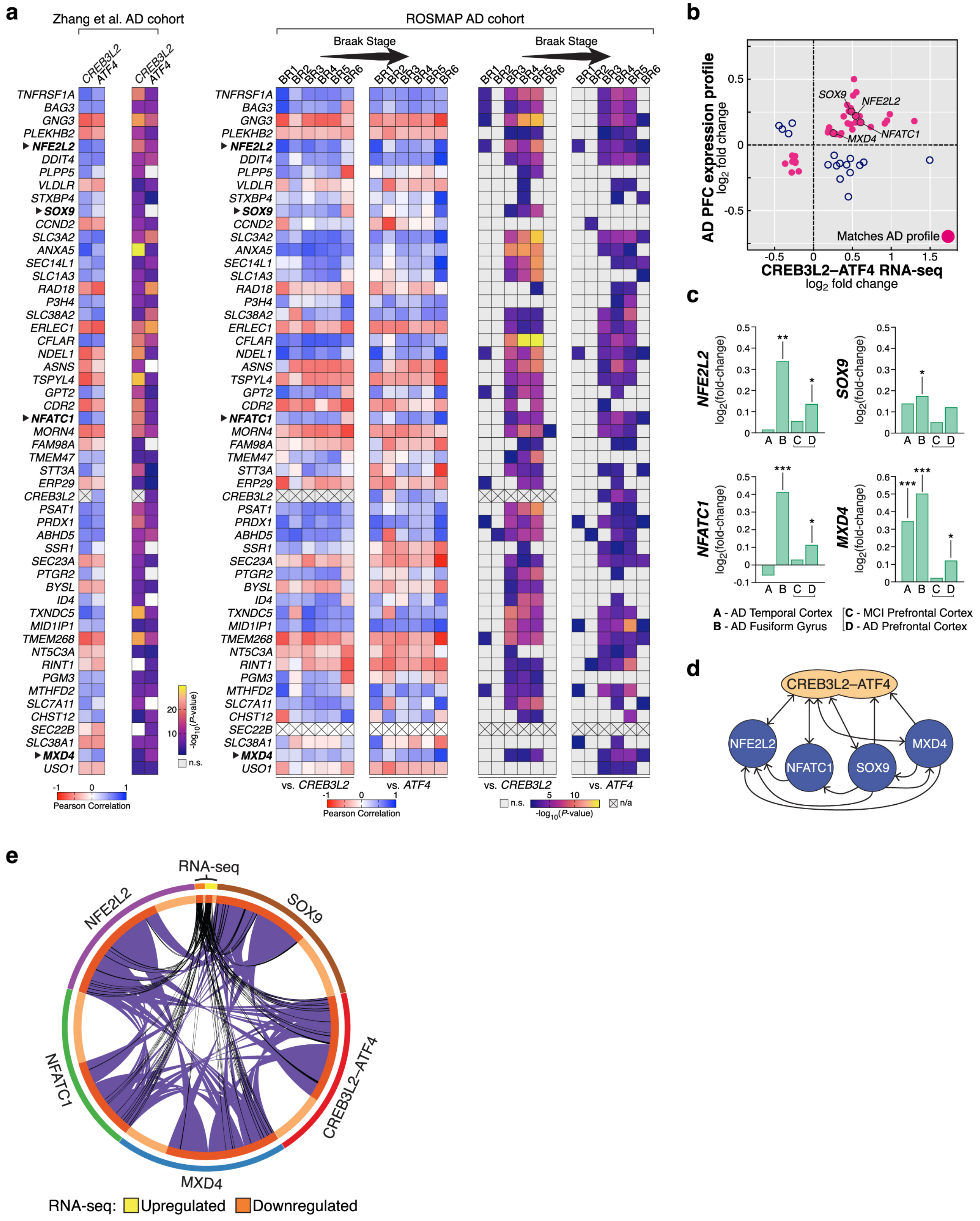
a
Zhang et al. AD cohort
ROSMAP AD cohort
Braak Stage
CREB3L2
ATF4
BR1 BR2 BR3 BR4 BR5 BR6
TNFRSF1A
BAG3
GNG3
PLEKHB2
NFE2L2
DDIT4
PLPP5
VLDLR
STXBP4
SOX9
CCND2
SLC3A2
ANXA5
SEC14L1
SLC1A3
RAD18
P3H4
SLC38A2
ERLEC1
CFLAR
NDEL1
ASNS
TSPYL4
GPT2
CDR2
NFATC1
MORN4
FAM98A
TMEM47
STT3A
ERP29
CREB3L2
PSAT1
PRDX1
ABHD5
SSR1
SEC23A
PTGR2
BYSL
ID4
TXNDC5
MID1IP1
TMEM268
NT5C3A
RINT1
PGM3
MTHFD2
SLC7A11
CHST12
SEC22B
SLC38A1
MXD4
USO1
-1 0 1
Pearson Correlation
20
10
-log10(P-value)
n.s.
vs. CREB3L2
vs. ATF4
5 10
n/a
b
AD PFC expression profile
log2 fold change
CREB3L2–ATF4 RNA-seq
log2 fold change
SOX9
NFE2L2
NFATC1
MXD4
Matches AD profile
0.5
0
-0.5
-0.5 0 0.5 1 1.5
c
NFE2L2
SOX9
NFATC1
MXD4
log2(fold-change)
A B C D
A - AD Temporal Cortex
B - AD Fusiform Gyrus
C - MCI Prefrontal Cortex
D - AD Prefrontal Cortex
d
CREB3L2–ATF4
NFE2L2
NFATC1
SOX9
MXD4
e
RNA-seq
NFE2L2
SOX9
NFATC1
CREB3L2–ATF4
MXD4
RNA-seq: Upregulated Downregulated

Fig. S5 – CREB3L2–ATF4 orchestrates AD-linked transcription network.

a. Expression correlation between CREB3L2–ATF4 targets with AD-associated transcriptional profiles and *CREB3L2* or *ATF4* in diseased individuals. These analyses were performed in two independent cohorts (ROSMAP, Religious Order Study/Memory and Aging Project) (*4, 49*). *P*-values indicate correlation significances. It is noteworthy that the number of cases assigned to each Braak stage varies considerably, with most individuals in the ROSMAP cohort being stratified between BR3 and BR5. n/a, not available (*SEC22B*) or not applicable (*CREB3L2*).

b. Head-to-head comparison of gene expression profiles in CREB3L2–ATF4 neurons and AD prefrontal cortex (*4*). These are the same 53 genes directly targeted by the heterodimer and misregulated in AD.

c. *NFE2L2*, *SOX9*, *NFATC1,* and *MXD4* AD gene expression profiles across various disease-relevant brain regions (*45, 46*). Prefrontal cortex samples shown here were mined from the ROSMAP cohort (*49*).

d. Regulatory interconnections within the wider CREB3L2–ATF4-activated NRF2-SOX9-NFATC1-MXD4 network.

e. Circos plot illustrating the regulatory relationships within the wider CREB3L2–ATF4 transcription network and their interaction with CREB3L2–ATF4-promoted transcriptional changes in neurons. Inner lines link genes shared by two datasets (e.g., SOX9 and NFATC1); black-colored lines additionally identify genes with altered transcriptional profiles downstream of CREB3L2–ATF4 activation in neurons.

Figure S6.

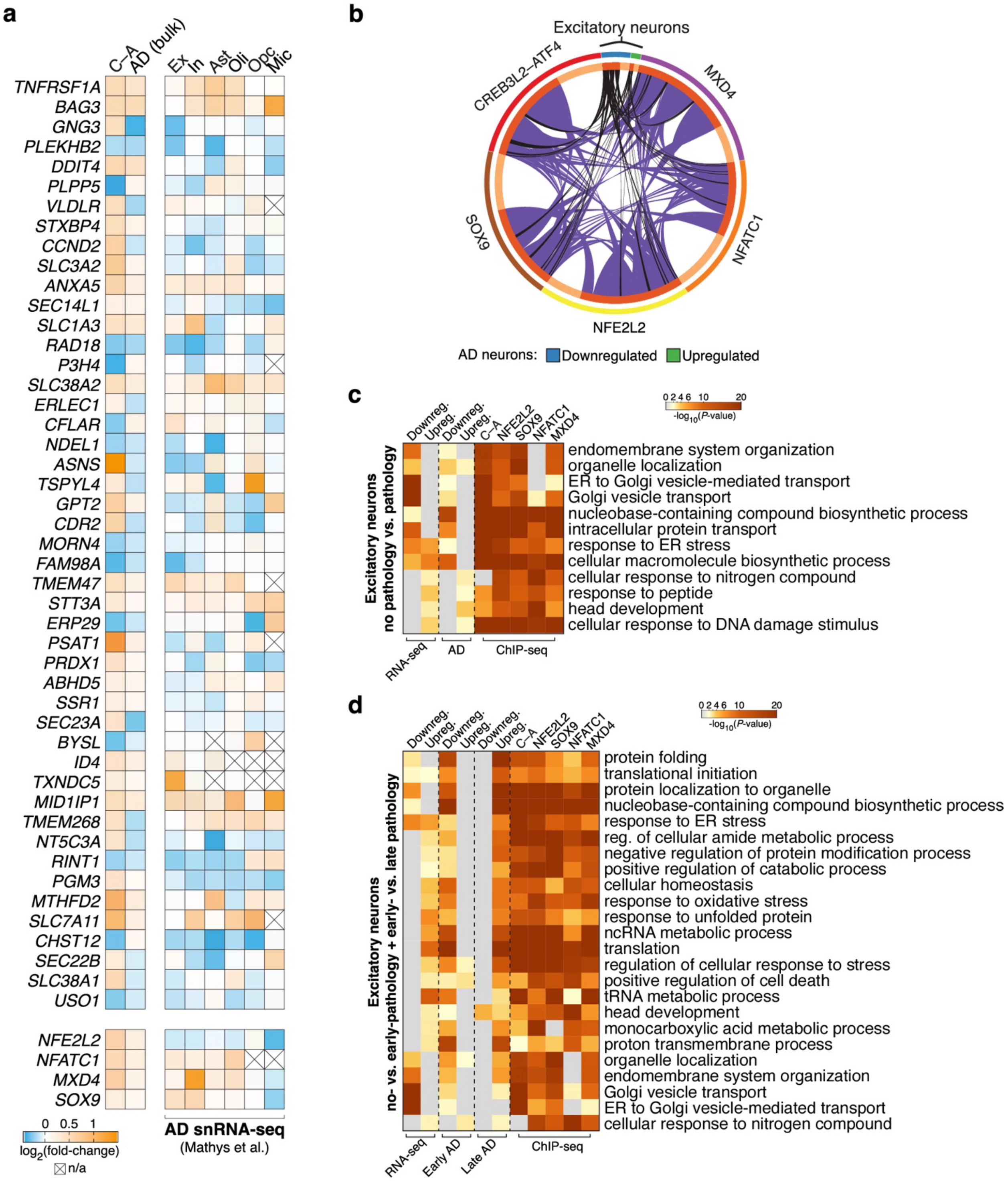

a
C–A
AD (bulk)
Ex
In
Ast
Oli
Opc
Mic
TNFRSF1A
BAG3
GNG3
PLEKHB2
DDIT4
PLPP5
VLDLR
STXBP4
CCND2
SLC3A2
ANXA5
SEC14L1
SLC1A3
RAD18
P3H4
SLC38A2
ERLEC1
CFLAR
NDEL1
ASNS
TSPYL4
GPT2
CDR2
MORN4
FAM98A
TMEM47
STT3A
ERP29
PSAT1
PRDX1
ABHD5
SSR1
SEC23A
BYSL
ID4
TXNDC5
MID1IP1
TMEM268
NT5C3A
RINT1
PGM3
MTHFD2
SLC7A11
CHST12
SEC22B
SLC38A1
USO1
NFE2L2
NFATC1
MXD4
SOX9
0 0.5 1
log2(fold-change)
n/a
AD snRNA-seq
(Mathys et al.)
b
Excitatory neurons
CREB3L2–ATF4
MXD4
SOX9
NFATC1
NFE2L2
AD neurons: Downregulated Upregulated
c
0 2 4 6 10 20
-log10(P-value)
Downreg.
Upreg.
Downreg.
Upreg.
C–A
NFE2L2
SOX9
NFATC1
MXD4
Excitatory neurons
no pathology vs. pathology
endomembrane system organization
organelle localization
ER to Golgi vesicle-mediated transport
Golgi vesicle transport
nucleobase-containing compound biosynthetic process
intracellular protein transport
response to ER stress
cellular macromolecule biosynthetic process
cellular response to nitrogen compound
response to peptide
head development
cellular response to DNA damage stimulus
RNA-seq
AD
ChIP-seq
d
0 2 4 6 10 20
-log10(P-value)
Downreg.
Upreg.
Downreg.
Upreg.
Downreg.
Upreg.
C–A
NFE2L2
SOX9
NFATC1
MXD4
Excitatory neurons
no- vs. early-pathology + early- vs. late pathology
protein folding
translational initiation
protein localization to organelle
nucleobase-containing compound biosynthetic process
response to ER stress
reg. of cellular amide metabolic process
negative regulation of protein modification process
positive regulation of catabolic process
cellular homeostasis
response to oxidative stress
response to unfolded protein
ncRNA metabolic process
translation
regulation of cellular response to stress
positive regulation of cell death
tRNA metabolic process
head development
monocarboxylic acid metabolic process
proton transmembrane process
organelle localization
endomembrane system organization
Golgi vesicle transport
ER to Golgi vesicle-mediated transport
cellular response to nitrogen compound
RNA-seq
Early AD
Late AD
ChIP-seq

Fig. S6 – CREB3L2–ATF4 overlaps with AD-relevant cellular dysfunctions in AD neurons.

a. Cell-type-specific transcriptomic analysis using snRNA-seq datasets produced by Mathys et al. (*7*). These are the same 53 DEGs examined in Fig. 4b. Controls, $n = 24$ (individuals with no or very low β-amyloid burden; AD-pathology, $n = 24$ (cases with high levels of β-amyloid and other neuropathological hallmarks of AD). Ex, excitatory neurons; In, inhibitory neurons; Ast, astrocytes; Oli, oligodendrocytes; Opc, oligodendrocyte precursor cells; Mic, microglia.

b. Regulatory relationships within the CREB3L2–ATF4 transcription network and their interaction with DEGs in AD excitatory neurons (*7*). Black-colored lines identify genes with AD-associated transcriptional profiles.

c-d. Representative GO terms enriched across input gene lists, colored by *P*-values (-$\log_{10}$-transformed). This comparative analysis integrates the DNA-binding program of each TF within the CREB3L2–ATF4 network, neuronal gene expression changes detected in AD excitatory neurons (*7*), and the transcriptional profile promoted by CREB3L2–ATF4 in cultured neurons described in Fig. 4a. In (d), progressive gene expression changes were considered by subgrouping individuals according to clinicopathological traits. ‘Early pathology’, as defined by Mathys and colleagues (*7*), included individuals with some amyloid burden but modest neurofibrillary tangles/cognitive impairment; cases presenting with higher amyloid loads, increased neurofibrillary tangles, and cognitive decline were classified as ‘late pathology’.

Figure S7.

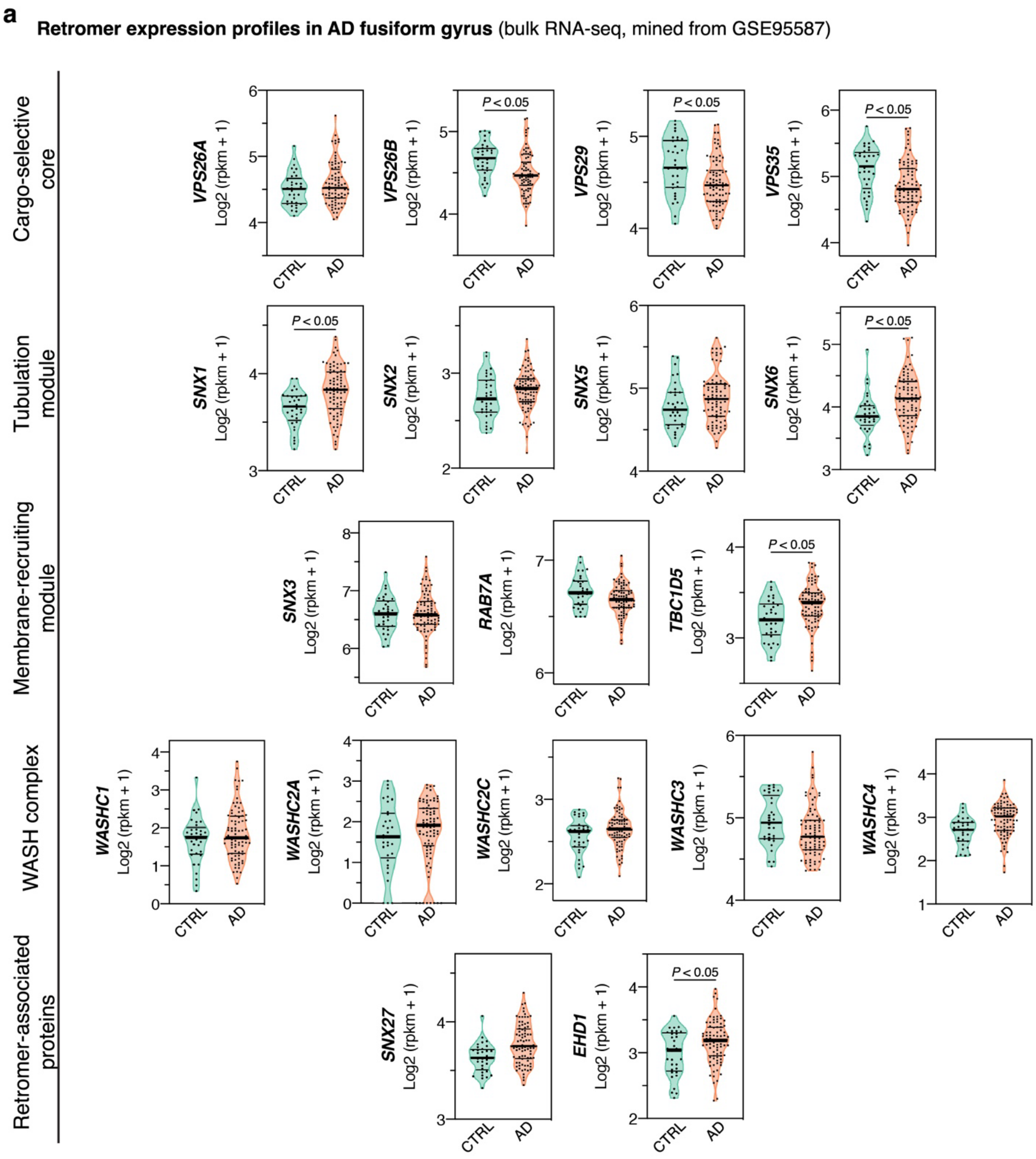
a
Retromer expression profiles in AD fusiform gyrus (bulk RNA-seq, mined from GSE95587)
Cargo-selective core
Tubulation module
Membrane-recruiting module
WASH complex
Retromer-associated proteins
VPS26A
VPS26B
VPS29
VPS35
SNX1
SNX2
SNX5
SNX6
SNX3
RAB7A
TBC1D5
WASHC1
WASHC2A
WASHC2C
WASHC3
WASHC4
SNX27
EHD1
Log2 (rpkm + 1)
P < 0.05
CTRL
AD

Fig. S7 – Retromer misregulation in AD brain (fusiform gyrus).

a. Differential expression analysis of retromer-associated genes in the fusiform gyrus. Datasets comparing control and AD cohorts were produced by Friedman et al. from bulk-extracted RNA (*46*). Controls, $n = 33$; AD cases, $n = 84$.

Figure S8.

a

Retromer expression profiles in AD temporal cortex (bulk RNA-seq, mined from GSE15222)

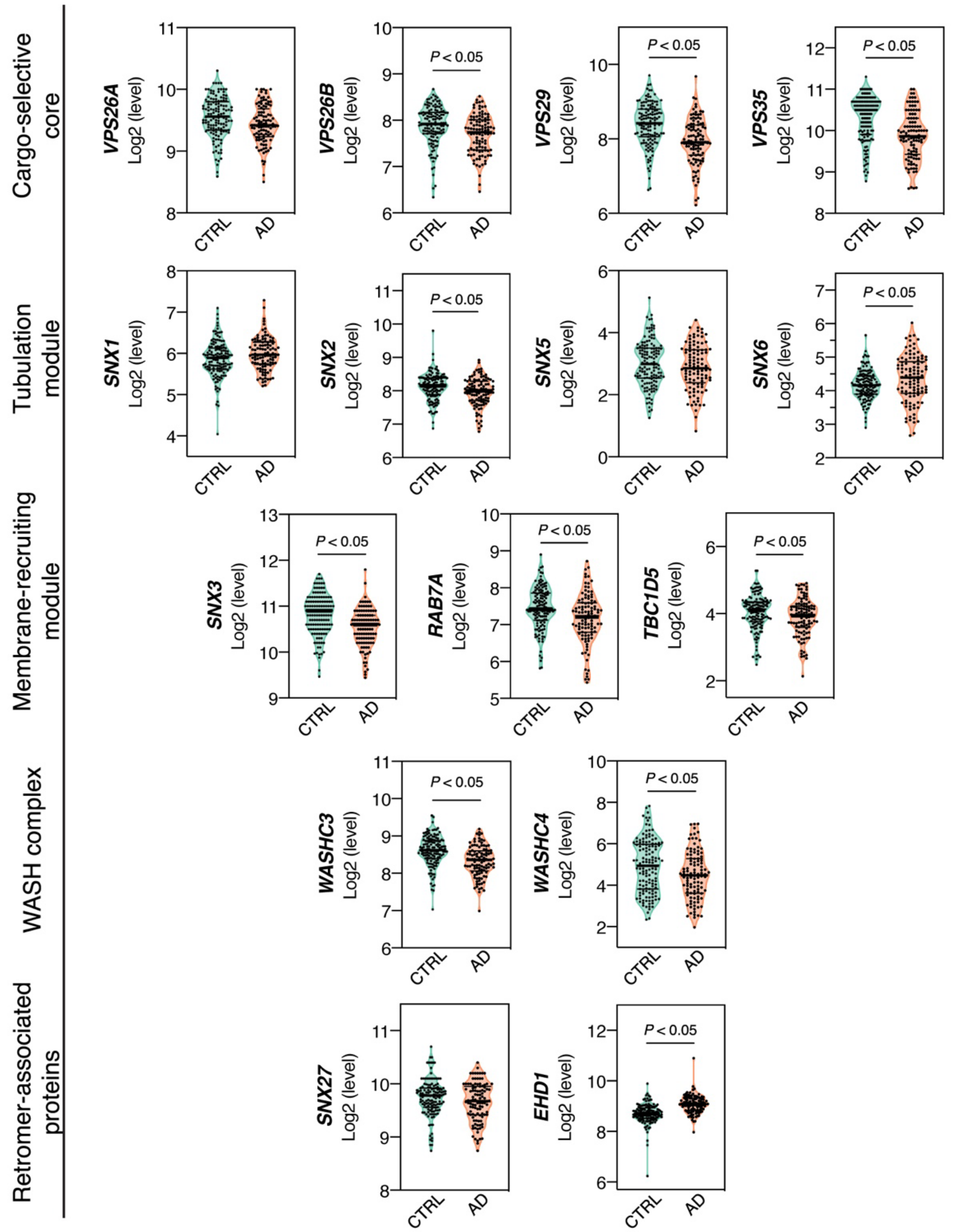

b

AD snRNA-seq: excitatory neurons, dorsolateral prefrontal cortex

(no pathology vs. pathology comparison, mined from Mathys et al.)

↓ Downregulated

Gene	IndModel Adj. *P*-value	IndModel fold-change	MixedModel z-score	MixedModel *P*-value	DEGs IndModel	DEGs MixedModel
↓ *SNX3*	1.30×10^{-93}	-0.22	-3.33	0.00087	FALSE	FALSE
↓ *VPS29*	8.07×10^{-81}	-0.28	-4.67	3.07×10^{-6}	TRUE	TRUE
↓ *RAB7A*	1.31×10^{-79}	-0.16	-3.84	0.00012	FALSE	FALSE
↓ *VPS35*	6.90×10^{-71}	-0.15	-1.67	0.096	FALSE	FALSE

Fig. S8 – Retromer misregulation in AD brain (bulk temporal cortex and snRNA-seq).

a. Differential expression analysis of retromer-associated genes in the temporal cortex. Datasets comparing control and AD cohorts were produced by Webster et al. from bulk-extracted RNA (*45*). Controls, n = 135; AD cases, n = 106.

b. Expression profiles for top-ranked retromer subunits in AD excitatory neurons derived from dorsolateral prefrontal cortex snRNA-seq datasets (*7*). DEGs IndModel: logical indication of whether a gene meets 'false discovery rate (FDR)-adjusted *P*-value' < 0.01 and 'absolute $\log_2$(foldchange)' > 0.25 conditions; DEGs MixedModel: logical indication of whether a gene meets 'FDR-adjusted MixedModel *P*-value' < 0.05 condition.

Figure S9.

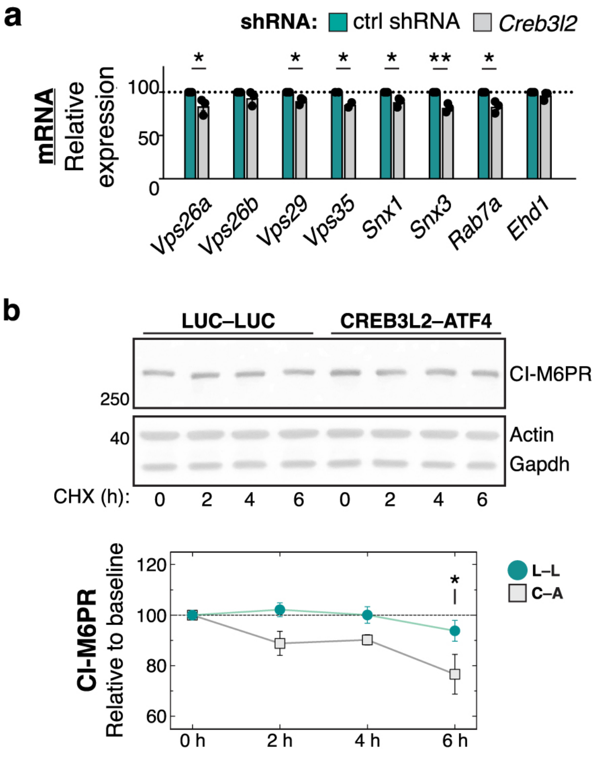
a
shRNA: ctrl shRNA Creb3l2
mRNA
Relative expression
100
50
0
*
*
*
*
**
*
Vps26a
Vps26b
Vps29
Vps35
Snx1
Snx3
Rab7a
Ehd1
b
LUC–LUC
CREB3L2–ATF4
CI-M6PR
250
40
Actin
Gapdh
CHX (h): 0 2 4 6 0 2 4 6
CI-M6PR
Relative to baseline
120
100
80
60
0 h
2 h
4 h
6 h
L–L
C–A

Fig. S9 – CREB3L2–ATF4 activation recapitulates AD retromer misregulation.

a. RT-qPCR analysis of retromer gene expression in *Creb3l2*-depleted rat cortical neurons. Measurements were normalized to *Tubb3* and *Pgk1* and are presented relative to control expression levels. Mean ± SEM of $n = 3$ independent experiments. *Vps26a*: **P*-value = 0.0402; *Vps29*: **P*-value = 0.0113; *Vps35*: **P*-value = 0.0316; *Snx1*: **P*-value = 0.0186; *Snx3*: ***P*-value = 0.0035; *Rab7a*: **P*-value = 0.0133; unpaired *t*-tests.

b. Western blot analysis of CI-M6PR degradation kinetics in control (LUC-LUC homodimer) and CREB3L2–ATF4-expressing hippocampal neurons after treatment with 40 µg/ml cycloheximide for 2, 4, or 6 hours. CI-M6PR levels are presented relative to $t = 0$ timepoint. Plot shows mean ± SEM of $n = 4$ independent experiments; two-way ANOVA with Sidak's multiple comparison test. Column factor (Background): **ANOVA *P*-value = 0.0014; Row factor (Time): **ANOVA *P*-value = 0.0078; *Sidak's *P*-value ($t = 6$ h) = 0.0203.

Figure S10.

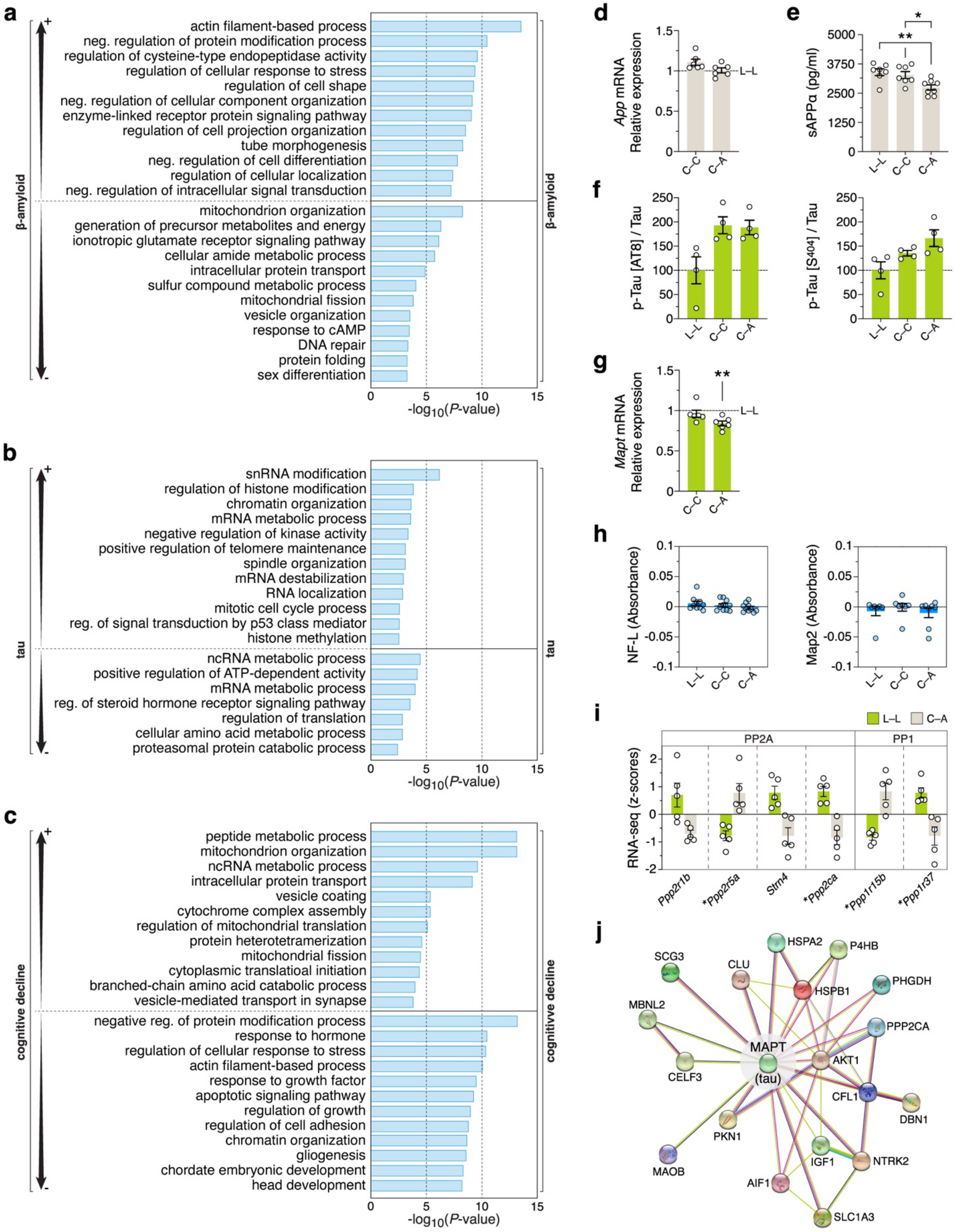
a
β-amyloid
actin filament-based process
neg. regulation of protein modification process
regulation of cysteine-type endopeptidase activity
regulation of cellular response to stress
regulation of cell shape
neg. regulation of cellular component organization
enzyme-linked receptor protein signaling pathway
regulation of cell projection organization
tube morphogenesis
neg. regulation of cell differentiation
regulation of cellular localization
neg. regulation of intracellular signal transduction
mitochondrion organization
generation of precursor metabolites and energy
ionotropic glutamate receptor signaling pathway
cellular amide metabolic process
intracellular protein transport
sulfur compound metabolic process
mitochondrial fission
vesicle organization
response to cAMP
DNA repair
protein folding
sex differentiation
-log10(P-value)
b
tau
snRNA modification
regulation of histone modification
chromatin organization
mRNA metabolic process
negative regulation of kinase activity
positive regulation of telomere maintenance
spindle organization
mRNA destabilization
RNA localization
mitotic cell cycle process
reg. of signal transduction by p53 class mediator
histone methylation
ncRNA metabolic process
positive regulation of ATP-dependent activity
mRNA metabolic process
reg. of steroid hormone receptor signaling pathway
regulation of translation
cellular amino acid metabolic process
proteasomal protein catabolic process
c
cognitive decline
peptide metabolic process
mitochondrion organization
ncRNA metabolic process
intracellular protein transport
vesicle coating
cytochrome complex assembly
regulation of mitochondrial translation
protein heterotetramerization
mitochondrial fission
cytoplasmic translatioal initiation
branched-chain amino acid catabolic process
vesicle-mediated transport in synapse
negative reg. of protein modification process
response to hormone
regulation of cellular response to stress
actin filament-based process
response to growth factor
apoptotic signaling pathway
regulation of growth
regulation of cell adhesion
chromatin organization
gliogenesis
chordate embryonic development
head development
cognitivve decline
d
App mRNA Relative expression
L–L
C–C
C–A
e
sAPPα (pg/ml)
f
p-Tau [AT8] / Tau
p-Tau [S404] / Tau
g
Mapt mRNA Relative expression
h
NF-L (Absorbance)
Map2 (Absorbance)
i
PP2A
PP1
RNA-seq (z-scores)
Ppp2r1b
*Ppp2r5a
Strn4
*Ppp2ca
*Ppp1r15b
*Ppp1r37
j
SCG3
CLU
HSPA2
P4HB
HSPB1
PHGDH
MBNL2
PPP2CA
MAPT
(tau)
AKT1
CELF3
CFL1
DBN1
PKN1
IGF1
NTRK2
MAOB
AIF1
SLC1A3

Fig. S10 –CREB3L2–ATF4 interacts with β-amyloid and tau neuropathologies.

a-c. Enriched GO terms for CREB3L2–ATF4-interacting genes positively and negatively associated ($-3 \leq$ signed $-\log_{10}$ *P*-value ≥ 3) with β-amyloid or tau neuropathologies. Pathology-interacting molecular networks were originally elucidated by De Jager and colleagues (*5*). Cognition, unlike β-amyloid and tau neuropathologies, declines over time; hence, a GO term negatively correlated with cognition indicates a deleterious association.

d. RT-qPCR analysis of *App* mRNA expression in CREB3L2–ATF4, CREB3L2–CREB3L2, and control neurons. Measurements were normalized to *Tubb3* (βIII-tubulin) and are presented relative to baseline levels (*Renilla* luciferase dimer background), indicated by the dashed line. Plot shows individual measurements and mean ± SEM of $n = 6$ independent experiments; one-way ANOVA test with Tukey's multiple comparison correction: *ANOVA *P*-value = 0.0308, *Tukey's *P*-value (C–C vs. L–L) = 0.0448.

e. Analysis of extracellular sAPPα levels by ELISA. Plots show individual measurements and mean ± SEM of n = 7 independent replicates; one-way ANOVA test with Tukey's multiple comparison correction. **ANOVA P-value = 0.0052, **Tukey's *P*-value (L–L vs. C–A) = 0.0066, *Tukey's *P*-value (C–C vs. C–A) = 0.0269.

f. Western blot quantification of tau phosphorylation in rat hippocampal neurons normalized against total tau levels. Plots show individual measurements and mean ± SEM of $n = 4$ independent replicates; sample differences are not statistically significant due to overall higher variability in control replicates. Measurements shown here pertain only to tau signals of 60 kDa isoform – see immunoblots in Fig. 6d.

g. RT-qPCR analysis of *Mapt* mRNA expression. Measurements were normalized to *Tubb3* (βIII-tubulin) and are presented relative to baseline levels (*Renilla* luciferase dimer background), indicated by the dashed line. Plot shows individual measurements and mean ± SEM of $n = 6$ independent experiments; one-way ANOVA test with Tukey's multiple comparison correction: *ANOVA *P*-value = 0.0059, **Tukey's *P*-value (C–A vs. L–L) = 0.0060, *Tukey's *P*-value (C–C vs. L–L) = 0.0351.

h. ELISA quantification of extracellular Neurofilament-light (NF-L) and MAP2 levels. Plots show individual measurements and mean ± SEM. NF-L: $n = 10$ independent replicates; MAP2: $n = 8$ independent replicates.

i. Differentially expressed PP2A and PP1 subunits after CREB3L2–ATF4 activation in primary hippocampal neurons, as determined by RNA-seq. *Ppp2r1b*: log fold-change = -0.40, *P*-value = 0.017; *Ppp2r5a*: log fold-change = +0.29, *P*-value = 0.014; *Strn4*: log fold-change = -0.17, *P*-value = 0.034; *Ppp2ca*: log fold-change = -0.15, *P*-value = 0.016; *Ppp1r15b*: log fold-change = +0.37, *P*-value = 0.00012; *Ppp1r37*: log fold-change = -0.20, *P*-value = 0.034. Asterisks denote DEGs found within the CREB3L2–ATF4 transcription network.

j. STRING protein interaction network of CREB3L2–ATF4-induced DEGs with links to tau (average local clustering coefficient = 0.787; network enrichment: *P*-value = 1.14×10^{-7}). Nodes represent proteins, and edges denote predicted functional associations. Purple edges represent experimentally determined protein-protein associations.

Figure S11.

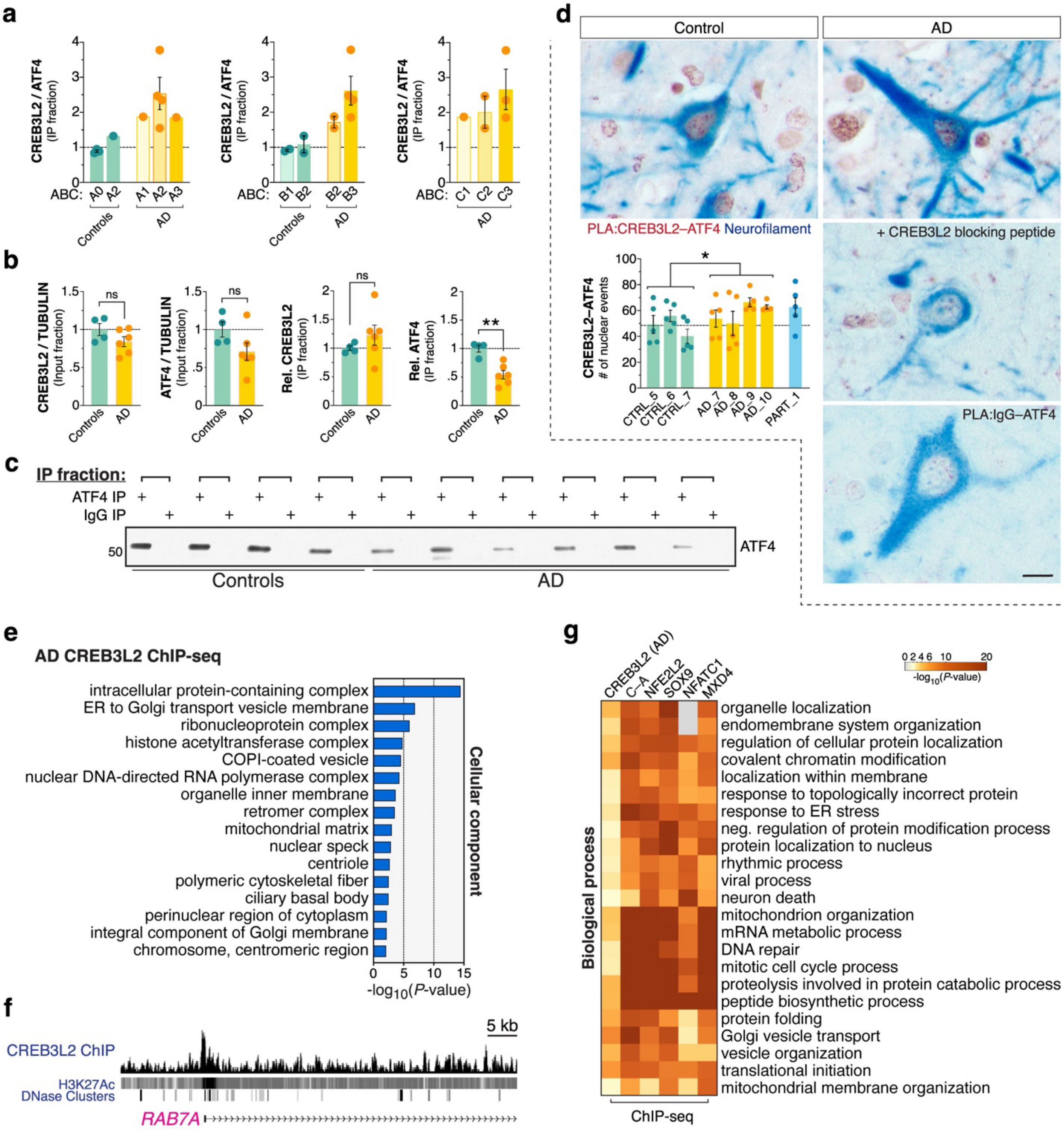

a
CREB3L2 / ATF4 (IP fraction)
ABC: A0 A2 A1 A2 A3
Controls
AD
ABC: B1 B2 B2 B3
ABC: C1 C2 C3
b
CREB3L2 / TUBULIN (Input fraction)
ATF4 / TUBULIN (Input fraction)
Rel. CREB3L2 (IP fraction)
Rel. ATF4 (IP fraction)
ns
**
c
IP fraction:
ATF4 IP
IgG IP
50
ATF4
Controls
AD
d
Control
AD
PLA:CREB3L2–ATF4 Neurofilament
+ CREB3L2 blocking peptide
PLA:IgG–ATF4
CREB3L2–ATF4 # of nuclear events
CTRL_5
CTRL_6
CTRL_7
AD_7
AD_8
AD_9
AD_10
PART_1
e
AD CREB3L2 ChIP-seq
intracellular protein-containing complex
ER to Golgi transport vesicle membrane
ribonucleoprotein complex
histone acetyltransferase complex
COPI-coated vesicle
nuclear DNA-directed RNA polymerase complex
organelle inner membrane
retromer complex
mitochondrial matrix
nuclear speck
centriole
polymeric cytoskeletal fiber
ciliary basal body
perinuclear region of cytoplasm
integral component of Golgi membrane
chromosome, centromeric region
Cellular component
-log10(P-value)
f
5 kb
CREB3L2 ChIP
H3K27Ac
DNase Clusters
RAB7A
g
CREB3L2 (AD)
C–A
NFE2L2
SOX9
NFATC1
MXD4
-log10(P-value)
organelle localization
endomembrane system organization
regulation of cellular protein localization
covalent chromatin modification
localization within membrane
response to topologically incorrect protein
response to ER stress
neg. regulation of protein modification process
protein localization to nucleus
rhythmic process
viral process
neuron death
mitochondrion organization
mRNA metabolic process
DNA repair
mitotic cell cycle process
proteolysis involved in protein catabolic process
peptide biosynthetic process
protein folding
Golgi vesicle transport
vesicle organization
translational initiation
mitochondrial membrane organization
Biological process
ChIP-seq

Fig. S11 – CREB3L2–ATF4 heterodimers are present in AD brain.

a. CREB3L2–ATF4 co-immunoprecipitation dataset shown in Fig. 7a stratified according to ABC scores (A, amyloid; B, Braak; C, CERAD [neuritic plaques]). Plots show individual measurements and mean ± SEM of CREB3L2/ATF4 ratios from $n = 4$ controls and $n = 6$ AD cases.

b. Additional quantifications of co-immunoprecipitation reactions of CREB3L2–ATF4 heterodimers in control and late-onset AD prefrontal cortex shown in Fig. 7a. Plots show mean ± SEM of $n = 4$ controls and $n = 6$ AD cases; unpaired *t*-tests were performed for statistical comparisons; ***P*-value = 0.0022. Decreased overall levels of ATF4 may indicate prolonged CREB3L2–ATF4 activation (see, for example, fig. 4a, fig. 6c, and fig. S3d).

c. Immunoprecipitation reaction with control rabbit IgG in control and AD prefrontal cortex shows no enrichment of ATF4. These are the same cases analyzed in Fig. 7a and fig. S11a,b.

d. Quantification of dorsolateral prefrontal CREB3L2–ATF4 heterodimers (red punctate signals) in control and AD cases. Brains were co-stained for neurofilament (blue labelling), a neuronal marker. PART, primary age-related tauopathy. Plots show, for each case, average neuronal CREB3L2–ATF4 signals across non-contiguous layer III-V segments (mean ± SEM). Controls, $n = 3$; AD, $n = 4$; PART, $n = 1$; **P*-value = 0.0234, unpaired *t*-test. Technical control #1: CREB3L2 PLA probe and CREB3L2 blocking peptide were co-incubated before proceeding with the assay; Technical control #2: CREB3L2 PLA probe was substituted with rabbit IgG PLA probe. Scale bar, 5 µm.

e. GO term enrichment analysis of AD CREB3L2 targets (cellular component).

f. AD CREB3L2 ChIP-seq genome browser tracks in *RAB7A* locus juxtaposed with ENCODE-produced H3K27Ac and DNaseI hypersensitivity profiles. For H3K27Ac and DNaseI hypersensitivity profiles, signal strength is indicated by increasingly darker shades.

g. Representative GO terms enriched across input gene lists, colored by *P*-values ($-\log_{10}$-transformed). This comparative analysis integrates the DNA-binding program of each TF within the CREB3L2–ATF4-activated NRF2-SOX9-NFATC1-MXD4 transcription network, as well as the AD CREB3L2 ChIP-seq readout.

Table S1.

Table S1. Neuropathological evaluation (co-immunoprecipitation)

Classification	Sample ID	Age	Sex	Cold PMI[a]	Frozen PMI[b]	A*	B*	C*
Control	CTRL_1	54	Female	6:41	16:36	A0	B2	Not eligible
Control	CTRL_2	62	Male		5:24	A0	B1	Not eligible
Control	CTRL_3	67	Male	11:10	15:10	A0	B1	Not eligible
Control	CTRL_4	89+	Male	4:47	11:17	A2	B2	C1
AD	ALZ_1	89+	Male		11:26	A2	B3	C2
AD	ALZ_2	68	Male	1:00	13:30	A2	B3	C3
AD	ALZ_3	63	Male	3:10	12:10	A2	B3	C3
AD	ALZ_4	63	Male		22:24	A3	B3	C3
AD	ALZ_5	89+	Female	4:30	6:20	A1	B2	C1
AD	ALZ_6	89+	Male	4:00	27:30	A2	B2	C2

[a]Cold PMI: Postmortem interval calculated from the reported time of death to the time the patient was brought into the cold room.

[b]Frozen PMI: Postmortem interval calculated from the reported time of death to the mean time the brain was processed.

*Neuropathological classification follows ABC criteria.

Cases are listed in the same order used for loading co-immunoprecipitation gels in Fig. 7a.

Table S2.

Table S2. Neuropathological evaluation (proximity ligation assay)

Classification	Sample ID	Age	Sex	Frozen PMI[b]	Clinical diagnosis	Neuropathological diagnosis	A*	B*	C*
Control	CTRL_5	67	Female	20.7	Mesenteric ischemia	No diagnostic abnormality			
Control	CTRL_6	53	Female	15.4	Vasculopathy	Hemorrhagic infarct, remote/chronic, cerebellum			
Control	CTRL_7	38	Female	23.7	Cirrhosis of the liver (ethanol)	Cerebellar vermian atrophy, severe			
PART	PART_1	68	Male	22	Peritoneal bleeding	Primary age-related tauopathy	A0	B1	C0
AD	ALZ_7	83	Female	18	Advanced dementia	AD neuropathologic changes, intermediate	A3	B2	C2
AD	ALZ_8	82	Male	36.7	Alzheimer's & COVID-19	AD neuropathologic changes, intermediate	A2	B2	C2
AD	ALZ_9	90	Female	8.9	Alzheimer's & COVID-19	AD neuropathologic changes, moderate	A2	B3	C2
AD	ALZ_10	74	Male	11.8	Dementia (Alzheimer's type), seizures, anxiety	AD neuropathologic changes, severe	A3	B3	C3

[a]Frozen PMI (hours): Postmortem interval calculated from the reported time of death to the mean time the brain was processed.

*Neuropathological classification follows ABC criteria.

Table S3.

Table S3. Neuropathological evaluation (ChIP-seq)

Classification	Sample	Age	Sex	Cold PMI[a]	Frozen PMI[b]	NIA-Reagan consensus criteria	Braak NFT Stage	CERAD Plaque Score
AD	ALZ_11	89+	Female	0:45	16:30	High	V	C
AD	ALZ_12	89+	Female	1:25	20:10	Intermediate	V	A

[a]Cold PMI: Postmortem interval calculated from the reported time of death to the time the patient was brought into the cold room.

[b]Frozen PMI: Postmortem interval calculated from the reported time of death to the mean time the brain was processed.

Table S4.

Table S4. List of primers used for ChIP-qPCR

Target gene	Primer sequence (5' to 3' direction)
Chop	ACTTCCGGGTCCGAGATAAC<br>GTGTCCAGGAGCCTACCAATC
Vps26a	GAGGAAGCAAGGATTTGTGC<br>GTGAGATCAGGTGCGAAGGT
Vps26b	CGCACTCACTGAACTGCCTA<br>GTGGAGAGGGAGAAGACGTG
Vps29	CAATGAGACGACGAGTTTGC<br>GAGGAATTTCTCGCAGCAC
Vps35	GACTTTATGTGGGCCAATCG<br>ACAAGCAGCAGCGCCTAC
Snx1	GGCGCCAGTGAAAATATCCT<br>GGGAGGTGGTGGCTGTAG
Snx3	TCACAGTGAGGCACTGGACT<br>ACCCCGGAAATGATTTTAGC
Rab7a	CACCATATTGGGCCAAGAAC<br>GTTCCAAAGGGGGACACTCT
Ehd1	GTCTGTACGCCGGTCCTTG<br>GGAGACAGAGCTGGCTGCTA

Table S5.

Table S5. TaqMan probes for RT-qPCR analysis of gene expression

Target gene	Species	Assay ID
Creb3l2	*Rattus norvegicus*	Rn01455999_m1
Atf4	*Rattus norvegicus*	Rn00824644_g1
Vps26a	*Rattus norvegicus*	Rn01433541_m1
Vps26b	*Rattus norvegicus*	Rn02111368_s1
Vps29	*Rattus norvegicus*	Rn01480547_m1
Vps29	*Rattus norvegicus*	Rn01480546_m1
Vps35	*Rattus norvegicus*	Rn01538117_m1
Vps35	*Rattus norvegicus*	Rn01538113_m1
Snx1	*Rattus norvegicus*	Rn01418446_m1
Snx3	*Rattus norvegicus*	Rn01441118_m1
Rab7a	*Rattus norvegicus*	Rn00576640_m1
Ehd1	*Rattus norvegicus*	Rn06302200_s1
App	*Rattus norvegicus*	Rn00570673_m1
Mapt	*Rattus norvegicus*	Rn00691532_m1
Tubb3	*Rattus norvegicus*	Rn01431594_m1
Pgk1	*Rattus norvegicus*	Rn00821429_g1
Gapdh	*Rattus norvegicus*	Rn01775763_g1

Table S6.

Table S6. Breakdown of statistical comparisons.

Figure	Detailed Statistical Analyses
Fig. 5e	Vps26: ***P*-value = 0.0063; Vps29: ***P*-value = 0.0062; Vps35: ***P*-value = 0.0060; Snx1: ***P*-value = 0.0099; Snx3: ****P*-value = 0.0001; Rab7a: **P*-value = 0.0159; unpaired *t*-tests.
Fig. 5f	*Vps26b*: **P*-value = 0.0209; *Vps29*: ***P*-value = 0.0070; *Vps35*: ***P*-value = 0.0085; *Snx1*: ***P*-value = 0.0082; *Snx3*: ****P*-value = 0.0004; *Rab7a*: ****P*-value = 0.0009; unpaired *t*-tests.
Fig. 5g	*Vps26b*: **ANOVA *P*-value = 0.0042, **Tukey's *P*-value (C–C vs. C–A) = 0.0032. *Vps35*: **ANOVA *P*-value = 0.0042, **Tukey's *P*-value (L–L vs. C–C) = 0.0040, *Tukey's *P*-value (C–C vs. C–A) = 0.0325. *Ehd1*: ***ANOVA *P*-value = 0.0005, **Tukey's *P*-value (L–L vs. C–C) = 0.0005, *Tukey's *P*-value (C–C vs. C–A) = 0.0044.
Fig. 5h	Vps29: *ANOVA *P*-value = 0.0254, *Sidak's *P*-value (L–L vs. C–A) = 0.0455, *Sidak's *P*-value (C–C vs. C–A) = 0.0256. Vps35: *ANOVA *P*-value = 0.0321, *Sidak's *P*-value (C–C vs. C–A) = 0.0327. Snx3: *Sidak's *P*-value (L–L vs. C–A) = 0.0434. Rab7a: *ANOVA *P*-value = 0.0165, *Sidak's *P*-value (C–C vs. C–A) = 0.0298. Ehd1: *ANOVA *P*-value = 0.0199, *Sidak's *P*-value (L–L vs. C–C) = 0.0195.
Fig. 6b	$A\beta_{42}/A\beta_{40}$: ***ANOVA *P*-value = 0.0007, **Tukey's *P*-value (L–L vs. C–A) = 0.0018, **Tukey's *P*-value (C–C vs. C–A) = 0.0022. $A\beta_{42}$: ***ANOVA *P*-value = 0.0003, ***Tukey's *P*-value (L–L vs. C–A) = 0.0003, **Tukey's *P*-value (C–C vs. C–A) = 0.0094. $A\beta_{40}$: ***ANOVA *P*-value = 0.0003, ***Tukey's *P*-value (L–L vs. C–A) = 0.0003, **Tukey's *P*-value (C–C vs. C–A) = 0.0069.
Fig. 6d	AT8: *ANOVA *P*-value = 0.0271, *Tukey's *P*-value (L–L vs. C–A) = 0.0474. PHF-1: *ANOVA *P*-value = 0.0162, **Tukey's *P*-value (L–L vs. C–A) = 0.0076. p-Ser404: **ANOVA *P*-value = 0.0086, *Tukey's *P*-value (L–L vs. C–A) = 0.0333.
Fig. 6e	**ANOVA *P*-value = 0.0095, *Tukey's *P*-value (L–L vs. C–A) = 0.0109, *Tukey's *P*-value (C–C vs. C–A) = 0.0486.

Supplementary Materials & Methods

CHOP immunocytochemistry and image analysis

Neurons grown in microfluidic chambers were fixed for 20 minutes at room temperature with a PBS-based 4% paraformaldehyde, 4% sucrose solution and thoroughly washed with PBS. Permeabilization and blocking were performed with 3 mg ml^{-1} bovine serum albumin, 100 mM glycine, and 0.25% Triton X-100 in PBS. Samples were incubated overnight with a primary antibody against CHOP (1:1,000; #2895, Cell Signaling Technology) diluted in permeabilization/blocking buffer. Following multiple PBS washes, a fluorophore-conjugated Alexa secondary antibody (1:1,000, Thermo Fisher) was applied for 1 hour. Samples were preserved in ProLong Diamond Antifade (Thermo Fisher) and imaged using an Axio-Observer.Z1 microscope equipped with an EC Plan-Neofluar 40×/1.3 objective and an AxioCam MRm Rev. 3 camera (Zeiss). Image acquisition settings were calibrated to avoid pixel saturation using AxioVision software (Zeiss). Acquisition parameters were kept constant between samples in any given experiment. Mean pixel intensity values were computed after background fluorescence subtraction.

Chromatin immunoprecipitation and quantitative PCR (ChIP-qPCR)

For each immunoprecipitation/condition, approximately 15×10^6 rat cortical neurons obtained from E16 Sprague-Dawley embryos were cultured for a week on 0.1 mg ml^{-1} poly-D-lysine (Millipore Sigma), 2 μg ml^{-1} laminin (Bio-Techne) pre-coated flasks and maintained in Neurobasal medium supplemented with B27 and L-glutamine; $A\beta_{42}$ oligomers, prepared as described above, were bath-applied 36 hours prior to immunoprecipitation protocol. Protein-DNA cross-links were promoted using formaldehyde at a final concentration of 1% (v/v), and the cross-linking reaction allowed to proceed for 10 minutes at room temperature, as per manufacturer's instructions (SimpleChIP Plus Kit [#9005], Cell Signaling Technology). Chromatin fragments (150-900 base pair-long) were obtained by partial digestion with micrococcal nuclease (MNase) incubated for 11 minutes at 37ºC in a Thermomixer R (Eppendorf) with frequent mix cycles. Nuclear membranes were broken up by three rounds of 20-second pulses on a low setting (15% amplitude) using a Sonic Dismembrator (Model 500, Fisher Scientific) and lysates subsequently clarified by centrifugation. Adequate digestion was confirmed by agarose gel electrophoresis. Per immunoprecipitation/condition, 10 μg of digested, cross-linked chromatin was used. Antibodies were incubated overnight at 4ºC with end-over-end rotation: anti-CREB3L2 (2 μg; HPA015068, Atlas Antibodies) or, as negative control, rabbit normal serum IgG (#2729). Immunoprecipitates were captured using Protein G magnetic beads (#9006, Cell Signaling Technology) and washed with low- and high-salt buffers. Elution was performed at 65ºC and 1,200 rpm for 30 minutes using a thermomixer. Protein-DNA cross-links were then reversed by treatment with Proteinase K for 2 hours at 65ºC, and DNA column-purified. ChIP signals were measured by quantitative PCR with QuantiTect SYBR Green PCR master mixes from Qiagen (Table S4). CREB3L2 enrichment was computed using the percent input method, whereby ChIP signals are normalized to input.

Transwell neuronal culture and co-immunoprecipitation

Transwell inserts (1 μm pore size; Millipore [MCRP06H48]) were mounted on 6-well plates and both sides of the membrane sequentially coated with 0.1 mg ml^{-1} poly-D-lysine (Millipore Sigma) and 2 μg ml^{-1} laminin (Bio-Techne). A mix of E18 rat cortical and hippocampal neurons (800,000 per well) was plated on the bottom side of the insert (flipped up during this part of the protocol) and allowed to settle for 20-30 minutes. Transwell inserts were then carefully placed back in the plates, now reverted to their original orientation. With this set-up, neurites grow upward towards the top half of the membrane, rendering them more accessible to treatment. Cells were maintained for 10-12 DIV in Neurobasal containing 1× B27 and 2 mM L-glutamine, after the first 24 hours on Neurobasal supplemented with 10% fetal bovine serum, 2 mM L-glutamine, 1 mM sodium

pyruvate, and antibiotics (50 U ml^{-1} penicillin-streptomycin). Following a PBS wash (ice-cold), cell bodies were gently scraped off with a cotton swab and neuritic fractions (comprised of at least two pooled transwells, ca. 200 µg of protein) collected in CHAPS buffer (150 mM KCl, 50 mM HEPES, 0.1% CHAPS, adjusted to pH 7.4, plus protease inhibitors). Co-immunoprecipitations were performed overnight at 4ºC with rotation using antibody-bound M-280 Dynabeads (Thermo Fisher); antibodies: mouse anti-CREB3L2 (1:200; MABE1018, Millipore) or, as negative control, normal mouse IgG serum. Finally, beads were washed five times with PBS, resuspended in 25 µl of Laemmli buffer containing 5% β-mercaptoethanol, and boiled before western blot analysis.

Preparation of α-synuclein pre-formed fibrils

Recombinant human α-synuclein monomers (#RP-003, Proteos) were used to generate pre-formed fibrils as previously described (*22*). Briefly, monomers were diluted to a concentration of 5 mg/mL in PBS and shaken at 1000 rpm in an Eppendorf Thermomixer C for 7 days at 37°C to generate fibrils. Fibrils were aliquoted and stored at -80°C. Prior to treatment of hippocampal neurons, aliquots were thawed at room temperature, diluted to a concentration of 0.1 µg/µL in PBS, and sonicated in a QSonica 700 sonicator with cup horn at 30% amplitude for a total of 22.5 minutes (3 seconds on, 2 seconds off). Sonicated fibrils were added to hippocampal neurons on DIV 4 at a concentration of 5 mg/mL for 10 days. Intraneuronal pathology was confirmed by staining against phospho-α-synuclein ($pSer^{129}$; 1:250; #23706, Cell Signaling Technology).

RNA-sequencing

Prior to RNA collection, neuronal cultures were washed with ice-cold HBSS (Thermo Fischer) and lysed with TRIzol reagent (#15596026, Thermo Fisher) by scraping. The reaction was allowed to proceed on ice for 5 minutes, at which point samples were centrifuged at 12,000 *g* and 4ºC and supernatants transferred to new tubes before a 1:1 (v/v) dilution with molecular biology-grade 100% ethanol (Millipore Sigma). The RNA extraction protocol was continued using the Direct-zol RNA MicroPrep kit (Zymo Research), in accordance to manufacturer's instructions, and included an on-column DNA digestion step with DNase I. RNA was eluted in 30 µl of water. RNA-seq library preparation and sequencing reactions were conducted at GENEWIZ, Inc. (South Plainfield, NJ, USA). RNA sequencing libraries were prepared using the NEBNext Ultra RNA Library Prep Kit for Illumina following manufacturer's instructions (NEB). The samples were sequenced using a 2x150bp Paired-End (PE) configuration. Image analysis and base calling were conducted by HiSeq Control Software (HCS). Raw sequence data (.bcl files) generated from Illumina HiSeq was converted into fastq files and de-multiplexed using Illumina's bcl2fastq 2.17 software. One mismatch was allowed for index sequence identification. RNA-seq sequencing data were processed and analyzed within the Galaxy web platform, using the public server at usegalaxy.org. First, library adapters and low-quality reads were removed using Trimmomatic (version 0.38) with the following settings: initial ILLUMINACLIP step to cut adapters and other Illumina-specific sequences from the reads, seedMismatches = 2, palindromeClipThreshold = 30, simpleClipThreshold = 10, minAdapterLength = 8, keepBothReads = True, AVGQUAL ≥ 20, SLIDINGWINDOW: windowSize = 4 and requiredQuality = 20, and MINLEN = 50. Second, reads were mapped to the Rattus norvegicus (Rn) 6.0 reference genome and gene model (downloaded from Ensembl) with RNA STAR (version 2.7.5b) using default settings except: sjdbOverhang = 149, outFilterType = True, alignIntronMax = 1000000, alignMatesGapMax = 1000000, and alignSJoverhangMin = 8; additionally, unmapped reads, alignments that had junctions with inconsistent strands, alignments across unannotated non-canonical junctions and all alignments across non-canonical junctions were excluded from output. Third, featureCounts (version 1.6.4+galaxy2) was run to quantify reads mapping to exons with the following parameters: 'create gene length file' = True, 'count fragments instead of reads' = True, 'only allow fragments with both reads aligned' = True, 'exclude chimeric fragments' = True, 'GFF feature type filter' = exon, 'GFF

gene identifier' = gene_id, 'on feature level' = False, 'allow reads to map to multiple features' = False, 'minimum mapping quality per read' = 10; otherwise default tool settings. Forth, differentially expressed features were determined using default DESeq2 (version 2.11.40.6+galaxy1) settings, except: 'output normalized counts table' = True. Fifth, DESeq2 output was filtered to extract only the most differentially expressed genes (adjusted *P*-value < 0.05) between conditions. Sixth, the Galaxy tool 'Annotate DESeq2/DEXSeq output tables' (version 1.1.0) was employed to retrieve gene annotations. Subsequently, to visualize gene expression profiles over samples, Z-scores were computed from normalized gene counts and plotted using the heatmap2 tool. Comparative gene ontology enrichment meta-analyses were performed with differential expression lists through the Metascape web portal (*36*).

shRNA preparation and delivery

shERWOOD-UltramiR shRNAs targeting *R. norvegicus Creb3l2* (TLRSU1400-362339) were acquired from transOMIC Technologies (Huntsville, Alabama, USA). For expression in primary neuronal cells, the original CMV promoter was substituted with that of the human ubiquitin C (*UBC*) gene using the ClaI and AgeI sites. shRNA lentiviral particles were produced in HEK293T cells and titers measured with qPCR Lentivirus Titration Kit (Applied Biological Materials). Briefly, envelope, packaging, and shRNA-carrying lentiviral vectors (3:7:10 ratio) were delivered using Lipofectamine 3000; 6 hours post-transfection, the medium was changed to Neurobasal containing B27 and L-glutamine. Viral supernatant was collected after 36 hours, passed through a 0.45 µm PES filter, aliquoted, and stored at -80ºC.

RNA extraction and quantitative real-time PCR

At the time of collection, cells were washed with ice-cold Hank's Balanced Salt Solution (HBSS; Thermo Fischer) and lysed with TRIzol reagent (#15596026, Thermo Fisher) by scraping. The reaction was allowed to proceed on ice for 5 minutes, at which point samples were centrifuged at 12,000 *g* and 4ºC and supernatants transferred to new tubes before a 1:1 (v/v) dilution with molecular biology-grade 100% ethanol (Millipore Sigma). The RNA extraction protocol was continued using the Direct-zol RNA MicroPrep kit (Zymo Research), in accordance to manufacturer's instructions, and included an on-column DNA digestion step with DNase I. RNA was typically eluted in 50 µl of water. Real-time quantification of target RNA species was performed in triplicate on a StepOnePlus Real-Time qPCR system using the Luna Universal One-Step RT-qPCR kit (New England Biolabs); reverse transcription and PCR amplification were primed with TaqMan hydrolysis probes (Table S5; Thermo Fisher). The ΔΔCt method was employed for obtaining relative gene expression data points; reference gene stability across conditions was tested in preliminary experiments using the geNorm module in qbase+ (Biogazelle). Axonal or somatic total RNA was similarly isolated by TRIzol extraction coupled with Direct-zol RNA MicroPrep kit purification from 25 microfluidic chambers per condition, reverse transcribed (SuperScript III, Thermo Fisher), and cDNA preamplified for 20 cycles using the TaqMan PreAmp Master Mix kit (Thermo Fisher), according to manufacturer's instructions. Real-time PCR quantification was performed on a StepOnePlus system using the TaqMan Gene Expression Master Mix. Serial dilution calibration curves were calculated to assess overall sample quality and amplification efficiency. Ct values were interpolated from these curves and expression levels normalized to input RNA.

CI-M6PR cycloheximide chase

Control and CREB3L2–ATF4-expressing rat hippocampal neurons, supplemented every 2 days with 100 nM heterodimerizer, were grown until DIV10 before treatment with cycloheximide (40 µg/ml, #C4859, Millipore Sigma) for 2-6 hours, as previously described (*47*). Cells were lysed in 2× Laemmli buffer (130 mM Tris-Cl pH 6.8, 0.1 mM dithiothreitol, 20% (v/v) glycerol and 4% sodium dodecyl sulfate diluted in water) by

scraping, boiled at 85ºC for 5 minutes, and analyzed by western blot using an antibody against CI-M6PR (1:30,000; ab124767, Abcam).

CREB3L2 ChIP-sequencing in AD prefrontal cortex

Autopsy cases #ALZ_11 and #ALZ_12 (Table S3), both females with moderate AD pathology, were chosen for ChIP-seq analysis based on 1) high CREB3L2 and ATF4 expression level, 2) CREB3L2–ATF4 complex accumulation and 3) reduced postmortem processing intervals. Frozen minced brain tissue (approximately 150 mg per immunoprecipitation and a total of 300 mg per case) was transferred to a conical tube containing 6 ml of PBS supplemented with protease inhibitors (PBS +PI), and protein-DNA cross-linking allowed to develop for 20 minutes at room temperature using formaldehyde at a final concentration of 1.5% (v/v). Cross-linking reaction was then quenched with glycine (5 minutes at room temperature), as per manufacturer's instructions (SimpleChIP Plus Kit [#9005], Cell Signaling Technology). After rinsing in ice-cold PBS + PI, we proceeded by disaggregating tissue using an ice-cold Dounce homogenizer (7 ml total capacity) until a single-cell suspension was obtained, which was followed by a 2,000 *g* and 4ºC centrifugation step. The resulting supernatants were discarded. Chromatin fragments (mainly 1-3 nucleosomes in size) were obtained by partial digestion with micrococcal nuclease (MNase; 2 µl in 500 µl) incubated for 13 minutes at 37ºC in a Thermomixer R (Eppendorf) programmed for frequent mix cycles. Nuclear membranes were broken up by three rounds of 20-second, 15% amplitude pulses using a Sonic Dismembrator Model 500 (Fisher Scientific), and lysates subsequently clarified by centrifugation. Adequate digestion was confirmed by agarose gel electrophoresis. Approximately 6 µg of chromatin, diluted in 400 µl of ChIP buffer, was used per immunoprecipitation; CREB3L2-bound DNA was immunoprecipitated by overnight incubation at 4ºC with anti-CREB3L2 serum (2 µg; HPA015068, Atlas Antibodies). Immunoprecipitates were captured using Protein G magnetic beads and washed with low- and high-salt buffers, as directed. Elution was performed at 65ºC and 1,200 rpm for 30 minutes using a thermomixer, protein-DNA cross-links reversed by treatment with Proteinase K for 2 hours at 65ºC, and DNA purification achieved by using a column-based system. ChIP-seq library preparation and sequencing reactions were conducted at GENEWIZ, Inc. (South Plainfield, NJ, USA). During library preparation, immunoprecipitated samples were normalized to input DNA, i.e., chromatin cross-linked and fragmented side by side with immunoprecipitated DNA using the same conditions. The sequencing libraries were multiplexed and clustered on one lane of a flowcell. Sequencing was performed using a 2x150 Paired-End (PE) configuration. Image analysis and base calling were conducted by the HiSeq Control Software (HCS). Raw sequence data generated from Illumina HiSeq were converted into fastq files and de-multiplexed using Illumina's bcl2fastq v2.17 software. One mismatch was allowed for index sequence identification. ChIP-seq sequencing data were processed and analyzed within the Galaxy web platform, using the public server at usegalaxy.org. First, we run FastQC to evaluate overall sequencing quality (unique reads > 90%). Second, library adapters and low-quality reads were removed using Trimmomatic v0.36. Third, reads were mapped to the hg38 reference genome with Bowtie v0.12.7, and non-uniquely mapped reads filtered out. Forth, unmapped and low quality (MAPQ < 20) reads were excluded with samtools v1.2. Fifth, peak calling was performed with MACS2 v2.1.1 with minimum false discovery rate (FDR) cutoff for peak detection fixed at 0.05, lower and upper mfold bounds defined as 5 and 50, respectively, and extension size set at 144. Finally, peaks were exported to the UCSC genome browser for visualization after conversion to bigwig format. CREB3L2 gene ontology (GO) term enrichment was analyzed using ClueGO v2.5.4 within the Cytoscape platform (v3.6.1).

CREB3L2 and ATF4 aZIPs: reagent preparation and neuronal viability analysis

Specific aZIPs sequences were synthesized by Genewiz. CREB3L2 aZIP: 5'-GGATCCGCCACCATGGACT-ACAAAGATGATGACGACAAGCACATGGCCAGCATGACCGGGGGCCAGCAGATGGGAAGAGACC
CTGATTTGGAACAAAGGGCAGAGGAGCTGGCCCGGGAGAACGAAGAACTGGAGAAGGAAGCTG

AGGAACTTGAGCAGGAGCTCGCTGAACTTCGGAAGAAGGTGGAGGTGCTGGAGAACACCAACAG
GACTCTCCTTCAGCAACTTCAGAAGCTTCAGACTTTGGTGATGGGGAAGGTCTCTCGAACCTGCA
AGTTAGCTGGTACACAGACTGGCACCTGCCTCATGGTCGTTGTGCTTTAAGAATTC-3’. ATF4 aZIP:
5’-GGATCCGCCACCATGGACTACAAAGATGATGACGACAAGCACATGGCCAGCATGACCG-
GGGGCCAGCAGATGGGAAGAGACCCTGATTTGGAACAAAGGGCAGAGGAGCTGGCCCGGGAGA
ACGAAGAACTGGAGAAGGAAGCTGAGGAACTTGAGCAGGAGCTCGCTGAACTCACTGGCGAGTG
TAAAGAGCTAGAAAAGAAGAACGAGGCTCTGAAAGAGAAGGCAGATTCTCTCGCCAAAGAGATT
CAGTATCTAAAAGACCTGATAGAAGAGGTCCGTAAGGCAAGGGGGAAGAAGAGAGTTCCTTAAG
AATTC-3’. aZIP transgenes were were cloned into the modified FUGW plasmid described in ‘Chemically induced proximity: reagent preparation’ using BamHI + EcoRI sites. Lentiviral particles were delivered at DIV1, 24 hours post-dissection. Neuronal cultures were allowed to mature until DIV15 and treated with $A\beta_{42}$ at 750 nM for 48 hours. Cell viability was assayed using RealTime-Glo MT Cell Viability Assay (Promega) following manufacturer’s guidelines; reaction was allowed to proceed for 1 hour before taking luminescence readings (SpectraMax iD5, Molecular Devices).
